# The genome sequence provides insights into salt tolerance of *Achnatherum splendens* (Gramineae), a constructive species of alkaline grassland

**DOI:** 10.1111/pbi.13699

**Published:** 2021-09-15

**Authors:** Guangpeng Ren, Yanyou Jiang, Ao Li, Mou Yin, Minjie Li, Wenjie Mu, Ying Wu, Jianquan Liu

**Affiliations:** ^1^ State Key Laboratory of Grassland Agro‐Ecosystems Institute of Innovation Ecology & School of Life Sciences Lanzhou University Lanzhou China; ^2^ Key Laboratory of Bio‐Resources and Eco‐Environment of the Ministry of Education & State Key Lab of Hydraulics & Mountain River Engineering College of Life Sciences Sichuan University Chengdu China

**Keywords:** *Achnatherum splendens*, genome assembly, whole‐genome duplication, transcriptome, stress tolerance

## Abstract

*Achnatherum splendens* Trin. (Gramineae) is a constructive species of the arid grassland ecosystem in Northwest China and is a major forage grass. It has good tolerance of salt and drought stress in alkaline habitats. Here, we report its chromosome‐level genome, determined through a combination of Illumina HiSeq sequencing, PacBio sequencing and Hi‐C technology. The final assembly of the ~1.17 Gb genome sequence had a super‐scaffold N50 of 40.3 Mb. A total of 57 374 protein‐coding genes were annotated, of which 54 426 (94.5%) genes have functional protein annotations. Approximately 735 Mb (62.37%) of the assembly were identified as repetitive elements, and among these, LTRs (40.53%) constitute the highest proportion, having made a major contribution to the expansion of genome size in *A. splendens*. Phylogenetic analysis revealed that *A. splendens* diverged from the *Brachypodium distachyon*–*Hordeum vulgare*–*Aegilops tauschii* subclade around 37 million years ago (Ma) and that a clade comprising these four species diverged from the *Phyllostachys edulis* clade ~47 Ma. Genomic synteny indicates that *A. splendens* underwent an additional species‐specific whole‐genome duplication (WGD) 18–20 Ma, which further promoted an increase in copies of numerous saline–alkali‐related gene families in the *A. splendens* genome. By transcriptomic analysis, we further found that many of these duplicated genes from this extra WGD exhibited distinct functional divergence in response to salt stress. This WGD, therefore, contributed to the strong resistance to salt stress and widespread arid adaptation of *A. splendens*.

## Introduction

Soil salinity, which is one of the major environmental stresses, limits plant growth (Mahajan and Tuteja, 2005; Zhu, 2001). It has been reported that ∼20% of global land plants are seriously affected by salinity (Morton *et al*., 2019; Qadir *et al*., 2014). High concentrations of salt trigger ion imbalance and hyperosmotic stress in plants, and possibly cause secondary stresses such as oxidative damage (Møller and Tester, 2007; Shabala and Cuin, 2008; Zhu, 2001). These stresses eventually lead to poor growth or plant death. Based on their responses to soil salinity, plants can be divided into glycophytes and halophytes. While glycophytes are highly sensitive to salinity, halophytes can survive in saline–alkali habitats (Flowers and Colmer, 2008; Flowers *et al*., 2010; Gong *et al*., 2020). It is, therefore, necessary to understand the genomic make‐up of halophytes, which may be helpful in the management and usage of these special wild resources (Wu *et al*., 2012).

**Figure 1:**
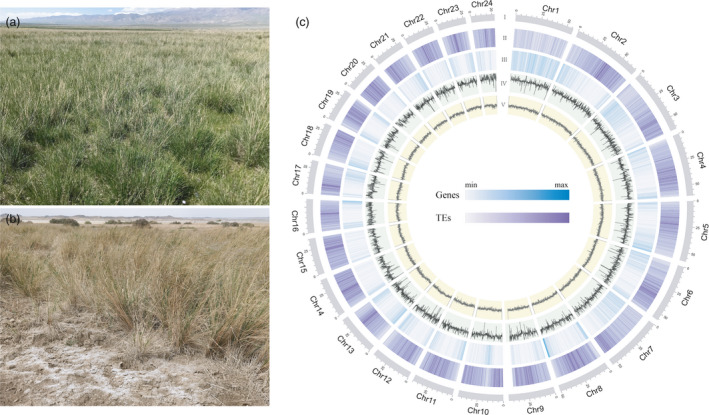
(a) and (b) Photographs of *A. splendens* as a constructive species of the alkaline grassland. (c) The landscape of assembly and annotation of the *A. splendens* genome. Tracks from outside to the inside correspond to I, pseudochromosomes; II, repeat density; III, gene density; IV, depth density and V, GC density.

*Achnatherum splendens* Trin. (Gramineae) (2n = 48; Guo *et al*., 2003) is a perennial halophyte, which is a constructive species of the alkaline grassland in northwest China (*e.g*. Figure 1a; Huai *et al*., 2008; Jiang *et al*., 2017). Through long‐term adaptation to the extremely alkaline environment, this species has developed a considerable level of resistance to salt and drought stress (*e.g*. Figure 1b; Haixia *et al*., 2008; Irfan *et al*., 2016). In addition, the highly developed underground roots of this species help to improve sand fixation and water conservation in saline areas (Zhang *et al*., 2010). This constructive species also provides a major forage resource with good palatability and high nutritive value for local livestock in the arid alkaline regions, and its stalks have also been used as superior raw material for paper making for a long time (You *et al*., 2020).

In this study, we assembled *de novo* a chromosome‐scale genome for *A. splendens* through a combination of various sequencing strategies, involving Illumina HiSeq sequencing, single‐molecule real‐time (SMRT) sequencing and chromosome conformation capture (Hi‐C) technology. RNA‐seq‐based transcriptomics were used to identify differential expressions of the paralogous genes under salt treatments. Using gene annotation, phylogenetic analysis and transcriptomic analysis, we aimed to clarify the genomic basis of how this species adapts to alkaline habitats.

## Results

### Genome sequencing, size estimation and assembly

We estimated the genome size based on K‐mer distribution. In total, 57 879 333 379 k‐mers (size 17) were identified. The major peak was around a k‐mer depth of 52, and another clear peak was detected as being located at half of the expected depth, and inferred to be a heterozygous peak. The genome size of *A. splendens* was estimated to be 1113 Mb (Figure S1 and Table S1).

A total of 83.81 Gb of subreads (75× genome coverage) from the PacBio Sequel platform and 142.82 Gb of short reads (128× genome coverage) from the Illumina HiSeq platform were generated, representing almost 200‐fold coverage of the *A. splendens* genome (Table S2). Contigs were first self‐corrected and initially assembled with high‐quality PacBio subreads, and then polished using the Illumina reads. The improved contigs were further assembled into scaffolds with a scaffold N50 of 1.08 Mb. To assign scaffolds to the correct chromosomal positions, a total of 123.42 Gb Hi‐C reads were produced based on genomic proximity. 94.85% of the whole‐genome assembly was successfully anchored into 24 pseudochromosomes and 92.28% was ordered (Figure S2 and Table S3). The final assembled genome size was 1178 Mb with a super‐scaffold N50 length of 40.3 Mb (Table 1). This is little larger than the predicated genome size, probably as a result of the comparatively high heterozygosity of the genome.

**Table 1:** Features of the *A. splendens* genome assembly

Genome assembly	Value
Estimated genome size (Gb)	1.13
Total length of scaffolds (Gb)	1.17
Number of scaffolds	2297
N50 of scaffolds (Mb)	1.08
N90 of scaffolds (kb)	271.02
Longest scaffolds (Mb)	6.08
Average scaffold length (kb)	513.07
Anchored to chromosome (Gb)	1.12
N50 of super‐scaffold (Mb)	40.3
GC content (%)	45.5
Undetermined bases (%)	0.66
Genome annotation	
Repetitive sequences (Mb)	731.95
Number of protein‐coding genes	57 374
Genes in pseudochromosomes	50 069 (87.30%)
Number of non‐coding RNAs	289 938

The completeness and accuracy of the assembled genome were assessed by BUSCO and RNA sequencing. We found that 1401 of 1440 (97.2%) conserved protein genes were completely captured in the genome (Table S4). Moreover, 74 546 isoforms were generated by 1.37 Gb of PacBio full‐length cDNA sequencing data (Table S5), approximately 98.9% (73 770) of which could be well aligned to the genome (Table S6). These assessments indicate that the genome of *A. splendens* was well assembled, with high quality, completeness and accuracy.

### Genome annotation

A total of ~732 Mb (62.56%) of the *A. splendens* genome assembly was identified as repetitive elements based on *de novo* and homology‐based methods (Figure 1c and Table S7). As in most plant genomes, long terminal repeat retrotransposons (LTR‐RTs), accounting for 40.53% of the genome, are the most abundant elements. Among the LTR‐RTs, *Gypsy* and *Copia* are the most common super‐families, representing 27.4% and 12.4% of the total assembly respectively (Table S7). The ratio of *Gypsy* elements to *Copia* elements is 2.23, similar to that in other species in the Gramineae, except for *O. sativa* which has a ratio of 5.06 (Table S8).

For gene annotation, a high‐confidence set of 57 374 protein‐coding genes was predicted by integrating results from *de novo*, homology‐based and transcript‐based approaches (Table S9), and of these, 87.3% (50 069) genes were anchored into 24 pseudochromosomes. On average, protein‐coding genes in the *A. splendens* genome are 3250 bp long and cover 4.85 exons, and these features are similar to those in other species in the Gramineae (Figure S3; Table S9). The predicted genes were then annotated for functional proteins they encode by a consensus method, applying InterPro (Hunter *et al*., 2009), Gene Ontology (GO; Ashburner *et al*., 2000), Kyoto Encyclopedia of Genes and Genomes (KEGG; Kanehisa and Goto, 2000) and Swiss‐Prot (Boeckmann *et al*., 2003). In the aggregate, 54 426 genes (94.8% of the predicted genes) were identified as having known homologs in protein databases (Table S10). In addition, we detected 644 microRNA (miRNA), 898 transfer RNA (tRNA), 885 ribosomal RNA (rRNA) and 447 small nuclear (snRNA) genes in the genome sequence (Table S11).

### Comparative genomic analysis

**Figure 2:**
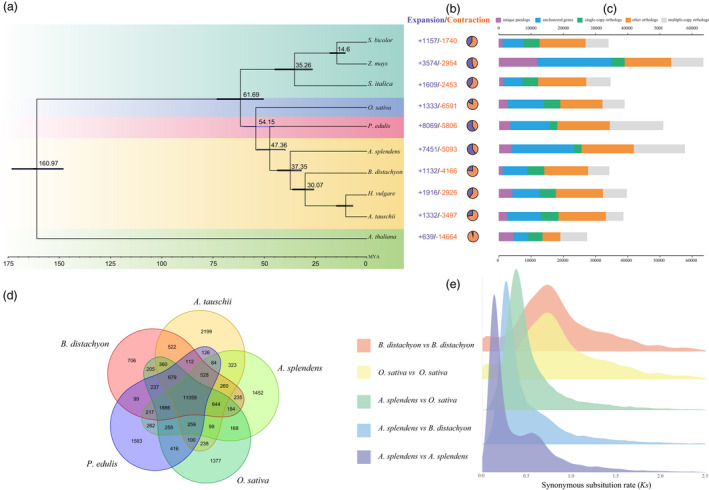
(a) Phylogenetic relationships and times of divergence between *A. splendens* and other Gramineae species. (b) Expansions and contractions of gene families. Purple and yellow indicate expanded and contracted gene families respectively. (c) Clusters of orthologous and paralogous gene families in *A. splendens* and other Gramineae species. Only the longest isoform for each gene is shown. (d) Venn diagram of orthologous genes shared among the five species *A. splenden*s, *O. sativa*, *B. distachyon*, *P. edulis* and *A. tauschii*. © Distribution of average synonymous substitution levels (*K*s) between syntenic blocks.

Based on sequence homology, a total of 310 214 genes were clustered into 177 606 families, of which 7129 families were shared by all 10 species and 1116 were specific to the *A. splendens* genome (Figure 2c,d and Table S12). These unique gene families were predicted to have functions involved in ‘response to oxidative stress’ (GO:0006979) and ‘oxidoreductase activity, acting on peroxide as acceptor’ (GO:0016684) (Table S13).

To infer the phylogeny of *A. splendens*, 963 single‐copy orthologous genes from 10 species were identified and used to construct a phylogenetic tree. The resulted phylogeny indicated that within the monocot lineage comprising six species (*O. sativa*, *P. edulis*, *A. splendens*, *B. distachyon*, *H. vulgare* and *A. tauschii*), the subclade containing only *A. splendens* diverged from the other one comprising the other three species (*B. distachyon*, *H. vulgare* and *A. tauschii*), which together diverged from *P. edulis* and further from *O. sativa*. Based on the MCMCtree and fossil calibration, the estimated divergence times were around 54.16 million years ago (Ma) between *O. sativa* and the other five monocots, 47.36 Ma between *P. edulis* and the remaining four species and 37.35 Ma between *A. splendens* and the other three species (Figure 2a). These divergences were in agreement with estimates from previous reports (Varshney *et al*., 2017).

We identified 7451 gene families that had expanded while 5093 had contracted in the *A. splendens* genome compared to the other plant species (Figure 2b). GO analysis revealed that the expanded orthogroups were significantly enriched in the functional terms ‘transmembrane transport’, ‘transmembrane transporter activity’ and ‘adenyl ribonucleotide binding’ (Figure S4 and Table S14). Several markedly expanded gene families were functionally associated with maintenance of intracellular osmolality and pH homeostasis.

### Whole‐genome duplication and chromosome composition

Whole‐genome duplication (WGD) events, which have happened frequently in most plants, are commonly regarded as a major evolutionary force (De Peer *et al*., 2009; Lynch and Conery, 2000; Salmanminkov *et al*., 2016). The additional genetic material provided by WGD enhances the adaptive plasticity of plants and contribute to species diversification and functional innovations (Paterson *et al*., 2004; Tang *et al*., 2008). By revealing preserved ancestral gene order, gene collinearity analysis is crucial in uncovering changes in and evolution of a plant genome (Wang *et al*., 2006). We examined gene collinearity among the *A. splendens*, *B. distachyon* and *O. sativa* genomes. *B. distachyon* and *O. sativa* were chosen as references because both have relatively simple genome structures, with only one WGD, common to all grass species (cWGD), found in them (Paterson *et al*., 2009; Wang *et al*., 2015).

In order to infer intra‐genomic gene collinearity, we identified a total of 15 371 gene pairs in 677 homologous blocks of the *A. splendens* genome. Using the same criteria, we detected 324 and 657 homologous blocks from the *O. sativa* and *B. distachyon* genomes, which contained 3770 and 5602 collinear gene pairs respectively. The largest homologous block in *A. splendens*, with 1211 gene pairs, was located in chromosomes 2 and 3 (Figure S5), while the largest in *O. sativa* and *B. distachyon* had, respectively, 280 and 265 gene pairs (Table S15). Thus, considerably more homologous gene pairs and more complete homologous regions were found in the *A. splendens* genome than in *O. sativa* or *B. distachyon*, suggesting that one or more additional WGD events had occurred in the *A. splendens* genome. For inter‐genomic gene collinearity, there were 1553 homologous blocks involving 35 753 collinear gene pairs between the *A. splendens* and *O. sativa* genomes and 1664 homologous blocks involving 35 923 collinear gene pairs between the *A. splendens* and *B. distachyon* genomes, revealing well‐preserved syntenic features between the *A. splendens* genome and the two reference genomes (Table S15).

**Figure 3:**
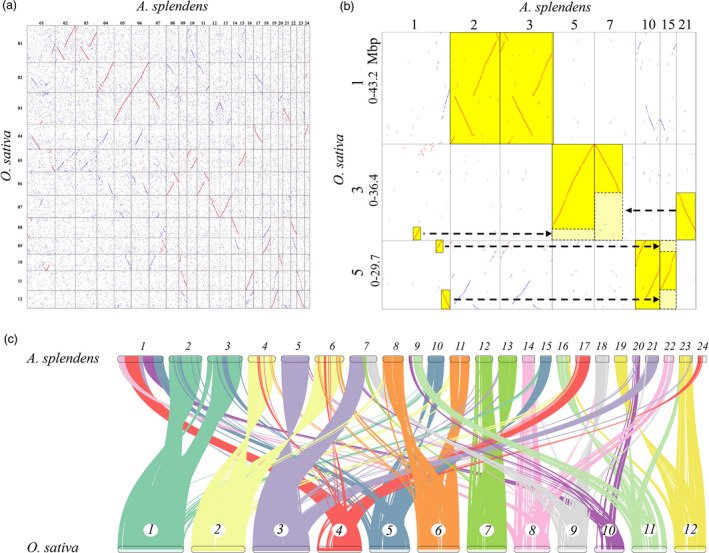
(a) Dotplots showing genes homologous between the *A. splendens* and *O. sativa* genomes. (b) Example of homologous gene dotplots for *A. splendens* and *O. sativa*. Chromosome numbers and regions (in Mbp) are shown. Best hit (orthologous) genes are red dots, secondary hits (outparalogous) are blue dots and the others are shown in grey. Highlights show the best matched chromosomal regions. Arrows show correspondence produced by chromosome breakages during evolution. (c) Gene synteny between the *A. splendens* and *O. sativa* genomes.

**Figure 4:**
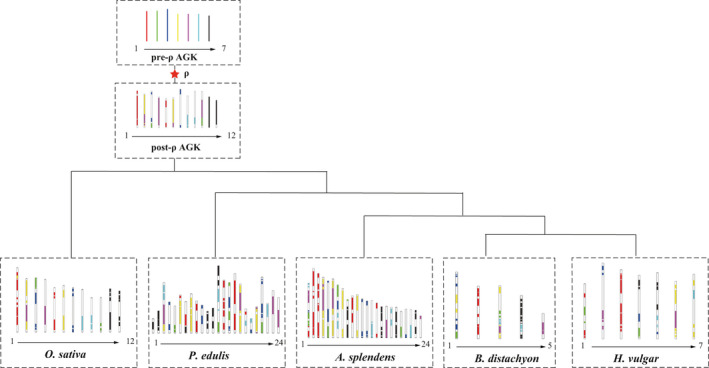
Construction of ancestral chromosomes for *A. splendens*, *O. sativa*, *B. distachyon*, *H. vulgare* and *P. edulis*. AGK indicates the ancestral grass karyotype. The red star indicates a whole‐genome duplication event.

Through mapping the *O. sativa* genome sequence onto the *A. splendens* genome, we generated a homologous gene dotplot (Figure 3), which was helpful in locating homologous correspondence and determining orthologs (established from the *O. sativa*––*A. splendens* split) and outparalogs (established after the cWGD). Without taking into account any gene loss, if there was no additional WGD in *A. splendens* after the cWGD, we would expect to find each *O. sativa* gene (or chromosomal region) having one best matched orthologous *A. splendens* gene (or chromosomal region), and one outparalogous *A. splendens* gene (or chromosomal region). If there had been an *A. splendens*‐specific WGD, we would expect to find each *O. sativa* gene (or chromosomal region) having two best matched orthologous *A. splendens* genes (or chromosomal regions), and two outparalogous *A. splendens* genes (or chromosomal regions). In the dotplots and genomic synteny between *O. sativa* and *A. splendens* (Figure 3), we found that almost every rice chromosome had two best matched chromosomal regions in *A. splendens*. For instance, *O. sativa* chromosomes 1 and 5 are homologous (paralogous) after the cWGD event (Figure 4) and we could identify two more orthologs and two more outparalogs in *A. splendens* (Figure 3b). *O. sativa* chromosome 1 is best matched with (or orthologous to) regions in the *A. splendens* chromosomes 2 and 3, and each orthologous region is outparalogous to *O. sativa* chromosome 5. Using the same strategy, we drew homologous dotplots between *A. splendens* and *B. distachyon*, which exhibited a pattern analogous to that for *O. sativa* (ortholog ratio 1 : 2), further supporting an additional WGD in *A. splendens* (Figure S6).

Furthermore, the synonymous substitution divergence (*K*s) of each collinear gene pair within a genome or between genomes was characterized. The *K*s distribution of paralogs within *O. sativa* and *B. distachyon* suggested only one peak at *K*s ~0.6. In contrast, the paralogous peaks in *A. splendens* showed a clear bimodal feature, having one peak at *K*s ~0.15 and another at ~0.5 (Figure 2e), implying at least two WGD events. The paralogous peak at ~0.6 corresponds to the cWGD event, which has been dated to ~96 Ma (Wang *et al*., 2015). Based on this calibrated time, the *A. splendens*‐specific WGD event was inferred to have occurred ~18–20 Ma. The orthologous peaks for *A. splendens*–*O. sativa* and –*B. distachyon*, corresponding to *K*s values of ~0.45 and ~0.3, were dated to 50 and 35 Ma, respectively, consistent with phylogenetic results.

Genomes often underwent massive gene loss and chromosomal reorganization after WGD events (Wang *et al*., 2005, 2009). Previous studies have suggested that Gramineae genomes evolved from a pre‐ρ ancestral grass karyotype (AGK) with 7 protochromosomes to a post‐ρ AGK with 12 protochromosomes (Murat *et al*., 2010, 2017). To unmask the details of chromosomal reorganizations in *A. splendens*, the AGK genes were downloaded and mapped onto the chromosomes of five Gramineae species (*A. splenden*s, *O. sativa*, *B. distachyon*, *P. edulis* and *H. vulgare*) (Figure 4). It is obvious that *O. sativa* is most similar to the ancestral chromosomal composition; ancestral chromosomes 1, 6 and 7 were well conserved and duplicated to generate chromosomes 1 and 5, 8 and 9, and 11 and 12 respectively. In *A. splendens*, apart from six chromosomes (1, 4, 5, 6, 7 and 9) that had experienced chromosome fusions, each chromosome originated from only one ancestral chromosome, suggesting that there was little chromosomal rearrangement after the additional WGD event in *A. splendens*. In addition, each species examined here inherited one relatively conserved chromosome (AGK 1); for example, it was present as chromosomes 1 and 5 in *O. sativa*, chromosomes 7, 9, 14 and 16 in *H. vulgare*, chromosomes 2, 3, 5 and 15 in *A. splendens*, chromosome 2 in *B. distachyon* and chromosome 3 in *H. vulgare*. It is likely that genes on this conserved chromosome may have played a significant role in major aspects of development and regulation and have therefore avoided recursive chromosomal fusion and fission.

### WGD contributed to adaptation to the saline–alkali environment

In addition to a common WGD event shared by all monocots and another common WGD event shared by Gramineae species (Paterson *et al*., 2004), *A. splendens* experienced an additional species‐specific WGD event after its divergence from other Gramineae species (see above results). Although massive gene loss may have occurred after these WGD events, we still found that more than 55% of the annotated 57 374 genes were duplicate genes in the WGD regions (Table S16). Especially, most of these duplicate genes resulted mainly from the species‐specific WGD event (Figure 3 and Figure S6). To investigate whether this extra WGD event had contributed to the adaptation of *A. splendens* to a saline–alkali environment, we first, based on the functional annotations in the *A. thaliana* genome, identified gene families that are closely related to resistance to salt stress and transcription factor (TF) families in the *A. splendens*, *O. sativa* and *B. distachyon* genomes. We found that copy numbers of the salt‐tolerant–related gene families and TF families in *A. splendens* were, respectively, 1.77 and 1.73 times more than those in *O. sativa*, and 1.77 and 1.67 times more than those in *B. distachyon* (Table S17 and Table S18). The increase in copy number of these gene families may have played a role in the evolution of salt tolerance in *A. splendens*.

**Figure 5:**
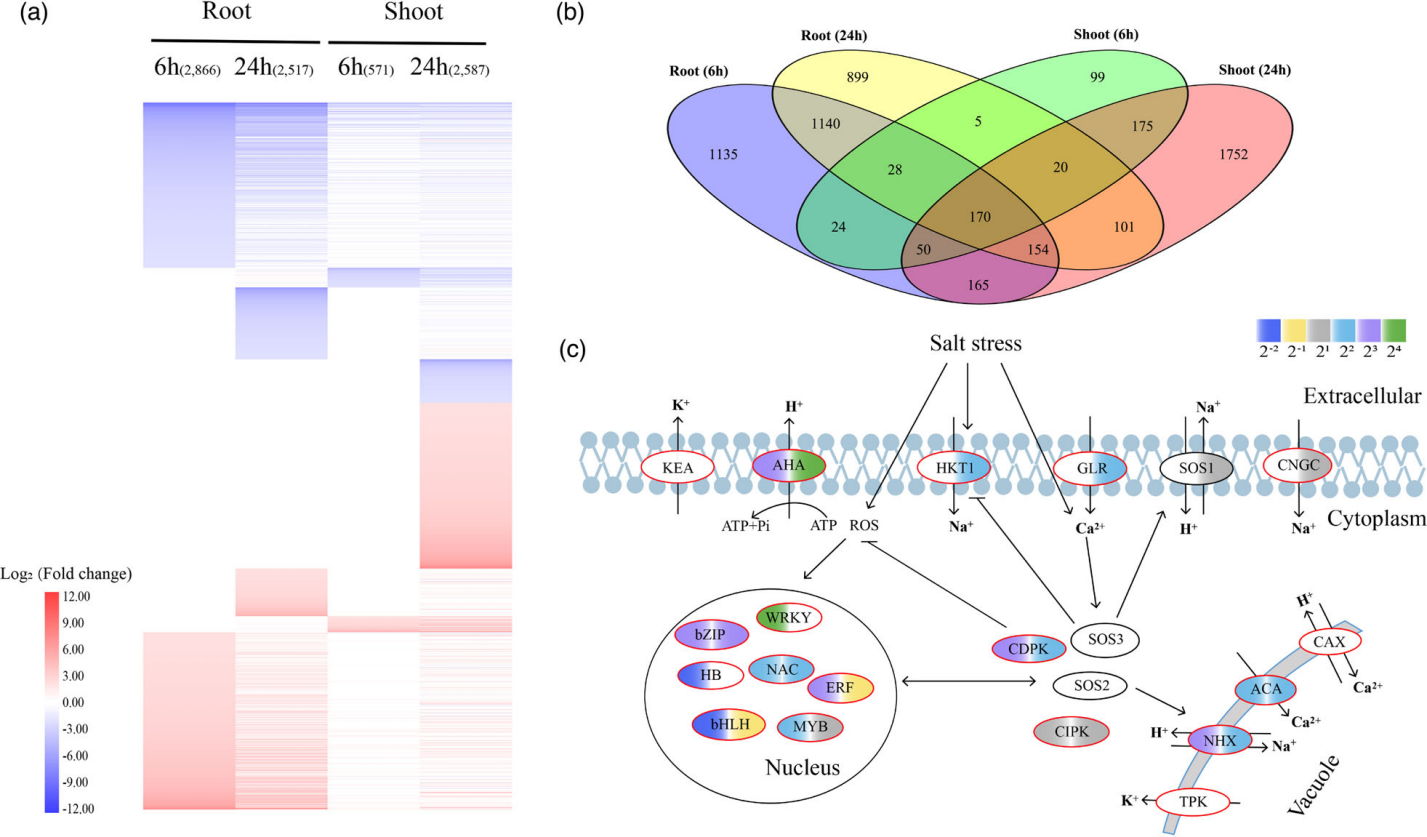
Transcriptomics of *A. splendens* under salt stress. (a) Expression of DEGs identified in root and shoot at each time point. The heatmap was generated from hierarchical cluster analysis of genes. (b) Venn diagram of the number of DEGs in root and shoot at each time point. (c) Expression of key salt tolerance‐related genes in *A. splendens*. Ellipses with black borders indicate single gene, while red borders indicate gene or TF families. For each family, we only selected one copy to show its expression under salt treatment. Detailed expression information of these family members is shown in Table S21. The filled colours correspond to their degree of regulation (TPM_treatment_/TPM_control_) at 6 h in root (left) and at 24 h in shoot (right) in response to salt stress.

**Figure 6:**
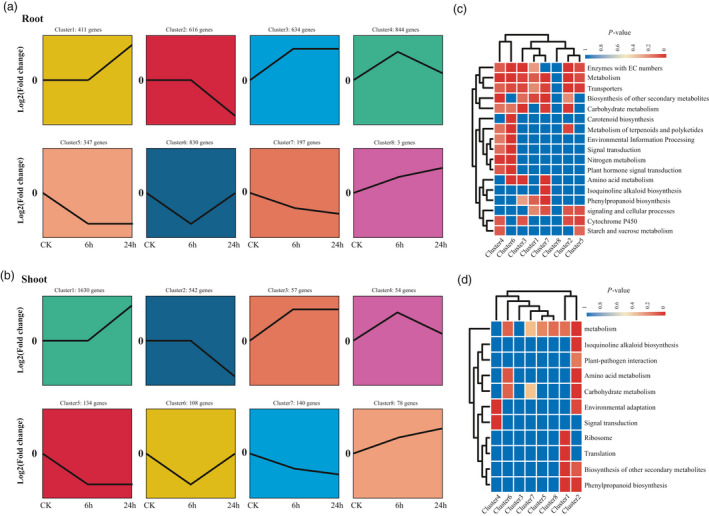
Expression patterns and enrichment analysis of DEGs under salt stress. (a–b) Cluster analysis of DEGs displaying a log_2_ fold change (with absolute value >2) of transcripts during salt stress at 6 and 24 h in root and shoot respectively. The comparisons include control *versus* control (CK), 6 h *versus* control (6 h) and 24 h *versus* control (24 h). (c–d) Heat maps of significantly enriched pathways during salt stress. The blue and red colours indicate the *P*‐values for significantly enriched pathways for each cluster.

Hierarchical cluster analysis of transcriptomic data showed clustering of the four repeats for the shoot or root at each time point (Figure S7), except for one shoot sample at 24 h, which was discarded for downstream analysis. Differentially expressed genes (DEGs) were identified under salt treatment by comparing each time point (6 and 24 h) with 0 h. A total of 2866, 2517, 571 and 2587 DEGs were identified in the 6 h root, 24 h root, 6 h shoot and 24 h shoot respectively (Figure 5a). Only 571 DEGs were detected under salt treatment of 6 h shoot, while more than four times DEGs were identified in 24 h shoot compared to 6 h shoot, indicating that the early stage of salt stress does not considerably affect the shoot of *A. splendens*. Of the total 5917 DEGs, majority of them were specific to each of the four samples and only 170 DEGs were shared by all samples (Figure 5a,b). Co‐expression analysis showed that the DEGs formed eight major clusters in root and shoot respectively (Figure 6). The patterns of some clusters may be correlated with the adaptation to a saline–alkali environment of this species.

In root, cluster 4 (844 DEGs) and cluster 6 (830 DEGs; Figure 6a) are the two biggest modules containing up‐regulated and down‐regulated genes respectively. DEGs of both clusters were induced only at 6 h after salt treatment and are mainly enriched in metabolism of carbohydrate, terpenoids, polyketides and nitrogen, environmental information processing, signal transduction and plant hormone signal transduction. Therefore, phytohormone signals and metabolism processes were turned on or off at the early phase of salt stress. Pathways that play an important role in response to abiotic stresses, such as cytochrome P450, metabolism of amino acid, starch and sucrose and phenylpropanoid biosynthesis, were also enriched by other clusters (Figure 6c). In shoot, the biggest cluster 1, containing 1630 DEGs, was up‐regulated in the late phase (24 h). It contains genes for ribosome, translation and biosynthesis of other secondary metabolites and phenylpropanoid biosynthesis. The second biggest cluster (cluster 2: 542 DEGs) was also induced in the late phase, comprising genes enriched in metabolism of carbohydrate and amino acid, environmental adaptation and plant–pathogen interaction (Figure 6b,d). Therefore, the major clusters and DEGs revealed in root and shoot were different (Figures 5a and 6b,d), probably in relation to diverse adaptive mechanisms in different tissues.

We then investigated expression patterns of paralogous genes in *A. splendens* under salt treatment. We focused on the paralogous genes that have two copies in *A. splendens* with one synteny copy in *O. sativa*. A total of 6796 paralogous gene pairs were identified. Among them, 5347 (78.68%) pairs of genes had contrasted differential expressions and 1047 (15.41%) pairs had one DEG between different tissues in each pair. A total of 402 pairs of genes with differential expression (Table S19) are enriched in many functional categories that are related to salt stress, such as ‘oxidoreductase activity’, ‘antioxidant activity’, ‘transcription regulator activity’ and ‘response to oxidative stress’ (Figure S8). Within these 402 gene pairs, we further detected the differential expression pattern of two paralogous copies in each pair. We did not consider the expression changes in different time points in order to reduce the number of patterns. We recognized four patterns: pattern I, gene functional redundancy; pattern II, one copy was DEG in a tissue, while the other copy was DEG in a different tissue; pattern III, the two copies were differentially expressed in the same tissue, but in different way (*i.e*. up‐ *vs*. down‐regulation) and pattern IV: a combination of patterns II and III. We found that 258 (64.18%) gene pairs showed pattern I, 108 gene pairs (26.86%) for pattern II, 10 pairs for (2.49%) pattern III and 26 pairs (6.47%) for pattern IV (Table S20). These results indicated that many duplicated paralogous genes from the extra WGD in *A. splendens* had evolved the new functions during its saline–alkali adaptation. Finally, we examined the expression of the duplicated genes, especially in the TF families related to the salt stress response pathway of this species. We found that many duplicate genes of these families, for examples, *bZIP*, *NAC* and *WRKY*, important TFs in the regulation of gene expression in response to abiotic stress and the key genes controlling ion homeostasis (*e.g*. *NHX* and *ACA*) were extensively up‐regulated in the salt‐stressed samples (Figure 5c and Table S21). However, the *SOS* (salt overly sensitive) genes with relative high expression level (Table S21) that are critical for salt tolerance (Gong *et al*., 2020) were not differentially expressed in *A. splendens*, which is consistent with previous result (Liu *et al*., 2016).

## Discussion

Abiotic stress, especially salinity and alkali, severely limits plant growth and biomass productivity worldwide, and this is becoming an enormous threat because of increases in climatic changes and human activities (Yamaguchi and Blumwald, 2005; Zhu, 2001). Previously researchers have conducted genomic analyses of salt‐tolerant plants, but our current knowledge remains limited in the case of constructive forage species of alkaline grassland (Huang *et al*., 2020; Ma *et al*., 2013; Zhu *et al*., 2007). Such key species are likely to have developed more adaptive features than other halophytes. In this study, we assembled a high‐quality genome for *A. splendens*, a constructive species of the alkaline grassland in Northwest China.

We combined Illumina HiSeq sequencing, PacBio sequencing and Hi‐C technology to generate a high‐quality chromosome‐scale genome assembly for *A. splendens*. The final assembled genome size was around 1.17 Gb with a super‐scaffold N50 of 40.3 Mb, and more than 94% of the genome sequences could be located on 24 pseudochromosomes. Frequent activation of repetitive elements is one of the main driving forces that act to increase genome sizes (Flavell *et al*., 1974; Levin and Moran, 2011). A total of 731 Mb was identified as repetitive elements, representing 62.11% of the total genome. LTR‐RTs dominated primarily, as in other plants (Vicient *et al*., 1999). Most LTR‐RTs had expanded and accumulated recently in the *A. splendens* genome. It should be noted that TE activity may also contribute to plant stress responses and, thus, strengthen the adaptation of *A. splendens* to harsh environments (Wendel, 2000). We identified a total of 57 374 protein‐coding genes from the assembled genome of *A. splendens*. This number is greater than those in *O. sativa* (39 049) and *B. distachyon* (34 310), which have not undergone an additional WGD, and other Gramineae species.

whole‐genome duplications or polyploidizations play critical roles in plant adaptations to stressful habitats (Adams and Wendel, 2005; Chen *et al*., 2020; Li *et al*., 2021; Soltis *et al*., 2014; Wang *et al*., 2021). They provide functional innovations through gene duplication, chromosome rearrangements and genome repatterning events (Murat *et al*., 2010). WGDs are, therefore, closely linked to species evolution and diversification. Recent research has revealed that all monocots experienced a common WGD event and that Gramineae species shared another common WGD event (Paterson *et al*., 2004). Genomic analyses indicated that *A. splendens* experienced an additional WGD event after its divergence from other monocots, which was estimated to happen at 18–22 Ma. We also established lists of orthologous and paralogous genes resulting from this recent WGD event, which may be useful for comparative genomics analyses in future research. This WGD may have resulted in the evolution of salt and drought tolerance in *A. splendens* through increasing the copy numbers of gene families followed by functional redundancy or innovations related to the regulation of ionic and osmotic equilibrium and the ABA pathway. For example, we identified numerous pairs of paralogous genes (*i.e*. two copies in *A. splendens vs*. one copy in *O. sativa*) in *A. splendens*, which arose from this species‐specific WGD. We focused on the duplicate genes that both are identified as DEGs in each pair. We identified a total of 402 pairs of such duplicate genes, some of which are enriched in response to oxidative stress and cell wall organization or biogenesis, suggesting that these duplicated genes should have played an important role in response to salt stress of this species. Of these 402 paralogous gene pairs, more than 35% gene pairs exhibit functional divergence by differential expression in different tissues or in different ways. Such an increase in gene family members and their functional redundancy or innovations may have enhanced the adaptation of this constructive species to large‐scale alkaline environments. The WGD‐driven salt resistance mechanisms revealed for this species differ from those in other genome‐sequenced halophytes, in which tandem copy increases in gene family members and positive mutations in genes involved in salt stress have enhanced their salt tolerance (Huang *et al*., 2020; Ma *et al*., 2013; Zhu *et al*., 2007). In summary, the availability of a high‐quality genome and transcriptomic data under salt treatment for *A. splendens* provides genomic insights into salt adaptation in this species and new genetic resources for evolutionary and comparative genomics analyses with other monocots in the future.

## Methods

### Plant material and growth conditions

One live *A*. *splendens* plant was collected from Xining, Qinghai Province, China, and then transferred to a greenhouse at the State Key Laboratory of Grassland and Agro‐ecosystems, School of Life Sciences, Lanzhou University. This plant was grown under controlled temperature (24 ± 1 °C) and humidity (45%–55%) conditions with a 14 h−10 h day−night cycle and used for genomic sequencing. Seeds of *A. splenden*s from the same population were collected and used for karyotype analysis and RNA‐seq analysis under salt treatment. Seeds were first treated with a 4% sodium hypochlorite solution for 15 min, then rinsed thoroughly with sterile water. After chilled at 4 °C for 2 days, seeds were put in moist Petri dishes to germinate at 25 °C until the radicals were approximately 2 cm. Then, the seedlings were transferred into plant cultivation boxes (96 holes, aperture 5 mm) with 1 litre of MS (Murashige and Skoog Basal Medium with Vitamins, Phyto Technology Laboratories, Usage: 4.43 grams per litre) fluid nutrient medium, and grown in greenhouse under the same controlled conditions as the live *A. splendens* plant. The 4‐week‐old seedlings were treated with 300 mm NaCl (Liu *et al*., 2016). Roots and shoots with salt treatment for 0, 6 and 24 h were collected, immediately frozen in liquid nitrogen and stored at −80 °C. For each time point, ten seedlings from one cultivation box were pooled and used as a single sample. There were four biological replicates for each sample.

### DNA extraction, genome and Hi‐C sequencing

To overcome the difficulty caused by the high heterozygosity and repetitive sequence content of the *A. splendens* genome (see Results), a combination of three different sequencing strategies (short read from Illumina, subread from PacBio and interaction read from Hi‐C) was employed to obtain a high‐quality reference genome. Genomic DNA was extracted by a standard CTAB method (Porebski *et al*., 1997) from fresh leaves which were harvested and frozen immediately in liquid nitrogen. Selected high‐quality DNA samples were sent to BGI for both Illumina and PacBio sequencing. For the Illumina sequencing, a library with an insert size of 270 bp was constructed and sequenced using an Illumina HiSeq 4000 platform (Illumina, San Diego, CA) to produce a short‐read sequencing library. The 20‐kb PacBio SMRT libraries were prepared according to the manufacturer’s standard protocol and sequenced on a PacBio‐sequel platform (PacBio, Menlo Park, CA) with 18 cells. Through mapping interactions between chromatin regions, the Hi‐C approach has been proved to be more effective than traditional genetic mapping for genome assembly, especially in terms of specificity, coverage and cost. A Hi‐C experiment was conducted according to a published procedure that briefly included cell cross‐linking, endonuclease digestion, terminal repair, cyclization, DNA purification and capture. We generated a total of 120 Gb of clean Hi‐C data (Table S2) from the Illumina HiSeq instrument, accounting for 100× coverage of the estimated *A. splendens* genome size. All sequencing reads were trimmed to remove adaptors and enhance quality.

### Transcriptome sequencing

Fresh tissues of the same plant of *A. splendens* (leaf, stem, flower, seed and root) were mixed and sampled for PacBio full‐length cDNA sequencing. Two ISO‐Seq libraries were constructed for the mixed sample from these tissues and sequenced in two cells with a Pacific Bioscience RS II platform, generating 1 371 202 253 raw data (475 753 reads) and details are summarized in Table S3. For salt‐treated samples, total RNA extraction, library construction and sequencing were performed by BGI‐Shenzhen Company (Wuhan, China) on the MGI2000 platform by 2 × 150 bp pair‐end mode.

### Genome size estimation

Genome size was estimated by K‐mer frequency distribution analysis (Genome Size = K‐mer_num/Peak_depth) (Li *et al*., 2010). In order to calculate and plot the K‐mer frequency distribution, a total of 67.28 Gb Illumina short reads were used to determine the total number of k‐mers of length 17 by means of jellyfish (Marçais and Kingsford, 2011). The selection of 17 mers to plot the frequency distribution was based on genome characteristics and the pattern of the Poisson distribution (Figure S1).

### Genome assembly

The PacBio reads, corrected by CANU v1.8 (Koren *et al*., 2017), were first applied to generate the primary contigs via FALCON v3.1 (Chin *et al*., 2016) with the parameters length_cutoff = 15kb, length_cutoff_pr = 15kb, pa_HPCdaligner_option = ‘‐v ‐B128 ‐t16 ‐w8 ‐M32 ‐e0.75 ‐k16 ‐T32 ‐h320 ‐l3200 ‐s1000’ and ovlp_HPCdaligner_option = ‘‐v ‐B128 ‐k16 ‐T32 ‐h480 ‐e0.96 ‐l1600 ‐s1000’. Subsequently, contigs were enhanced by mapping all filtered PacBio reads through QUIVER (Chin *et al*., 2013) and were further corrected by Pilon v1.22 (Sun *et al*., 2021; Walker *et al*., 2014) with default parameters to obtain the consensus sequences. These improved contigs were then assembled into scaffolds by SSPACE‐LongRead v1.1 (Boetzer and Pirovano, 2014). Finally, potential duplicate haplotypes were removed based on PURGE HAPLOTIGS v1.1.0 with the option ‘‐a 80’ (Roach *et al*., 2018).

### Chromosome assembly using Hi‐C data

For chromosome‐level assembly, the Hi‐C data were first aligned to the scaffolds by BWA v0.7.17 (Li and Durbin, 2009). They were classified as valid interaction pairs or invalid interaction pairs using HiC‐Pro v2.11.1 (Servant *et al*., 2015), and only valid interaction pairs were retained for subsequent assembly (Sun *et al*., 2021). Any two segments that presented connections inconsistent with the information from the raw contig were corrected manually. Furthermore, scaffolds were divided into subgroups and sorted and oriented into 24 pseudochromosomes using LACHESIS (Burton *et al*., 2013) with parameters CLUSTER_MIN_RE_SITES = 22, CLUSTER_MAX_LINK_DENSITY = 2, CLUSTER_NONINFORMATIVE_RATIO = 2, ORDER_MIN_N_RES_IN_TRUN = 10 and ORDER_MIN_N_RES_IN_SHREDS = 10.

### Assessment of genome assembly

The completeness of the genome assembly was evaluated by both transcriptome data and Benchmarking Universal Single‐Copy Orthologs (BUSCO). Isoforms generated by PacBio full‐length cDNA sequencing were remapped to the final genome assembly by GMAP with default parameters (Wu and Watanabe, 2005). In addition, 1440 conserved single‐copy orthologs in the BUSCO embryophyta odb9 dataset were searched against the genome sequences through BUSCO with the default parameters.

### Repeat annotation

Repetitive elements, including tandem repeats and transposable elements (TEs), were predicted in the *A. splendens* genome. For tandem repeats, Tandem Repeats Finder v4.09 (Benson, 1999) was implemented with the settings Match = 2, Mismatch = 7, Delta = 7, PM = 80, PI = 10, Minscore = 50 and MaxPeriod = 2000. For identification of TEs, a combination of homology‐based and *de novo* approaches was mainly used. We first used RepeatMasker v4.0.5 (Tarailo‐Graovac and Chen, 2009) with the Repbase TE library (Jurka *et al*., 2005) and RepeatProteinMasker (Tarailo‐Graovac and Chen, 2009) with the TE protein database to search for homologous repeat sequences in the genome. Then, *de novo*‐based identification was performed by RepeatModeler v5.8.8 (Tarailo‐Graovac and Chen, 2009) and LTR_FINDER (Xu and Wang, 2007) to predict the repeat element boundaries and family relationships from genome data. Finally, all repeat identification results from the different software packages were integrated and redundancy was eliminated to produce the final repeat annotation.

### Gene annotation

Three complementary methods were employed to predict protein‐coding genes: homology‐based, *de novo* and transcriptome‐based predictions. For homology‐based predictions, protein sequences from seven different species (*A. thaliana*, *O. sativa*, *S. bicolor*, *S. italica*, *Z. mays*, *B. distachyon* and *H. vulgare*) were downloaded and aligned with the repeat‐masked *A. splendens* genome by TBALSTN with an E‐value cut‐off of 1e‐5. The aligned sequences and candidate genomic regions were corrected and optimized by GeneWise v2.4.1 (Birney *et al*., 2004) for further prediction of exact protein coding gene structures. For *de novo* prediction, 3000 full‐length genes were selected randomly from the homology‐based predictions to train gene models of *A. splendens*. Four *de novo* prediction programs (Augustus v2.5.5, Stanke *et al*., 2008; Geneid v1.4, Blanco *et al*., 2007; GeneMark v3.54, Lukashin and Borodovsky, 1998; SNAP v2006‐07‐28, Korf, 2004) were utilized with *A. splendens* gene models for *de novo* prediction. Genes with coding sequences of <150 bp were discarded. For transcriptome‐based predictions, all RNA‐seq data from mixed samples (flower, leaf, stem, root and seed) were mapped to the *A. splendens* genome by PASA v2.3.3 (Haas *et al*., 2003), then TransDecoder (http://transdecoder.sourceforge.net/) was used to produce the annotation file. Finally, all predictions of gene models yielded by the above approaches were integrated using EVidenceModeler (EVM) v1.1.1 (Haas *et al*., 2008) to generate a consensus gene set.

Functional annotations of the predicted protein‐coding genes were applied by carrying out BLASTP (E‐value 1e‐5) against publicly available protein databases including KEGG (Kanehisa and Goto, 2000), SwissProt (Boeckmann *et al*., 2003) and InterProScan v5.36 (Zdobnov and Apweiler, 2001). GO annotations were accomplished using the Blast2GO pipeline v3.1.3 (Conesa *et al*., 2005).

### Annotation of non‐coding RNAs (ncRNAs)

Transfer RNAs (tRNAs) were predicted by tRNAscan‐SE v1.3.1 (Lowe and Eddy, 1997) with eukaryote parameters. Four types of ncRNA (microRNA, ribosomal RNA, small nuclear RNA and small nucleolar RNA) were identified by Infernal cmscan v1.1.1 (Nawrocki and Eddy, 2013) against the Rfam database v12.0 (Kozomara and Griffiths‐Jones, 2013).

### Gene family clusters and phylogenetic tree reconstruction

To examine evolution and divergence of the *A. splendens* genome, protein‐coding gene sequences from eight species of Gramineae (*Oryza sativa*, *Sorghum bicolor*, *Setaria italica*, *Zea mays*, *Brachypodium distachyon*, *Hordeum vulgare*, *Aegilops tauschii* and *Phyllostachys edulis*) and one eudicot (*Arabidopsis thaliana)* were downloaded from Phytozome v12 (https://phytozome.jgi.doe.gov/pz/portal.html) and the NCBI website (https://www.ncbi.nlm.nih.gov/) for comparative analyses. When one gene had multiple transcripts, only the longest transcript in the coding region was kept for further analysis. An all‐against‐all BLASTP search (Camacho *et al*., 2009) was applied to calculate pairwise sequence similarities with the default settings (E‐value ≤1e‐5 and a minimum match length of 50%). BLASTP results were then clustered into paralogs and orthologs using the OrthoMCL v2.0.9 method (Li *et al*., 2003). A Venn diagram was drawn to show the numbers of shared and species‐specific gene families among five species of Gramineae (*A. splenden*s, *O. sativa*, *B. distachyon*, *P. edulis* and *A. tauschii*).

Single‐copy orthologous genes were extracted from the OrthoMCL clustering results and MUSCLE v3.8.31 (Edgar, 2004) was used to align the protein sequences. Guided by these alignments, protein sequences were transformed into coding DNA sequences (CDS). Fourfold degenerate sites were extracted from CDS alignments and concatenated to give a super‐gene matrix. RAxML v8.1.17 (Stamatakis, 2014) was used to reconstruct a phylogenetic tree with the GTR+G+I model and a bootstrap value of 100. The resulting phylogeny was visualized by iTOL (https://itol.embl.de/).

### Estimation of divergence time

Species divergence time was inferred by MCMCtree in the PAML program v4.5 (Yang, 2007) with the ‘relaxed‐clock (clock = 2)’ model and ‘F84’ model. The divergence times for *A. thaliana*–*O. sativa* (mean: 150.0 Mya, std dev: 4.0) and for *O. sativa*–*Z. mays* (mean: 50.0 Mya, std dev: 4.0) obtained from the TimeTree database (http://www.timetree.org) were applied to calibrate the divergence times.

### Expansion and contraction of gene families

To analyse the expansion and contraction of gene families, the Computational Analysis of gene Family Evolution (CAFÉ) program v3.1 (De Bie *et al*., 2006) was run to compute changes in gene families along each lineage of the phylogenetic tree under a random birth‐and‐death model. The clustering results and the information from the estimated divergence times were used. Using conditional likelihood as the test statistic, the corresponding *P*‐values of each lineage were calculated and a *P*‐value of 0.01 was regarded significant. Additionally, the expanded and contracted gene families were subjected to GO enrichment to determine their functions.

### Genome synteny and whole‐genome duplication

Protein sequences within and between genomes were searched against one another to detect putative homologous genes (*E*‐value < 1e‐5) by BLASTP. With information about homologous genes as input, CollinearScan (Wang *et al*., 2006) and MCscanX (Wang *et al*., 2012) were implemented to infer homologous blocks involving collinear genes within and between genomes. The maximal gap length between collinear genes along a chromosome region was set to 50 genes (Wang *et al*., 2017, 2018). Then, homology dotplots were constructed by a perl script to reveal genomic correspondence. Non‐synonymous (*K*a) and synonymous (*K*s) substitution rates for gene pairs were calculated with KaKs Calculator v2.0 (Wang *et al*., 2010) under a YN model after converting protein alignments into codon alignments by PAL2NAL v14 (Suyama *et al*., 2006).

### Identification of genes related to saline–alkali tolerance

Gene families related to the regulation of stress tolerance were collected manually from published *A. thaliana* papers. Transcription factor families from *A. thaliana* were further downloaded from PlantTFDB v5.0 (Jin *et al*., 2016). All genes of the genomes of *A. splenden*s and related species were first searched against *A. thaliana* by BLASTP with an E‐value cut‐off of 1e‐5, then domains of candidate genes were identified by HMMER v3.2.1 (Finn *et al*., 2011) by searching the Pfam database (Punta *et al*., 2011). Only genes having protein domains identical to those of *A. thaliana* genes were selected as orthologs.

### Differential expression analysis

To examine genome‐wide responses to salt stress of *A. splendens*, we performed transcriptomic analysis under 300 mm NaCl treatment for 6 and 24 h (h) for both shoot and root tissues. For raw sequencing reads, adapters, reads containing poly‐N and lower‐quality reads (<Q30) were removed to obtain clean reads, which were then mapped to the *A. splenden*s genome using HISAT2 (Daehwan *et al*., 2015). StringTie v1.3.1 (Mihaela *et al*., 2015) was used to calculate transcripts per million (TPM) of mRNAs in each sample. TPM of genes was computed by summing the TPMs of transcripts in each gene group. We used DESeq R package v1.10.1 (negative binomial distribution) to perform differential expression analysis. DEGs were defined as those having at least twofold change in expression compared with those in 0 h (false discovery rate, FDR < 0.05). K‐cluster analysis of DEGs was performed using the OmicShare tools (http://www.omicshare.com/tools). Genes in each cluster were subjected to KEGG enrichment analysis.

## Conflicts of interest

The authors declare no competing interests.

## Author contributions

J.Q.L. conceived and designed the project. Y.Y.J. collected the genomic materials. G.P.R. and M.Y. conducted salt treatment experiment and collected the transcriptomic materials. G.P.R. and Y.Y.J. assembled the genome and performed gene annotation, gene family and evolutionary analyses, and genomic analyses with help from W.J.M. and Y.W. A.L. and M.J.L. conducted the transcriptomic analyses. G.P.R., Y.Y.J. and J.Q.L. wrote the manuscript. All authors read and approved the final version of the manuscript.

## Supporting information


**Figure S1** K‐mer analysis of the *A. splendens* genome by using K‐mer = 17.
**Figure S2** Hi‐C‐assisted assembly of *A. splendens* pseudochromosomes.
**Figure S3** Comparison of (a) CDS length, (b) mRNA length, (c) exon length and (d) intron length between *A. splendens* and other six related species.
**Figure S4** Gene ontology (GO) enrichment of the expanded gene families in *A. splendens*.
**Figure S5** Overview of dotplots within the *A. splendens* genome paralogous genes.
**Figure S6** (a) Overview of dotplots between *A. splendens* and *B. distachyon* genome homologous genes. (b) Example of homologous gene dotplots between *A. splendens* and *B. distachyon*. Chromosome numbers and regions (in Mbp) were shown. Best hit (orthologous) genes are red dots, secondary hits (outparalogous) are blue dots and the others are shown in grey. Highlights show the best matched chromosomal regions. Arrows show complement correspondence produced by chromosome breakages during evolution.
**Figure S7** Hierarchical cluster analysis of gene expression in root and shoot tissues.
**Figure S8** Gene ontology (GO) enrichment of the 402 pairs of paralogous genes in *A. splendens*.
**Table S1** Estimation of the *A. splendens* genome size based on 17‐mer statistics.
**Table S2** Summary of DNA sequencing data.
**Table S3** Assembly statistics based on Hi‐C data.
**Table S4** Genome assembly completeness evaluation by BUSCO.
**Table S5** Summary of PacBio full‐length cDNA sequencing.
**Table S6** The length distribution of full‐length (FL) transcripts.
**Table S7** Classification of interspersed repeats in the assembled *A. splendens* genome.
**Table S8** LTR subclass ratio in *A. splendens* and other five Gramineae genomes.
**Table S9** Summary of predicted protein‐coding gene annotations and their supporting evidence types.
**Table S10** Functional annotation of predicted genes in the *A. splendens* genome.
**Table S11** Summary statistics of non‐coding RNAs in the *A. splendens* genome.
**Table S12** Summary of gene family clustering.
**Table S13** Gene ontology (GO) enrichment analysis of the unique families in *A. splendens*.
**Table S14** Gene ontology (GO) enrichment analysis of the expanded families in *A. splendens*.
**Table S15** Number of gene pairs and homologous blocks within six Gramineae genomes or between the *A. splendens* and other five genomes.
**Table S16** Statistics of duplication types of annotated genes in *A. splendens*.
**Table S17** Copy numbers of gene families that are involved in salt–saline tolerance in the *A. thaliana, O. sativa, B. distachyon and A. splendens* genomes.
**Table S18** Copy numbers of transcription factor families identified in the *A. thaliana, O. sativa, B. distachyon* and *A. splendens* genomes.
**Table S19** The present pattern of DEGs in each of the total 6,796 paralogous gene (i.e., two copies in *A. splendens* with one synteny copy in *O. sativa*) pairs in *A. splendens*.
**Table S20** The differential expression pattern of 402 paralogous gene pairs in both root and shoot.
**Table S21** The expression (TPM) of the salt tolerance‐related DEGs in root and shoot of *A. splendens*.

## Data Availability

The whole‐genome sequence data (including Illumina short‐gun reads, PacBio SMRT reads and Hi‐C interaction reads), the PacBio full‐length transcriptomes and salt treatment of RNA‐seq data have been deposited at NCBI under the Bioproject ID: PRJNA755179. The final assembled genome and genome annotations have been deposited in the National Genomics Data Center (https://bigd.big.ac.cn/?lang=en), under accession PRJCA006214.

## References

[pbi13699-bib-0001] Adams, K.L. and Wendel, J.F. (2005) Polyploidy and genome evolution in plants. Curr. Opin. Plant Biol. 8, 135–141. 15752992 10.1016/j.pbi.2005.01.001

[pbi13699-bib-0002] Ashburner, M. , Ball, C.A. , Blake, J.A. , Botstein, D. , Butler, H. , Cherry, J.M. , Davis, A.P. *et al*. (2000) Gene ontology: tool for the unification of biology. The Gene Ontology Consortium. Nat. Genet., 25(1), 25–29. 10802651 10.1038/75556 PMC3037419

[pbi13699-bib-0003] Benson, G. (1999) Tandem repeats finder: a program to pomixe DNA sequences. Nucleic Acids Res. 27, 573–580. 9862982 10.1093/nar/27.2.573 PMC148217

[pbi13699-bib-0004] Birney, E. , Clamp, M. and Durbin, R. (2004) GeneWise and genomewise. Genome Res. 14, 988–995. 15123596 10.1101/gr.1865504 PMC479130

[pbi13699-bib-0005] Blanco, E. , Parra, G. and Guigó, R. (2007) Using geneid to identify genes. Curr. Protoc. Bioinformatics, 18, 4.3.1–4.3.28. 10.1002/0471250953.bi0403s18 18428791

[pbi13699-bib-0006] Boeckmann, B. , Bairoch, A. , Apweiler, R. , Blatter, M.‐C. , Estreicher, A. , Gasteiger, E. , Martin, M.J. *et al*. (2003) The SWISS‐PROT protein knowledgebase and its supplement TrEMBL in 2003. Nucleic Acids Res. 31, 365–370. 12520024 10.1093/nar/gkg095 PMC165542

[pbi13699-bib-0007] Boetzer, M. and Pirovano, W. (2014) SSPACE‐LongRead: scaffolding bacterial draft genomes using long read sequence information. BMC Bioinformatics, 15, 211. 24950923 10.1186/1471-2105-15-211 PMC4076250

[pbi13699-bib-0008] Burton, J.N. , Adey, A. , Patwardhan, R.P. , Qiu, R. , Kitzman, J.O. and Shendure, J. .(2013) Chromosome‐scale scaffolding of de novo genome assemblies based on chromatin interactions. Nat. Biotechnol., 31(12), 1119–1125. 24185095 10.1038/nbt.2727 PMC4117202

[pbi13699-bib-0009] Camacho, C. , Coulouris, G. , Avagyan, V. , Ma, N. , Papadopoulos, J. , Bealer, K. and Madden, T.L. (2009) BLAST+: architecture and applications. BMC Bioinformatics, 10, 421. 20003500 10.1186/1471-2105-10-421 PMC2803857

[pbi13699-bib-0010] Chen, Y. , Ma, T. , Zhang, L. , Kang, M. , Zhang, Z. , Zheng, Z. , Sun, P. *et al*. (2020) Genomic analyses of a “living fossil”: the endangered dove‐tree. Mol. Ecol. Resour. 20, 756–769. 10.1111/1755-0998.13138 31970919

[pbi13699-bib-0011] Chin, C.‐S. , Alexander, D.H. , Marks, P. , Klammer, A.A. , Drake, J. , Heiner, C. , Clum, A. *et al*. (2013) Nonhybrid, finished microbial genome assemblies from long‐read SMRT sequencing data. Nat. Methods, 10, 563–569. 23644548 10.1038/nmeth.2474

[pbi13699-bib-0012] Chin, C.‐S. , Peluso, P. , Sedlazeck, F.J. , Nattestad, M. , Concepcion, G.T. , Clum, A. , Dunn, C. *et al*. (2016) Phased diploid genome assembly with single‐molecule real‐time sequencing. Nat. Methods, 13, 1050–1054. 27749838 10.1038/nmeth.4035 PMC5503144

[pbi13699-bib-0013] Conesa, A. , Götz, S. , García‐Gómez, J.M. , Terol, J. , Talón, M. and Robles, M. (2005) Blast2GO: a universal tool for annotation, visualization and analysis in functional genomics research. Bioinformatics, 21, 3674–3676. 16081474 10.1093/bioinformatics/bti610

[pbi13699-bib-0014] Daehwan, K. , Ben, L. and Salzberg, S.L. (2015) HISAT: a fast spliced aligner with low memory requirements. Nat. Methods, 12, 357–360. 25751142 10.1038/nmeth.3317 PMC4655817

[pbi13699-bib-0015] De Bie, T. , Cristianini, N. , Demuth, J.P. and Hahn, M.W. (2006) CAFE: a computational tool for the study of gene family evolution. Bioinformatics, 22, 1269–1271. 16543274 10.1093/bioinformatics/btl097

[pbi13699-bib-0016] De Peer, Y.V. , Maere, S. and Meyer, A. (2009) The evolutionary significance of ancient genome duplications. Nat. Rev. Genet. 10, 725–732. 19652647 10.1038/nrg2600

[pbi13699-bib-0017] Edgar, R.C. (2004) MUSCLE: multiple sequence alignment with high accuracy and high throughput. Nucleic Acids Res. 32, 1792–1797. 15034147 10.1093/nar/gkh340 PMC390337

[pbi13699-bib-0018] Finn, R.D. , Clements, J. and Eddy, S.R. (2011) HMMER web server: interactive sequence similarity searching. Nucleic Acids Res. 39, W29–W37. 21593126 10.1093/nar/gkr367 PMC3125773

[pbi13699-bib-0019] Flavell, R. , Bennett, M. , Smith, J. and Smith, D. (1974) Genome size and the proportion of repeated nucleotide sequence DNA in plants. Biochem. Genet. 12, 257–269. 4441361 10.1007/BF00485947

[pbi13699-bib-0020] Flowers, T.J. and Colmer, T.D. (2008) Salinity tolerance in halophytes. New Phytol. 179, 945–963. 18565144 10.1111/j.1469-8137.2008.02531.x

[pbi13699-bib-0021] Flowers, T.J. , Galal, H.K. and Bromham, L. (2010) Evolution of halophytes: multiple origins of salt tolerance in land plants. Funct. Plant Biol. 37, 604–612.

[pbi13699-bib-0022] Gong, Z. , Xiong, L. , Shi, H. , Yang, S. , Herrera‐Estrella, L.R. , Xu, G. , Chao, D.‐Y. *et* *al*. (2020) Plant abiotic stress response and nutrient use efficiency. Sci. China Life Sci. 63, 635–674. 32246404 10.1007/s11427-020-1683-x

[pbi13699-bib-0023] Guo, Y.T. , Wei, L.J. and Yan, P. (2003) Analysis on karyotype of *Achnatherum splendens* . J. Shiehzi Univ. 7, 3. (in Chinese).

[pbi13699-bib-0024] Haas, B.J. , Delcher, A.L. , Mount, S.M. , Wortman, J.R. , Smith, R.K. Jr , Hannick, L.I. , Maiti, R. *et al*. (2003) Improving the Arabidopsis genome annotation using maximal transcript alignment assemblies. Nucleic Acids Res. 31, 5654–5666. 14500829 10.1093/nar/gkg770 PMC206470

[pbi13699-bib-0025] Haas, B.J. , Salzberg, S.L. , Zhu, W. , Pertea, M. , Allen, J.E. , Orvis, J. , White, O. *et al*. (2008) Automated eukaryotic gene structure annotation using EvidenceModeler and the Program to Assemble Spliced Alignments. Genome Biol. 9, R7. 18190707 10.1186/gb-2008-9-1-r7 PMC2395244

[pbi13699-bib-0026] Haixia, X. , Xia, Z. , Shaoming, W. , Ping, Y. and Jinzhou, D. (2008) Genetic diversity of *Achnatherum splendens* . Agric. Sci. Technol. 36, 6223–6224.

[pbi13699-bib-0027] Huai, H. , Wei, W. and Zhang, Y. (2008) Characteristics of the *Achnatherum splendens* community along the Qinghai‐Tibet Railway, China. Front. Biol. China, 3, 477–483.

[pbi13699-bib-0028] Huang, L. , Feng, G. , Yan, H. , Zhang, Z. , Bushman, B.S. , Wang, J. , Bombarely, A. *et al*. (2020) Genome assembly provides insights into the genome evolution and flowering regulation of orchardgrass. Plant Biotechnol. J. 18, 373–388. 31276273 10.1111/pbi.13205 PMC6953241

[pbi13699-bib-0029] Hunter, S. , Apweiler, R. , Attwood, T.K. , Bairoch, A. , Bateman, A. , Binns, D. , Bork, P. *et al*. (2009) InterPro: the integrative protein signature database. Nucleic Acids Res., 37, D211–D215. 18940856 10.1093/nar/gkn785 PMC2686546

[pbi13699-bib-0030] Irfan, M. , Chen, Q. , Yue, Y. , Pang, R. , Lin, Q. , Zhao, X. and Chen, H. (2016) Co‐production of biochar, bio‐oil and syngas from halophyte grass (*Achnatherum splendens* L.) under three different pyrolysis temperatures. Bioresour. Technol. 211, 457–463. 27035478 10.1016/j.biortech.2016.03.077

[pbi13699-bib-0031] Jiang, Z.‐Y. , Li, X.‐Y. , Wu, H.‐W. , Zhang, S.‐Y. , Zhao, G.‐Q. and Wei, J.‐Q. (2017) Linking spatial distributions of the patchy grass *Achnatherum splendens* with dynamics of soil water and salt using electromagnetic induction. Catena, 149, 261–272.

[pbi13699-bib-0032] Jin, J. , Tian, F. , Yang, D.‐C. , Meng, Y.‐Q. , Kong, L. , Luo, J. and Gao, G. (2016) PlantTFDB 4.0: toward a central hub for transcription factors and regulatory interactions in plants. Nucleic Acids Res. 45, D1040–D1045. 27924042 10.1093/nar/gkw982 PMC5210657

[pbi13699-bib-0033] Jurka, J. , Kapitonov, V.V. , Pavlicek, A. , Klonowski, P. , Kohany, O. and Walichiewicz, J. (2005) Repbase update, a database of eukaryotic repetitive elements. Cytogenet. Genome Res. 110, 462–467. 16093699 10.1159/000084979

[pbi13699-bib-0034] Kanehisa, M. and Goto, S. (2000) KEGG: pomi encyclopedia of genes and genomes. Nucleic Acids Res. 28, 27–30. 10592173 10.1093/nar/28.1.27 PMC102409

[pbi13699-bib-0035] Koren, S. , Walenz, B.P. , Berlin, K. , Miller, J.R. , Bergman, N.H. and Phillippy, A.M. (2017) Canu: scalable and accurate long‐read assembly via adaptive k‐mer weighting and repeat separation. Genome Res. 27, 722–736. 28298431 10.1101/gr.215087.116 PMC5411767

[pbi13699-bib-0036] Korf, I. (2004) Gene finding in novel genomes. BMC Bioinformatics, 5, 59. 15144565 10.1186/1471-2105-5-59 PMC421630

[pbi13699-bib-0037] Kozomara, A. and Griffiths‐Jones, S. (2013) miRBase: annotating high confidence microRNAs using deep sequencing data. Nucleic Acids Res. 42, D68–D73. 24275495 10.1093/nar/gkt1181 PMC3965103

[pbi13699-bib-0038] Levin, H.L. and Moran, J.V. (2011) Dynamic interactions between transposable elements and their hosts. Nat. Rev. Genet. 12, 615–627. 21850042 10.1038/nrg3030 PMC3192332

[pbi13699-bib-0039] Li, H. and Durbin, R. (2009) Fast and accurate short read alignment with Burrows‐Wheeler transform. Bioinformatics, 25, 1754–1760. 19451168 10.1093/bioinformatics/btp324 PMC2705234

[pbi13699-bib-0040] Li, L. , Stoeckert, C.J. and Roos, D.S. (2003) OrthoMCL: identification of ortholog groups for eukaryotic genomes. Genome Res. 13, 2178–2189. 12952885 10.1101/gr.1224503 PMC403725

[pbi13699-bib-0041] Li, M.J. , Yang, Y.Z. , Xu, R.P. , Mu, W.J. , Li, Y. , Mao, X.X. , Zheng, Z.Y. *et al*. (2021) A chromosome‐level genome assembly for the Tertiary relict plant *Tetracentron sinense* Oliv. (Trochodendraceae). Mol. Ecol. Resour. 21, 1186–1199. 33486895 10.1111/1755-0998.13334

[pbi13699-bib-0042] Li, R. , Fan, W. , Tian, G. , Zhu, H. , He, L. , Cai, J. , Huang, Q. *et al*. (2010) The sequence and de novo assembly of the giant panda genome. Nature, 463, 311. 20010809 10.1038/nature08696 PMC3951497

[pbi13699-bib-0043] Liu, J. , Zhou, Y. , Luo, C. , Xiang, Y. and An, L. (2016) De novo transcriptome sequencing of desert herbaceous *Achnatherum splendens* (*Achnatherum*) seedlings and identification of salt tolerance genes. Genes, 7, 12. 27023614 10.3390/genes7040012 PMC4846842

[pbi13699-bib-0044] Lowe, T.M. and Eddy, S.R. (1997) tRNAscan‐SE: a program for improved detection of transfer RNA genes in genomic sequence. Nucleic Acids Res. 25, 955–964. 9023104 10.1093/nar/25.5.955 PMC146525

[pbi13699-bib-0045] Lukashin, A.V. and Borodovsky, M. (1998) GeneMark. Hmm: new solutions for gene finding. Nucleic Acids Res. 26, 1107–1115. 9461475 10.1093/nar/26.4.1107 PMC147337

[pbi13699-bib-0046] Lynch, M. and Conery, J.S. (2000) The evolutionary fate and consequences of duplicate genes. Science, 290, 1151–1155. 11073452 10.1126/science.290.5494.1151

[pbi13699-bib-0047] Ma, T. , Wang, J. , Zhou, G. , Yue, Z. , Hu, Q. , Chen, Y. , Liu, B. *et al*. (2013) Genomic insights into salt adaptation in a desert poplar. Nat. Commun. 4, 1–9. 10.1038/ncomms3797 24256998

[pbi13699-bib-0048] Mahajan, S. and Tuteja, N. (2005) Cold, salinity and drought stresses: an overview. Archi. Biochem. Biophys. 444, 139–158. 10.1016/j.abb.2005.10.018 16309626

[pbi13699-bib-0049] Marçais, G. and Kingsford, C. (2011) A fast, lock‐free approach for efficient parallel counting of occurrences of k‐mers. Bioinformatics, 27, 764–770. 21217122 10.1093/bioinformatics/btr011 PMC3051319

[pbi13699-bib-0050] Mihaela, P. , Pertea, G.M. , Antonescu, C.M. , Tsung‐Cheng, C. , Mendell, J.T. and Salzberg, S.L. (2015) StringTie enables improved reconstruction of a transcriptome from RNA‐seq reads. Nat. Biotechnol. 33, 290–295. 25690850 10.1038/nbt.3122 PMC4643835

[pbi13699-bib-0051] Møller, I.S. and Tester, M. (2007) Salinity tolerance of Arabidopsis: a good model for cereals? Trends Plant Sci. 12, 534–540. 18023242 10.1016/j.tplants.2007.09.009

[pbi13699-bib-0052] Morton, M.J. , Awlia, M. , Al‐Tamimi, N. , Saade, S. , Pailles, Y. , Negrão, S. and Tester, M. (2019) Salt stress under the scalpel–dissecting the genetics of salt tolerance. Plant J. 97, 148–163. 30548719 10.1111/tpj.14189 PMC6850516

[pbi13699-bib-0053] Murat, F. , Armero, A. , Pont, C. , Klopp, C. and Salse, J. (2017) Reconstructing the genome of the most recent common ancestor of flowering plants. Nat. Genet. 49, 490–496. 28288112 10.1038/ng.3813

[pbi13699-bib-0054] Murat, F. , Xu, J. , Tannier, E. , Abrouk, M. , Guilhot, N. , Pont, C. , Messing, J. *et al*. (2010) Ancestral grass karyotype reconstruction unravels new mechanisms of genome shuffling as a source of plant evolution. Genome Res. 20, 1545–1557. 20876790 10.1101/gr.109744.110 PMC2963818

[pbi13699-bib-0055] Nawrocki, E.P. and Eddy, S.R. (2013) Infernal 1.1: 100‐fold faster RNA homology searches. Bioinformatics, 29, 2933–2935. 24008419 10.1093/bioinformatics/btt509 PMC3810854

[pbi13699-bib-0056] Paterson, A.H. , Bowers, J.E. and Chapman, B. (2004) Ancient polyploidization predating divergence of the cereals, and its consequences for comparative genomics. Proc. Natl. Acad. Sci. USA, 101, 9903–9908. 15161969 10.1073/pnas.0307901101 PMC470771

[pbi13699-bib-0057] Paterson, A.H. , Bowers, J.E. , Bruggmann, R. , Dubchak, I. , Grimwood, J. , Gundlach, H. , Haberer, G. *et al*. (2009) The Sorghum bicolor genome and the diversification of grasses. Nature, 457, 551–556. 19189423 10.1038/nature07723

[pbi13699-bib-0058] Porebski, S. , Bailey, L.G. and Baum, B.R. (1997) Modification of a CTAB DNA extraction protocol for plants containing high polysaccharide and polyphenol components. Plant Mol. Biol. Rep. 15, 8–15.

[pbi13699-bib-0059] Punta, M. , Coggill, P.C. , Eberhardt, R.Y. , Mistry, J. , Tate, J. , Boursnell, C. , Pang, N. *et al*. (2011) The Pfam protein families database. Nucleic Acids Res. 40, D290–D301. 22127870 10.1093/nar/gkr1065 PMC3245129

[pbi13699-bib-0060] Qadir, M. , Quillerou, E. , Nangia, V. , Murtaza, G. , Singh, M. , Thomas, R.J. , Drechsel, P. *et al*. (2014) Economics of salt‐induced land degradation and restoration. Nat. Resour. Forum, 38, 282–295.

[pbi13699-bib-0061] Roach, M.J. , Schmidt, S.A. and Borneman, A.R. (2018) Purge Haplotigs: allelic contig reassignment for third‐gen diploid genome assemblies. BMC Bioinformatics, 19, 460. 30497373 10.1186/s12859-018-2485-7 PMC6267036

[pbi13699-bib-0062] Salmanminkov, A. , Sabath, N. and Mayrose, I. (2016) Whole‐genome duplication as a key factor in crop domestication. Nat. Plants, 2, 16115. 27479829 10.1038/nplants.2016.115

[pbi13699-bib-0063] Servant, N. , Varoquaux, N. , Lajoie, B.R. , Viara, E. , Chen, C.J. , Vert, J.P. , Heard, E. *et al*. (2015) HiC‐Pro: an optimized and flexible pipeline for Hi‐C data processing. Genome Biol., 16, 259. 26619908 10.1186/s13059-015-0831-x PMC4665391

[pbi13699-bib-0064] Shabala, S. and Cuin, T.A. (2008) Potassium transport and plant salt tolerance. Physiol. Plant, 133, 651–669. 18724408 10.1111/j.1399-3054.2007.01008.x

[pbi13699-bib-0065] Soltis, D.E. , Visger, C.J. and Soltis, P.S. (2014) The polyploidy revolution then… and now: Stebbins revisited. Am. J. Bot. 101, 1057–1078. 25049267 10.3732/ajb.1400178

[pbi13699-bib-0066] Stamatakis, A. (2014) RaxML version 8: a tool for phylogenetic analysis and post‐analysis of large phylogenies. Bioinformatics, 30, 1312–1313. 24451623 10.1093/bioinformatics/btu033 PMC3998144

[pbi13699-bib-0067] Stanke, M. , Diekhans, M. , Baertsch, R. and Haussler, D. (2008) Using native and syntenically mapped cDNA alignments to improve de novo gene finding. Bioinformatics, 24, 637–644. 18218656 10.1093/bioinformatics/btn013

[pbi13699-bib-0068] Sun, P.C. , Jiao, B.B. , Yang, Y.Z. , Shan, L.X. , Li, T. , Li, X.N. , Xi, Z.X. *et al*. (2021) WGDI: a user‐friendly toolkit for evolutionary analyses of whole‐genome duplications and ancestral karyotypes. BioRxiv, 10.1101/2021.04.29.441969 36307977

[pbi13699-bib-0069] Suyama, M. , Torrents, D. and Bork, P. (2006) PAL2NAL: robust conversion of protein sequence alignments into the corresponding codon alignments. Nucleic Acids Res. 34, W609–W612. 16845082 10.1093/nar/gkl315 PMC1538804

[pbi13699-bib-0070] Tang, H. , Bowers, J.E. , Wang, X. , Ming, R. , Alam, M. and Paterson, A.H. (2008) Synteny and collinearity in plant genomes. Science, 320, 486–488. 18436778 10.1126/science.1153917

[pbi13699-bib-0071] Tarailo‐Graovac, M. and Chen, N. (2009) Using RepeatMasker to identify repetitive elements in genomic sequences. Curr. Protoc. Bioinformatics, 25, 4.10.11–14.10.14. 10.1002/0471250953.bi0410s25 19274634

[pbi13699-bib-0072] Varshney, R.K. , Shi, C. , Thudi, M. , Mariac, C. , Wallace, J. , Qi, P. , Zhang, H. *et al*. (2017) Pearl millet genome sequence provides a resource to improve agronomic traits in arid environments. Nat. Biotechnol. 35, 969–976. 28922347 10.1038/nbt.3943 PMC6871012

[pbi13699-bib-0073] Vicient, C.M. , Suoniemi, A. , Anamthawat‐Jónsson, K. , Tanskanen, J. , Beharav, A. , Nevo, E. and Schulman, A.H. (1999) Retrotransposon BARE‐1 and its role in genome evolution in the genus Hordeum. Plant Cell, 11, 1769–1784. 10488242 10.1105/tpc.11.9.1769 PMC144304

[pbi13699-bib-0074] Walker, B.J. , Abeel, T. , Shea, T. , Priest, M. , Abouelliel, A. , Sakthikumar, S. , Cuomo, C.A. *et al*. (2014) Pilon: an integrated tool for comprehensive microbial variant detection and genome assembly improvement. PloS One, 9, e112963. 25409509 10.1371/journal.pone.0112963 PMC4237348

[pbi13699-bib-0075] Wang, D. , Zhang, Y. , Zhang, Z. , Zhu, J. and Yu, J. (2010) KaKs_Calculator 2.0: a toolkit incorporating gamma‐series methods and sliding window strategies. Genomics Proteomics Bioinformatics, 8, 77–80. 20451164 10.1016/S1672-0229(10)60008-3 PMC5054116

[pbi13699-bib-0076] Wang, J. , Sun, P. , Li, Y. , Liu, Y. , Yu, J. , Ma, X. , Sun, S. *et al*. (2017) Hierarchically aligning 10 legume genomes establishes a family‐level genomics platform. Plant Physiol. 174, 284–300. 28325848 10.1104/pp.16.01981 PMC5411148

[pbi13699-bib-0077] Wang, J.‐P. , Yu, J.‐G. , Li, J. , Sun, P.‐C. , Wang, L.I. , Yuan, J.‐Q. , Meng, F.‐B. *et* *al*. (2018) Two likely auto‐tetraploidization events shaped kiwifruit genome and contributed to establishment of the Actinidiaceae family. iScience, 7, 230–240. 30267683 10.1016/j.isci.2018.08.003 PMC6161637

[pbi13699-bib-0078] Wang, M. , Tong, S. , Ma, T. , Xi, Z. and Liu, J.Q. (2021) Chromosome‐level genome assembly of Sichuan pepper provides insights into pomixes, drought tolerance, and alkaloid biosynthesis. Mol. Ecol. Resour. 21, 2533–2545. 34145765 10.1111/1755-0998.13449

[pbi13699-bib-0079] Wang, X. , Shi, X. , Hao, B. , Ge, S. and Luo, J. (2005) Duplication and DNA segmental loss in the rice genome: implications for diploidization. New Phytol. 165, 937–946. 15720704 10.1111/j.1469-8137.2004.01293.x

[pbi13699-bib-0080] Wang, X. , Shi, X. , Li, Z. , Zhu, Q. , Kong, L. , Tang, W.Y. , Ge, S. *et al*. (2006) Statistical inference of chromosomal homology based on gene colinearity and applications to Arabidopsis and rice. BMC Bioinformatics, 7, 447. 17038171 10.1186/1471-2105-7-447 PMC1626491

[pbi13699-bib-0081] Wang, X. , Tang, H. , Bowers, J.E. and Paterson, A.H. (2009) Comparative inference of illegitimate recombination between rice and sorghum duplicated genes produced by polyploidization. Genome Res. 19, 1026–1032. 19372385 10.1101/gr.087288.108 PMC2694483

[pbi13699-bib-0082] Wang, X. , Wang, J. , Jin, D. , Guo, H. , Lee, T. , Liu, T. and Paterson, A.H. (2015) Genome alignment spanning major poaceae lineages reveals heterogeneous evolutionary rates and alters inferred dates for key evolutionary events. Mol. Plant, 8, 885–898. 25896453 10.1016/j.molp.2015.04.004

[pbi13699-bib-0083] Wang, Y. , Tang, H. , DeBarry, J.D. , Tan, X. , Li, J. , Wang, X. , Lee, T.‐H. *et* *al*. (2012) MCScanX: a toolkit for detection and evolutionary analysis of gene synteny and collinearity. Nucleic Acids Res. 40, e49. 22217600 10.1093/nar/gkr1293 PMC3326336

[pbi13699-bib-0084] Wendel, J.F. (2000) Genome evolution in polyploids. Plant Mol. Biol. 42, 225–249. 10688139

[pbi13699-bib-0085] Wu, H.‐J. , Zhang, Z. , Wang, J.‐Y. , Oh, D.‐H. , Dassanayake, M. , Liu, B. , Huang, Q. *et al*. (2012) Insights into salt tolerance from the genome of *Thellungiella salsuginea* . Proc. Natl. Acad. Sci. USA, 109, 12219–12224. 22778405 10.1073/pnas.1209954109 PMC3409768

[pbi13699-bib-0086] Wu, T.D. and Watanabe, C.K. (2005) GMAP: a genomic mapping and alignment program for mRNA and EST sequences. Bioinformatics, 21, 1859–1875. 15728110 10.1093/bioinformatics/bti310

[pbi13699-bib-0087] Xu, Z. and Wang, H. (2007) LTR_FINDER: an efficient tool for the prediction of full‐length LTR retrotransposons. Nucleic Acids Res. 35, W265–W268. 17485477 10.1093/nar/gkm286 PMC1933203

[pbi13699-bib-0088] Yamaguchi, T. and Blumwald, E. (2005) Developing salt‐tolerant crop plants: challenges and opportunities. Trends Plant Sci. 10, 615–620. 16280254 10.1016/j.tplants.2005.10.002

[pbi13699-bib-0089] Yang, Z. (2007) PAML 4: phylogenetic analysis by maximum likelihood. Mol. Biol. Evol, 24, 1586–1591. 17483113 10.1093/molbev/msm088

[pbi13699-bib-0090] You, L. , Liu, B. and Bussmann, R.W. (2020) Achnatherum splendens (Trin.) Nevski P oaceae. Ethnobotany of the Mountain Regions of Central Asia and Altai, 1–3.

[pbi13699-bib-0091] Zdobnov, E.M. and Apweiler, R. (2001) InterProScan–an integration platform for the signature‐recognition methods in InterPro. Bioinformatics, 17, 847–848. 11590104 10.1093/bioinformatics/17.9.847

[pbi13699-bib-0092] Zhang, Y. , Liang, C. , Wei, W. , Wang, L. , Peng, J. , Yan, J. and Jia, C. (2010) Soil salinity and Achnatherum splendens distribution. Chin. J. Ecol. 29, 2438–2443.

[pbi13699-bib-0093] Zhu, J. (2001) Plant salt tolerance. Trends Plant Sci. 6, 66–71. 11173290 10.1016/s1360-1385(00)01838-0

[pbi13699-bib-0094] Zhu, J. , Fu, X. , Koo, Y.D. , Zhu, J.‐K. , Jenney, F.E. , Adams, M.W. , Zhu, Y. *et al*. (2007) An enhancer mutant of Arabidopsis salt overly sensitive 3 mediates both ion homeostasis and the oxidative stress response. Mol. Cell. Biol. 27, 5214–5224. 17485445 10.1128/MCB.01989-06 PMC1951954

