# Supplementary material for: The genome sequence provides insights into salt tolerance of Achnatherum splendens (Gramineae), a constructive species of alkaline grassland
Source: Plant Biotechnol J. 2021 Sep 15;20(1):116–28. doi: 10.1111/pbi.13699 (PMC8710827; doi:10.1111/pbi.13699)
Supplement: Supplementary file 1 — Figure S1 K‐mer analysis of the A. splendens genome by using K‐mer = 17. Figure S2 Hi‐C‐assisted assembly of A. splendens pseudochromosomes. Figure S3 Comparison of (a) CDS length, (b) mRNA length, (c) exon length and (d) intron length between A. splendens and other six related species. Figure S4 Gene ontology (GO) enrichment of the expanded gene families in A. splendens. Figure S5 Overview of dotplots within the A. splendens genome paralogous genes. Figure S6 (a) Overview of dotplots between A. splendens and B. distachyon genome homologous genes. (b) Example of homologous gene dotplots between A. splendens and B. distachyon. Chromosome numbers and regions (in Mbp) were shown. Best hit (orthologous) genes are red dots, secondary hits (outparalogous) are blue dots and the others are shown in grey. Highlights show the best matched chromosomal regions. Arrows show complement correspondence produced by chromosome breakages during evolution. Figure S7 Hierarchical cluster analysis of gene expression in root and shoot tissues. Figure S8 Gene ontology (GO) enrichment of the 402 pairs of paralogous genes in A. splendens. Table S1 Estimation of the A. splendens genome size based on 17‐mer statistics. Table S2 Summary of DNA sequencing data. Table S3 Assembly statistics based on Hi‐C data. Table S4 Genome assembly completeness evaluation by BUSCO. Table S5 Summary of PacBio full‐length cDNA sequencing. Table S6 The length distribution of full‐length (FL) transcripts. Table S7 Classification of interspersed repeats in the assembled A. splendens genome. Table S8 LTR subclass ratio in A. splendens and other five Gramineae genomes. Table S9 Summary of predicted protein‐coding gene annotations and their supporting evidence types. Table S10 Functional annotation of predicted genes in the A. splendens genome. Table S11 Summary statistics of non‐coding RNAs in the A. splendens genome. Table S12 Summary of gene family clustering. Table S13 Gene ontology (GO) enrichment analysis of the [file PBI-20-116-s001.doc]

**Supplementary Information**

**Supplementary Figures**


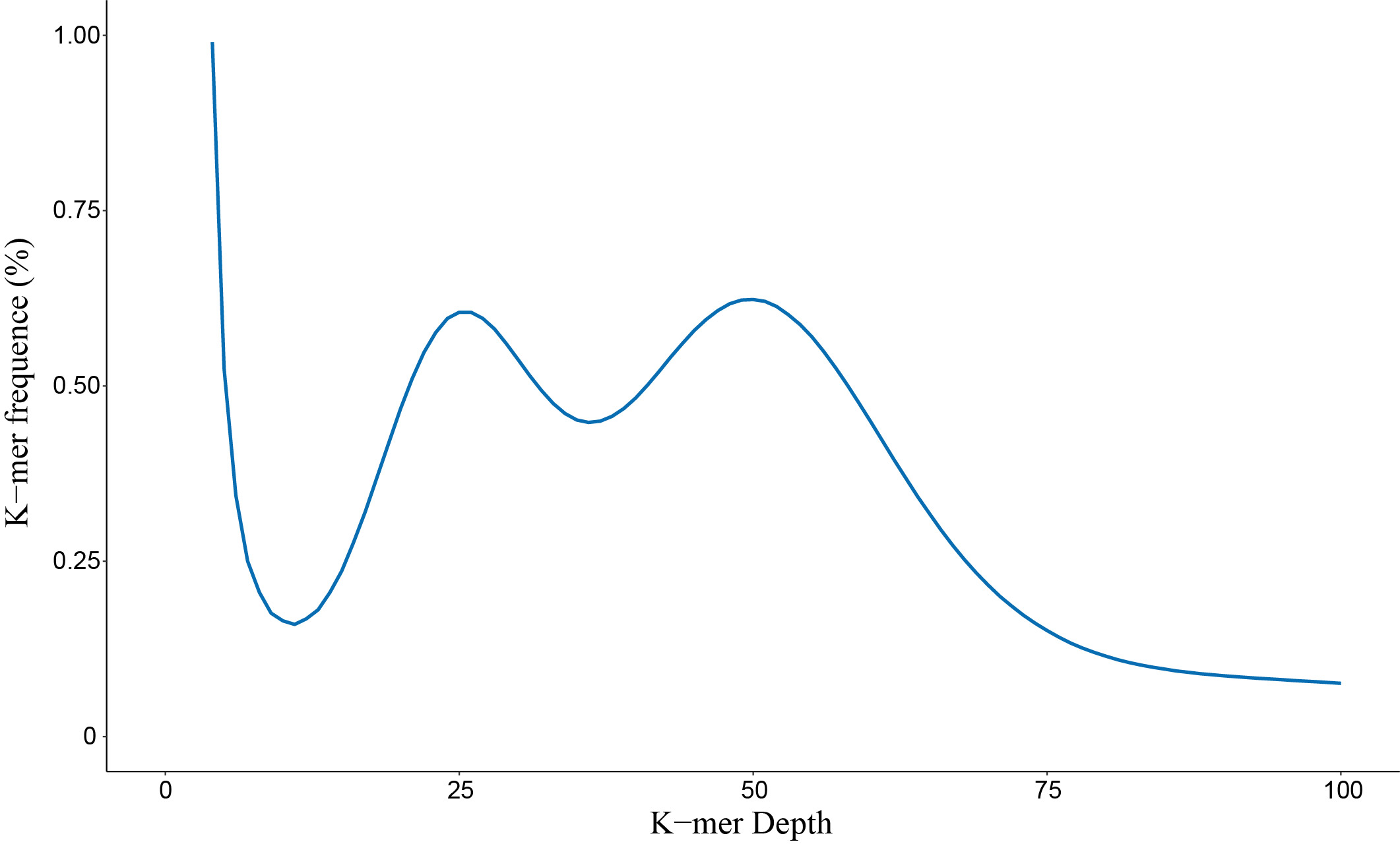


**Figure S1** K-mer analysis of the *A. splendens* genome by using K-mer = 17.


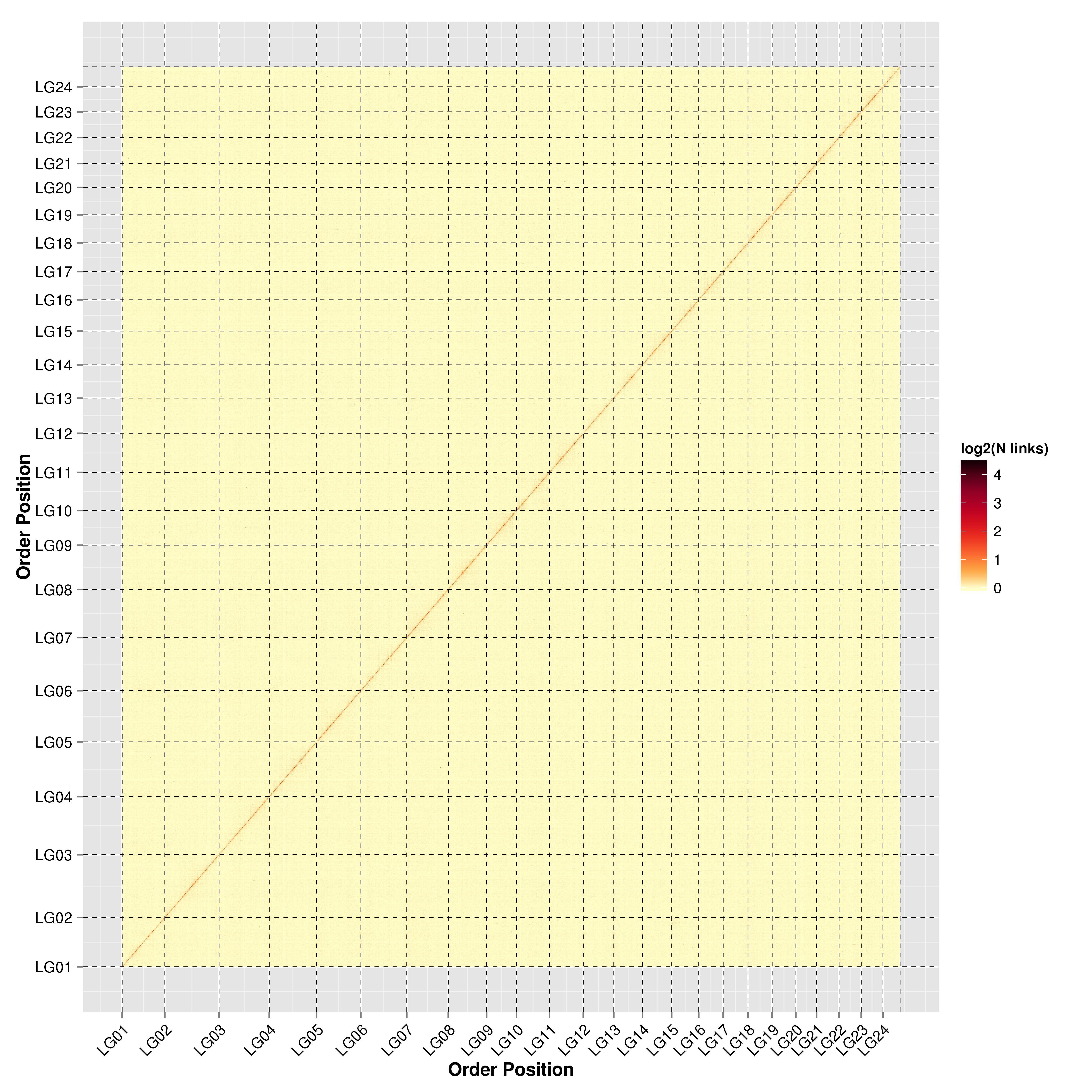
 **Figure S2** Hi-C assisted assembly of *A. splendens* pseudochromosomes. Heatmap showing Hi-C interactions under a resolution of 200 kb, and the antidiagonal pattern for the intrachromosomal interactions may reflect the Rabl configuration of chromatins.

**
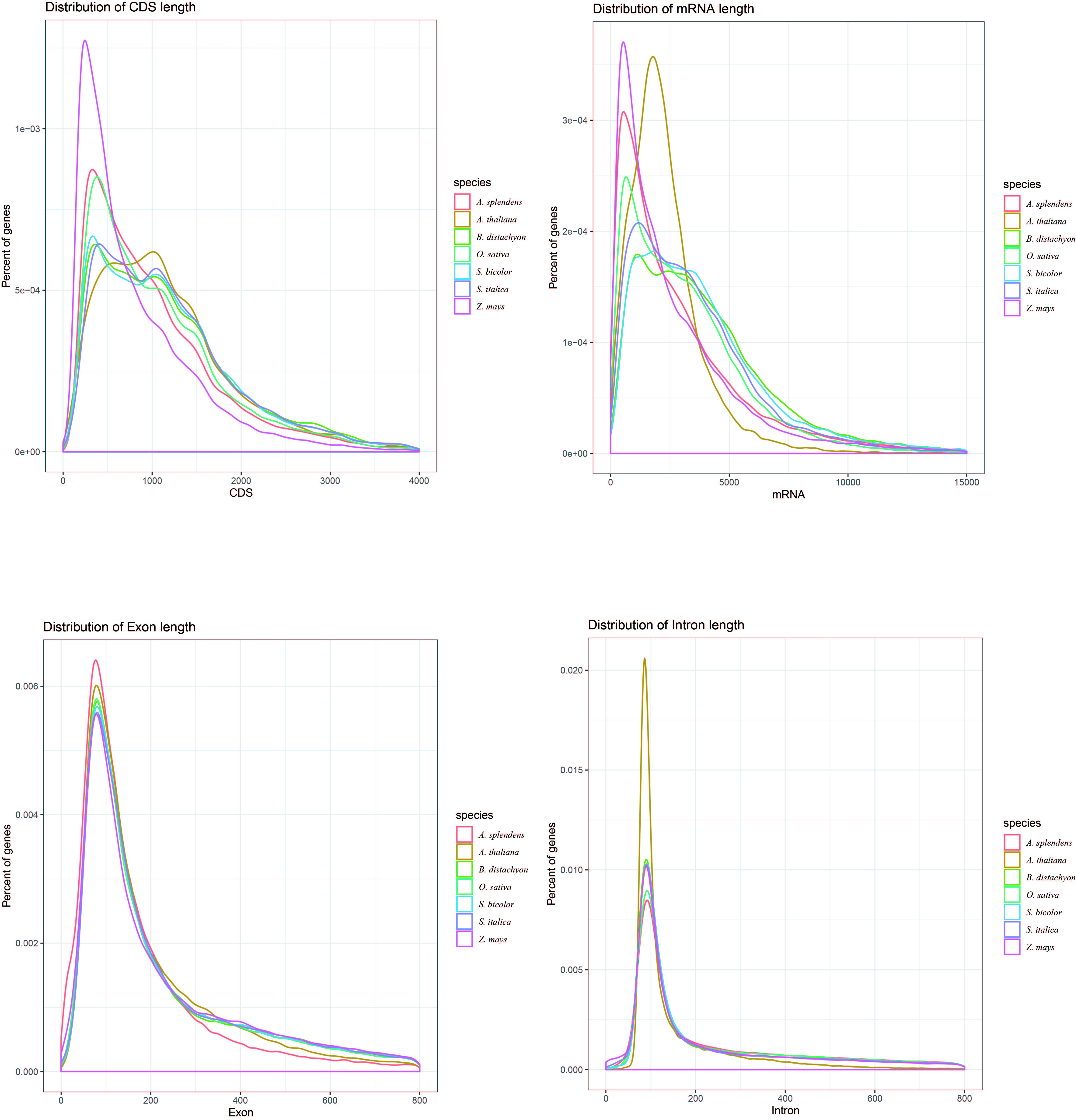
**

**Figure S3** Comparison of (a) CDS length, (b) mRNA length, (c) Exon length, (d) Intron length between *A. splendens* and other 6 related species.

**
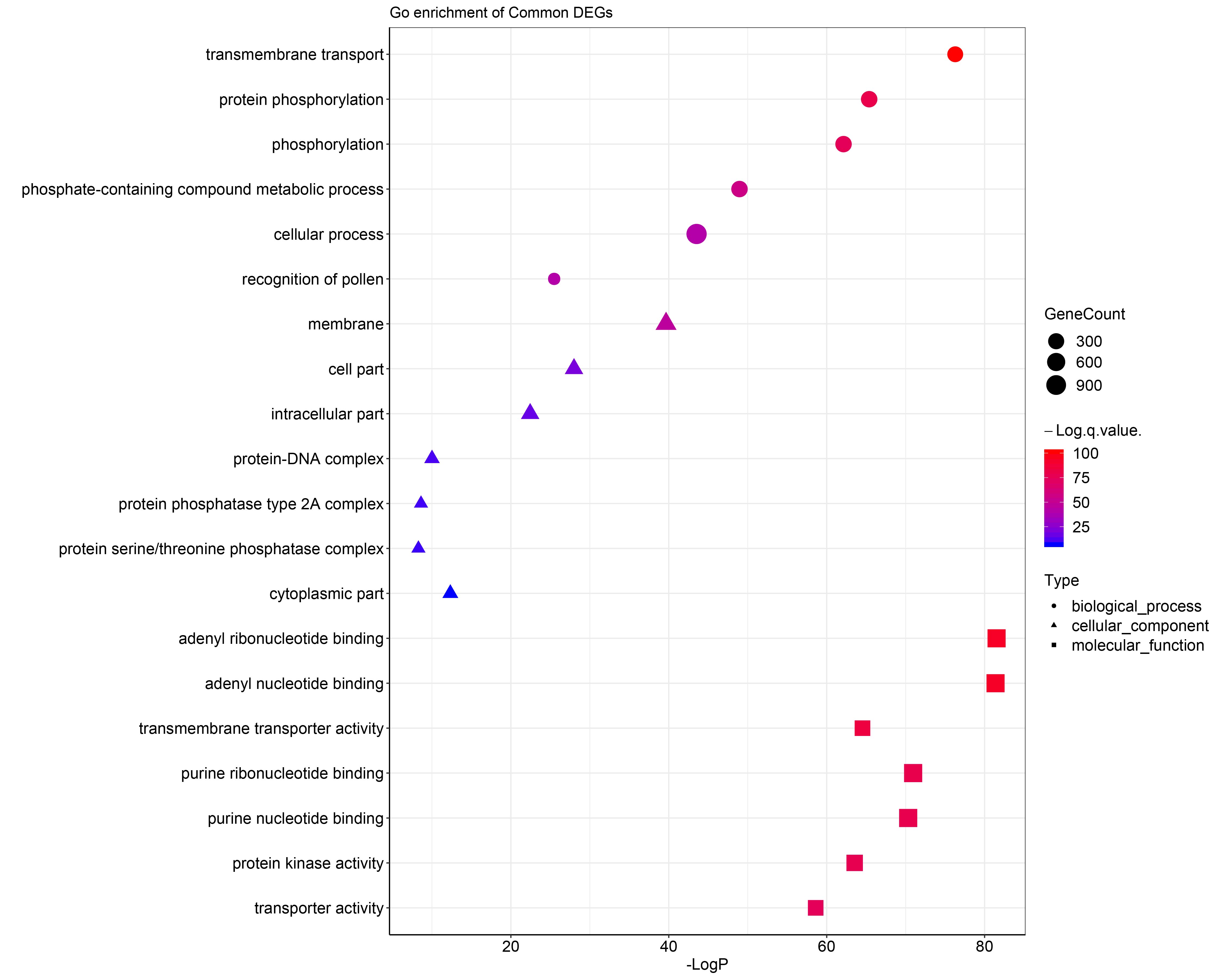
**

**Figure S4** Gene Ontology (GO) enrichment of the expanded gene families in *A. splendens*. Only the top 20 significant terms are shown. The x-axis (gene ratio) represents the ratio of gene numbers annotated to one GO term and annotated to all GO terms. The colour and size of the dots represent the range of the *P*-value and the number of bract-biased genes mapped to the indicated GO terms, respectively. Circles, triangles and squares represent the three types of GO terms: biological process, cellular component and molecular function, respectively.

**
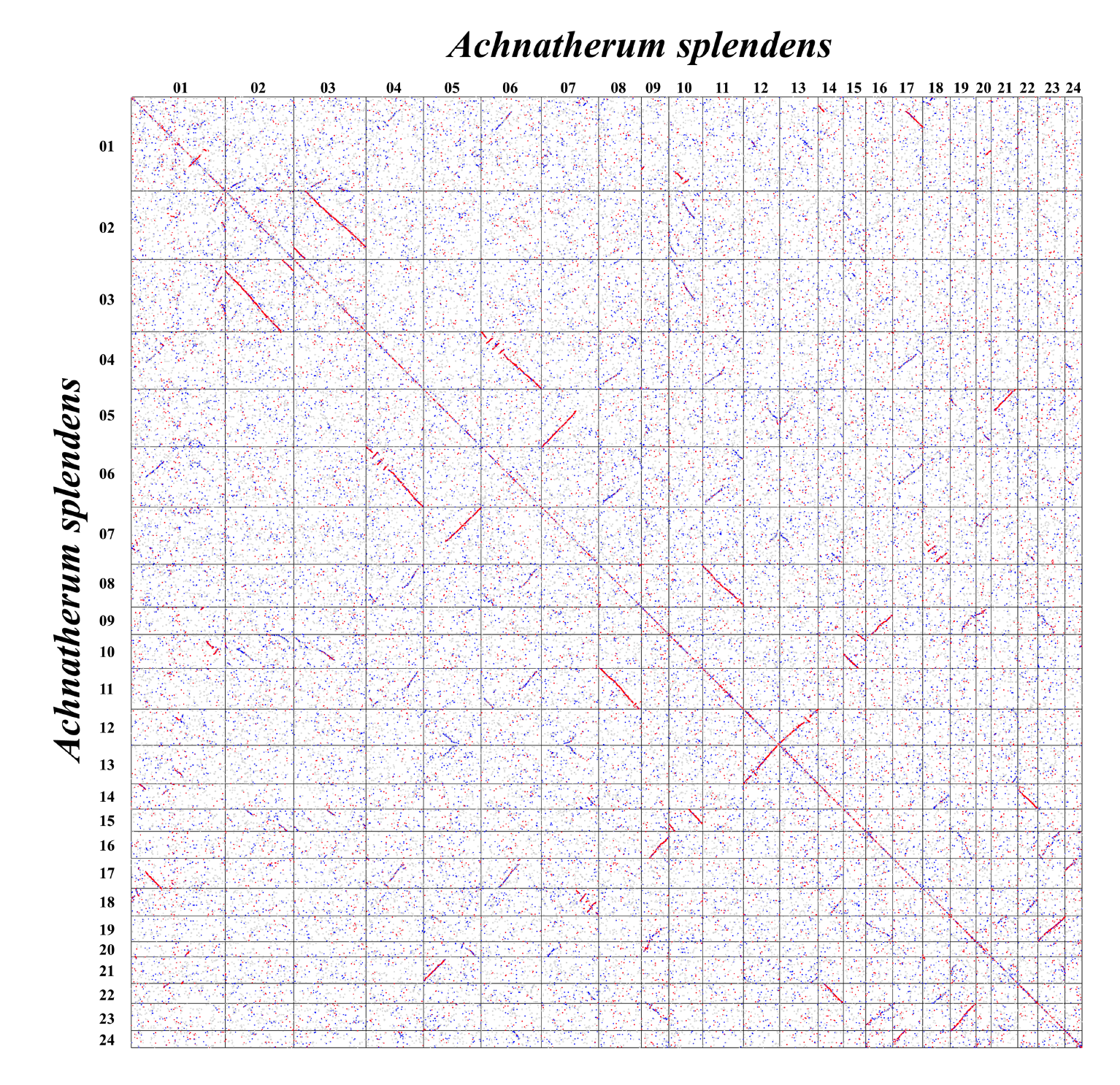
**

**Figure S5** Overview of dotplots within the *A. splendens* genome paralogous genes.

**Figure S6** (a) Overview of dotplots between *A. splendens* and *B. distachyon* genome homologous genes. (b) Example of homologous gene dotplots between *A. splendens* and *B. distachyon*. Chromosome numbers and regions (in Mbp) were shown. Best-hit (orthologous) genes are red dots, secondary hits (outparalogous) are blue dots, and the others are shown in gray. Highlights show the best matched chromosomal regions. Arrows show complement correspondence produced by chromosome breakages during evolution.

**Figure S7** Hierarchical cluster analysis of gene expression in root and shoot tissues.

**Figure S8** Gene Ontology (GO) enrichment of the 402 pairs of paralogous genes in *A. splendens*. The x-axis (Gene_ratio) represents the ratio of gene numbers annotated to one GO term and annotated to all GO terms. The colour and size of the dots represent the range of the *P*-value and the number of bract-biased genes mapped to the indicated GO terms, respectively. Circles, triangles and squares represent the three types of GO terms: biological process, cellular component and molecular function, respectively.

# Supplementary Tables

**Table S1** Estimation of the *A. splendens* genome size based on 17-mer statistics.

| **K-mer** | **K-mer num** | **K-mer Depth** | **Genome Size** | **Used Base** | **Repeat** | **Hete** |
| --- | --- | --- | --- | --- | --- | --- |
| 17 | 57,879,333,379 | 52 | 1,113,064,103 | 67,279,018,040 | 0.62 | 0.013 |

**Table S2** Summary of DNA sequencing data.

| **Read type** | **Total raw reads** | **Insert size (bp)** | **Read length / Mean subread length (N50)** | **Sequence coverage**^1^**(X)** |
| --- | --- | --- | --- | --- |
| **Illumina pair-end reads** | 142,824,454,500 | 270 | 2 x 150 bp | 128 |
| **Hi-C pair-end reads** | 123,418,456,296 | 300 | 2 x 150 bp | 111 |
| **PacBio subreads** | 83,814,074,321 | - | 10.08 kb  (14.59 kb) | 75 |

^1^Depth was calculated under the estimate of a genome size of 1.113 Gb.

**Table S3** Assembly statistics based on Hi-C data.

| Group | Cluster Num | Cluster Len (bp) | Order Num | Order Len (bp) |
| --- | --- | --- | --- | --- |
| LG01 | 126 | 60,274,297 | 87 | 56,522,540 |
| LG02 | 177 | 75,437,823 | 132 | 71,846,896 |
| LG03 | 184 | 70,320,975 | 135 | 66,423,842 |
| LG04 | 190 | 69,796,262 | 124 | 62,521,810 |
| LG05 | 160 | 61,967,733 | 116 | 58,573,686 |
| LG06 | 154 | 64,231,125 | 111 | 60,746,629 |
| LG07 | 121 | 58,461,814 | 83 | 54,951,602 |
| LG08 | 137 | 53,736,379 | 96 | 50,754,284 |
| LG09 | 138 | 43,721,307 | 89 | 39,630,148 |
| LG10 | 136 | 47,210,395 | 89 | 43,593,887 |
| LG11 | 125 | 47,443,727 | 91 | 44,789,340 |
| LG12 | 133 | 44,922,628 | 80 | 40,289,208 |
| LG13 | 115 | 40,769,711 | 79 | 38,086,986 |
| LG14 | 122 | 41,713,026 | 86 | 38,726,960 |
| LG15 | 112 | 37,949,748 | 84 | 35,783,571 |
| LG16 | 143 | 37,647,562 | 88 | 32,317,294 |
| LG17 | 119 | 37,041,946 | 81 | 32,830,253 |
| LG18 | 103 | 34,597,425 | 71 | 32,059,127 |
| LG19 | 110 | 34,211,204 | 76 | 31,412,567 |
| LG20 | 116 | 32,034,982 | 71 | 27,435,083 |
| LG21 | 89 | 31,761,443 | 62 | 29,753,175 |
| LG22 | 105 | 31,640,612 | 71 | 29,333,239 |
| LG23 | 78 | 30,585,488 | 52 | 28,679,215 |
| LG24 | 111 | 28,571,766 | 64 | 22,831,543 |
| Total (Ratio %) | 3,104  (87.31) | 1,116,049,378 (94.85) | 2118  (68.23) | 1,029,892,885  (92.28) |

**Table S4** Genome assembly completeness evaluation by BUSCO.

| C:97.3%, F:0.6%, M:2.1%, n:1440 | |
| --- | --- |
| Type | Number |
| Complete BUSCOs (C) | 1,401 |
| Complete and single-copy BUSCOs (S) | 686 |
| Complete and duplicated BUSCOs (D) | 715 |
| Fragmented BUSCOs (F) | 8 |
| Missing BUSCOs (M) | 31 |
| Total BUSCO groups searched | 1,440 |

**Table S5** Summary of PacBio full-length cDNA sequencing.

| **Tissue** | **Read type** | **Cell Number** | **Reads of Insert** | **Read Bases of Insert (bp)** | **Mean Read Length of Insert (bp)** | **Mean Read Quality of Insert** |
| --- | --- | --- | --- | --- | --- | --- |
| Mixed samples | PacBio full-length cDNA | 1 | 192,705 | 564,975,334 | 2,932 | 0.94 |
|  |  | 1 | 283,048 | 806,226,919 | 2,848 | 0.94 |

Quality shows how confident the reads are. Usually shorter reads have higher quality.

**Table S6** The length distribution of full-length (FL) transcripts.

| **FL transcripts length interval** | **200-500bp** | **500-1000bp** | **1-2kb** | **>2kb** | **Total** | **Alignment Rate** |
| --- | --- | --- | --- | --- | --- | --- |
| **Number of FL transcripts** | 2,342 | 9,400 | 23,842 | 38,962 | 74,546 | 73,770 (98.96%) |

**Table S7** Classification of interspersed repeats in the assembled *A. splendens* genome.

| **Class** | **Subfamily** | **Length (bp)** | **Percent (%)** |
| --- | --- | --- | --- |
| **Class I: Retrotransposon** | Total LTR | 477,703,887 | 40.83 |
|  | LTR/Copia | 146,945,289 | 12.56 |
|  | LTR/Gypsy | 322,955,183 | 27.60 |
|  | Other LTR | 10,705,593 | 0.92 |
|  | SINEs | 1,290,536 | 0.11 |
|  | Toatal LINEs | 41,706,710 | 3.56 |
|  | LINE/L1 | 39,898,023 | 3.41 |
|  | LINE/L2 | 1,020,022 | 0.09 |
|  | Other LINE | 803,042 | 0.07 |
| **Class II: DNA Transposon** | Total DNA | 147,800,064 | 12.63 |
|  | CMC-EnSpm | 89,958,833 | 7.69 |
|  | hAT-Ac | 6,482,824 | 0.55 |
|  | hAT-Charlie | 1,865,433 | 0.16 |
|  | PIF-Harbinger | 13,012,310 | 1.11 |
|  | TcMar-Stowaway | 4,103,483 | 0.35 |
|  | MULE-MuDR | 27,114,969 | 2.32 |
|  | Other DNA | 6,167,161 | 0.53 |
| **Unclassified** |  | 79,440,739 | 6.79 |
| **Low_complexity** |  | 1,167,059 | 0.10 |
| **Simple_repeat** |  | 41,448,547 | 3.54 |
| **Satellite** |  | 493,859 | 0.04 |
| **Total content** |  | 731,956,730 | 62.56 |

**Table S8 LTR subclass ratio in *A. splendens* and other five Gramineae genomes.**

| LTR | *A. splendens* | *A. tauschii* | *T. urartu* | *H. vulgare* | *B. distachyon* | *O. sativa* |
| --- | --- | --- | --- | --- | --- | --- |
| Copia | 12.47 | 16.83% | 16.45% | 21.60% | 4.44% | 3.45% |
| Gypsy | 27.40 | 38.40% | 45.84% | 46.02% | 14.22% | 17.45% |
| Gypsy/ Copia | 2.23 | 2.28 | 2.79 | 2.13 | 3.2 | 5.06 |

**Table S9 Summary of predicted protein-coding gene annotations and their supporting evidence types.**

| Gene set | | Total Genes Predicted | Average Gene Length (bp) | Average CDS Length (bp) | Average Exons per Gene | Average Exon Length (bp) | Average Intron Length (bp) |
| --- | --- | --- | --- | --- | --- | --- | --- |
| *De novo* | AUGUSTUS | 48,276 | 3,371.94 | 1,184.76 | 5.45 | 217.30 | 490.25 |
|  | GENEMARK | 111,617 | 1,959.50 | 618.78 | 4.32 | 143.28 | 404.00 |
|  | GENEID | 53,048 | 6,244.07 | 595.02 | 3.31 | 179.57 | 2,441.65 |
|  | SNAP | 92,992 | 3,978.60 | 716.29 | 4.01 | 178.66 | 1,084.06 |
| Homolog | *A. thaliana* | 35,898 | 2,529.48 | 972.49 | 4.22 | 229.95 | 500.80 |
|  | *O. sativa* | 50,536 | 2,335.62 | 928.39 | 3.89 | 238.60 | 502.79 |
|  | *B. distachyon* | 54,597 | 2,330.98 | 926.89 | 3.89 | 229.95 | 505.88 |
|  | *S. italica* | 51,477 | 2,400.33 | 950.47 | 4.01 | 236.82 | 497.65 |
|  | *S. bicolor* | 50,309 | 2,408.26 | 956.26 | 4.02 | 237.72 | 495.53 |
|  | *Z. mays* | 58,158 | 2,075.70 | 854.72 | 3.53 | 242.39 | 497.55 |
|  | *H. vulgare* | 53,814 | 2,122.99 | 872.61 | 3.62 | 241.09 | 493.28 |
| RNA_seq |  | 17,429 | 4,803.70 | 1,245.83 | 5.33 | 233.71 | 470.18 |
| EVM |  | 58,386 | 3,234.21 | 1,046.26 | 4.82 | 217.17 | 573.12 |
| Final Set |  | 57,374 | 3,250.81 | 1,052.66 | 4.85 | 217.04 | 569.75 |

**Table S10** Functional annotation of predicted genes in the *A. splendens* genome.

| Type | | Number | Percent of gene number (%) |
| --- | --- | --- | --- |
| Annotation | InterPro | 41,689 | 72.66 |
|  | GO | 41,331 | 72.04 |
|  | KEGG | 11,048 | 19.26 |
|  | Swissprot | 39,023 | 68.02 |
| Total | Annotated | 54,426 | 94.86 |
|  | Unannotated | 3,194 | 5.57 |
|  | Total gene | 57,374 | 100 |

**Table S11** Summary statistics of non-coding RNAs in the *A. splendens* genome.

| Type | Number | Average_length (bp) | Total_length (bp) | % of genome |
| --- | --- | --- | --- | --- |
| **miRNA** | 644 | 153.89 | 99,105 | 0.003419 |
| **tRNA** | 898 | 73.72 | 66,203 | 0.005617 |
| **rRNA** | 885 | 134.69 | 119,205 | 0.004113 |
| **snRNA** | 447 | 121.37 | 54,252 | 0.001872 |

**Table S12 Summary of gene family clustering.**

| Species | Total genes | Genes in families | Unclustered genes | Families | Unique families | Genes per family | Maximum gene family size |
| --- | --- | --- | --- | --- | --- | --- | --- |
| *A. tauschii* | 38,735 | 28,259 | 10,476 | 17,590 | 734 | 1.61 | 148 |
| *A. thaliana* | 27,416 | 22,885 | 4,531 | 11,754 | 1,273 | 1.95 | 142 |
| *B. distachyon* | 34,310 | 26,395 | 7,915 | 17,931 | 413 | 1.47 | 96 |
| *H. vulgare* | 39,734 | 31,245 | 8,489 | 18,011 | 1,270 | 1.73 | 107 |
| *A. splendens* | 57,682 | 38,318 | 19,364 | 17,916 | 1,116 | 2.14 | 155 |
| *O. sativa* | 39,049 | 27,851 | 11,198 | 18,168 | 934 | 1.53 | 100 |
| *P. edulis* | 50,936 | 38,682 | 12,254 | 17,903 | 1,295 | 2.16 | 106 |
| *S. bicolor* | 34,129 | 27,577 | 6,552 | 18,799 | 453 | 1.47 | 212 |
| *S. italica* | 34,584 | 28,654 | 5,930 | 18,804 | 395 | 1.52 | 147 |
| *Z. mays* | 63,480 | 40,348 | 23,132 | 20,730 | 3,772 | 1.95 | 116 |
| Total | 420,055 | 310,214 | 109,841 | 177,606 | - | 17.53 | - |

**Table S13** Gene ontology (GO) enrichment analysis of the unique families in *A. splendens*.

| **GO Terms** | **Type** | **P-value** | **Function** |
| --- | --- | --- | --- |
| GO:0006979 | biological_process | 1.42E-05 | response to oxidative stress |
| GO:0016684 | molecular_function | 3.75E-05 | oxidoreductase activity, acting on peroxide as acceptor |
| GO:0050794 | biological_process | 3.85E-05 | regulation of cellular process |
| GO:0009987 | biological_process | 4.12E-05 | cellular process |
| GO:0007166 | biological_process | 1.25E-04 | cell surface receptor signaling pathway |
| GO:0016423 | molecular_function | 2.25E-04 | tRNA (guanine) methyltransferase activity |
| GO:0005524 | molecular_function | 2.40E-04 | ATP binding |
| GO:0003824 | molecular_function | 2.82E-04 | catalytic activity |
| GO:0030554 | molecular_function | 4.60E-04 | adenyl nucleotide binding |
| GO:0032559 | molecular_function | 4.96E-04 | adenyl ribonucleotide binding |
| GO:0050789 | biological_process | 4.99E-04 | regulation of biological process |
| GO:2000112 | biological_process | 5.11E-04 | regulation of cellular macromolecule biosynthetic process |
| GO:0031326 | biological_process | 5.42E-04 | regulation of cellular biosynthetic process |
| GO:0031323 | biological_process | 5.61E-04 | regulation of cellular metabolic process |
| GO:0036094 | molecular_function | 5.97E-04 | small molecule binding |
| GO:0035639 | molecular_function | 9.66E-04 | purine ribonucleoside triphosphate binding |
| GO:0017076 | molecular_function | 1.37E-03 | purine nucleotide binding |
| GO:0016209 | molecular_function | 1.46E-03 | antioxidant activity |
| GO:0032555 | molecular_function | 1.60E-03 | purine ribonucleotide binding |
| GO:0007186 | biological_process | 2.19E-03 | G protein-coupled receptor signaling pathway |
| GO:1901265 | molecular_function | 2.38E-03 | nucleoside phosphate binding |
| GO:0080090 | biological_process | 2.78E-03 | regulation of primary metabolic process |
| GO:0019222 | biological_process | 2.86E-03 | regulation of metabolic process |
| GO:0051234 | biological_process | 3.14E-03 | establishment of localization |
| GO:0065007 | biological_process | 3.16E-03 | biological regulation |
| GO:0006810 | biological_process | 3.50E-03 | transport |
| GO:0051171 | biological_process | 4.53E-03 | regulation of nitrogen compound metabolic process |
| GO:0060255 | biological_process | 6.11E-03 | regulation of macromolecule metabolic process |
| GO:0010468 | biological_process | 6.82E-03 | regulation of gene expression |
| GO:2001141 | biological_process | 9.23E-03 | regulation of RNA biosynthetic process |
| GO:0008150 | biological_process | 1.10E-02 | biological_process |
| GO:0051252 | biological_process | 1.11E-02 | regulation of RNA metabolic process |
| GO:0005575 | cellular_component | 1.24E-02 | cellular_component |
| GO:0016740 | molecular_function | 1.28E-02 | transferase activity |
| GO:0008152 | biological_process | 1.62E-02 | metabolic process |
| GO:0044238 | biological_process | 1.88E-02 | primary metabolic process |
| GO:0016020 | cellular_component | 2.01E-02 | membrane |

**Table S14** Gene ontology (GO) enrichment analysis of the expanded families in *A. splendens*.

| **GO Terms** | **Type** | **P-value** | **Function** |
| --- | --- | --- | --- |
| GO:0055085 | biological_process | 4.22E-102 | transmembrane transport |
| GO:0032559 | molecular_function | 1.115E-93 | adenyl ribonucleotide binding |
| GO:0030554 | molecular_function | 2.598E-93 | adenyl nucleotide binding |
| GO:0022857 | molecular_function | 1.254E-84 | transmembrane transporter activity |
| GO:0006468 | biological_process | 2.287E-80 | protein phosphorylation |
| GO:0032555 | molecular_function | 2.07E-79 | purine ribonucleotide binding |
| GO:0017076 | molecular_function | 9.975E-79 | purine nucleotide binding |
| GO:0004672 | molecular_function | 1.42E-77 | protein kinase activity |
| GO:0016310 | biological_process | 1.519E-75 | phosphorylation |
| GO:0005215 | molecular_function | 3.774E-75 | transporter activity |
| GO:1901265 | molecular_function | 2.692E-67 | nucleoside phosphate binding |
| GO:0036094 | molecular_function | 7.311E-66 | small molecule binding |
| GO:0016773 | molecular_function | 1.863E-62 | phosphotransferase activity, alcohol group as acceptor |
| GO:0005524 | molecular_function | 2.623E-62 | ATP binding |
| GO:0006796 | biological_process | 6.839E-57 | phosphate-containing compound metabolic process |
| GO:0016301 | molecular_function | 6.206E-56 | kinase activity |
| GO:0035639 | molecular_function | 1.615E-50 | purine ribonucleoside triphosphate binding |
| GO:0016020 | cellular_component | 1.152E-46 | membrane |
| GO:0016772 | molecular_function | 7.345E-46 | transferase activity, transferring phosphorus-containing groups |
| GO:0009987 | biological_process | 3.796E-42 | cellular process |
| GO:0048544 | biological_process | 4.278E-42 | recognition of pollen |
| GO:0036211 | biological_process | 2.245E-40 | protein modification process |
| GO:0022804 | molecular_function | 8.042E-39 | active transmembrane transporter activity |
| GO:0030244 | biological_process | 9.237E-39 | cellulose biosynthetic process |
| GO:0030243 | biological_process | 3.758E-38 | cellulose metabolic process |
| GO:0015079 | molecular_function | 2.561E-36 | potassium ion transmembrane transporter activity |
| GO:0071805 | biological_process | 3.159E-35 | potassium ion transmembrane transport |
| GO:1901363 | molecular_function | 3.759E-35 | heterocyclic compound binding |
| GO:0043412 | biological_process | 3.805E-35 | macromolecule modification |
| GO:0016740 | molecular_function | 7.837E-35 | transferase activity |
| GO:0042626 | molecular_function | 2.764E-34 | ATPase activity, coupled to transmembrane movement of substances |
| GO:0006810 | biological_process | 3.788E-34 | transport |
| GO:0006813 | biological_process | 8.899E-34 | potassium ion transport |
| GO:0051234 | biological_process | 9.123E-34 | establishment of localization |
| GO:0043531 | molecular_function | 2.602E-31 | ADP binding |
| GO:0015405 | molecular_function | 7.875E-31 | P-P-bond-hydrolysis-driven transmembrane transporter activity |
| GO:0044267 | biological_process | 2.078E-29 | cellular protein metabolic process |
| GO:0043492 | molecular_function | 2.956E-29 | ATPase activity, coupled to movement of substances |
| GO:0048610 | biological_process | 1.632E-28 | obsolete cellular process involved in reproduction |
| GO:0016682 | molecular_function | 5.012E-26 | oxidoreductase activity, acting on diphenols and related substances as donors, oxygen as acceptor |
| GO:0022414 | biological_process | 7.051E-26 | reproductive process |
| GO:0019538 | biological_process | 8.312E-23 | protein metabolic process |
| GO:0009250 | biological_process | 2.529E-22 | glucan biosynthetic process |
| GO:0051213 | molecular_function | 2.616E-22 | dioxygenase activity |
| GO:0008150 | biological_process | 4.617E-22 | biological_process |
| GO:0016679 | molecular_function | 6.023E-22 | oxidoreductase activity, acting on diphenols and related substances as donors |
| GO:0044464 | cellular_component | 3.818E-21 | cell part |
| GO:0046914 | molecular_function | 5.787E-20 | transition metal ion binding |
| GO:0052716 | molecular_function | 1.445E-19 | hydroquinone:oxygen oxidoreductase activity |
| GO:0016701 | molecular_function | 2.778E-19 | oxidoreductase activity, acting on single donors with incorporation of molecular oxygen |
| GO:0033692 | biological_process | 2.781E-18 | cellular polysaccharide biosynthetic process |
| GO:0019748 | biological_process | 2.842E-18 | secondary metabolic process |
| GO:0015077 | molecular_function | 3.161E-18 | monovalent inorganic cation transmembrane transporter activity |
| GO:0042623 | molecular_function | 3.781E-18 | ATPase activity, coupled |
| GO:0035251 | molecular_function | 2.381E-17 | UDP-glucosyltransferase activity |
| GO:0046527 | molecular_function | 6.659E-17 | glucosyltransferase activity |
| GO:0044424 | cellular_component | 1.042E-16 | intracellular part |
| GO:0034641 | biological_process | 3.02E-16 | cellular nitrogen compound metabolic process |
| GO:0022834 | molecular_function | 3.072E-16 | ligand-gated channel activity |
| GO:0006807 | biological_process | 4.651E-16 | nitrogen compound metabolic process |
| GO:0015672 | biological_process | 7.442E-16 | monovalent inorganic cation transport |
| GO:0015075 | molecular_function | 1.048E-15 | ion transmembrane transporter activity |
| GO:0004888 | molecular_function | 1.211E-15 | transmembrane signaling receptor activity |
| GO:0006139 | biological_process | 2.951E-15 | nucleobase-containing compound metabolic process |
| GO:0005507 | molecular_function | 6.258E-15 | copper ion binding |
| GO:0046483 | biological_process | 1.298E-14 | heterocycle metabolic process |
| GO:0006073 | biological_process | 1.001E-13 | cellular glucan metabolic process |
| GO:0071704 | biological_process | 1.37E-13 | organic substance metabolic process |
| GO:0044042 | biological_process | 1.457E-13 | glucan metabolic process |
| GO:0004674 | molecular_function | 1.007E-12 | protein serine/threonine kinase activity |
| GO:0044260 | biological_process | 1.444E-12 | cellular macromolecule metabolic process |
| GO:0034220 | biological_process | 7.416E-12 | ion transmembrane transport |
| GO:0032993 | cellular_component | 7.471E-12 | protein-DNA complex |
| GO:0044264 | biological_process | 1.802E-11 | cellular polysaccharide metabolic process |
| GO:0046872 | molecular_function | 2.735E-11 | metal ion binding |
| GO:0005506 | molecular_function | 3.086E-11 | iron ion binding |
| GO:0046873 | molecular_function | 4.621E-11 | metal ion transmembrane transporter activity |
| GO:0090304 | biological_process | 5.678E-11 | nucleic acid metabolic process |
| GO:0000159 | cellular_component | 5.962E-11 | protein phosphatase type 2A complex |
| GO:1901360 | biological_process | 6.994E-11 | organic cyclic compound metabolic process |
| GO:0043170 | biological_process | 9.958E-11 | macromolecule metabolic process |
| GO:0034637 | biological_process | 1.78E-10 | cellular carbohydrate biosynthetic process |
| GO:0016747 | molecular_function | 2.927E-10 | transferase activity, transferring acyl groups other than amino-acyl groups |
| GO:0022890 | molecular_function | 2.93E-10 | inorganic cation transmembrane transporter activity |
| GO:0022838 | molecular_function | 3.168E-10 | substrate-specific channel activity |
| GO:0008287 | cellular_component | 3.182E-10 | protein serine/threonine phosphatase complex |
| GO:0003824 | molecular_function | 3.211E-10 | catalytic activity |
| GO:0006725 | biological_process | 9.547E-10 | cellular aromatic compound metabolic process |
| GO:0016887 | molecular_function | 1.332E-09 | ATPase activity |
| GO:0005976 | biological_process | 1.898E-09 | polysaccharide metabolic process |
| GO:0003674 | molecular_function | 1.996E-09 | molecular_function |
| GO:0043169 | molecular_function | 2.645E-09 | cation binding |
| GO:0030246 | molecular_function | 6.866E-09 | carbohydrate binding |
| GO:0016051 | biological_process | 7.85E-09 | carbohydrate biosynthetic process |
| GO:0019888 | molecular_function | 2.436E-08 | protein phosphatase regulator activity |
| GO:0019439 | biological_process | 3.222E-08 | aromatic compound catabolic process |
| GO:0015291 | molecular_function | 3.862E-08 | secondary active transmembrane transporter activity |
| GO:0015833 | biological_process | 5.292E-08 | peptide transport |
| GO:0006633 | biological_process | 5.484E-08 | fatty acid biosynthetic process |
| GO:0045735 | molecular_function | 7.356E-08 | nutrient reservoir activity |
| GO:0019208 | molecular_function | 8.535E-08 | phosphatase regulator activity |
| GO:1901361 | biological_process | 9.121E-08 | organic cyclic compound catabolic process |
| GO:0022803 | molecular_function | 2.06E-07 | passive transmembrane transporter activity |
| GO:0044444 | cellular_component | 2.146E-07 | cytoplasmic part |
| GO:0016705 | molecular_function | 3.075E-07 | oxidoreductase activity, acting on paired donors, with incorporation or reduction of molecular oxygen |
| GO:0048046 | cellular_component | 3.244E-07 | apoplast |
| GO:0043229 | cellular_component | 2.303E-06 | intracellular organelle |
| GO:0022836 | molecular_function | 4.434E-06 | gated channel activity |
| GO:0006950 | biological_process | 4.961E-06 | response to stress |
| GO:0016021 | cellular_component | 5.186E-06 | integral component of membrane |
| GO:0051015 | molecular_function | 5.681E-06 | actin filament binding |
| GO:0016070 | biological_process | 5.776E-06 | RNA metabolic process |
| GO:0016746 | molecular_function | 7.67E-06 | transferase activity, transferring acyl groups |
| GO:0006811 | biological_process | 1.144E-05 | ion transport |
| GO:0015114 | molecular_function | 1.387E-05 | phosphate ion transmembrane transporter activity |
| GO:0008324 | molecular_function | 1.582E-05 | cation transmembrane transporter activity |
| GO:0031224 | cellular_component | 1.622E-05 | intrinsic component of membrane |
| GO:0038023 | molecular_function | 1.653E-05 | signaling receptor activity |
| GO:0005576 | cellular_component | 1.958E-05 | extracellular region |
| GO:0016788 | molecular_function | 2.206E-05 | hydrolase activity, acting on ester bonds |
| GO:0015297 | molecular_function | 2.44E-05 | antiporter activity |
| GO:0046982 | molecular_function | 2.554E-05 | protein heterodimerization activity |
| GO:0051017 | biological_process | 3.334E-05 | actin filament bundle assembly |
| GO:0008152 | biological_process | 0.0001087 | metabolic process |
| GO:0006631 | biological_process | 0.0001239 | fatty acid metabolic process |
| GO:0043231 | cellular_component | 0.0001318 | intracellular membrane-bounded organelle |
| GO:0051649 | biological_process | 0.0001447 | establishment of localization in cell |
| GO:0044262 | biological_process | 0.0002352 | cellular carbohydrate metabolic process |
| GO:0080019 | molecular_function | 0.0003804 | fatty-acyl-CoA reductase (alcohol-forming) activity |
| GO:0060089 | molecular_function | 0.0006842 | molecular transducer activity |
| GO:0032787 | biological_process | 0.0012009 | monocarboxylic acid metabolic process |
| GO:0003676 | molecular_function | 0.0013015 | nucleic acid binding |
| GO:0020037 | molecular_function | 0.0014166 | heme binding |
| GO:0046906 | molecular_function | 0.0015454 | tetrapyrrole binding |
| GO:0044446 | cellular_component | 0.0016978 | intracellular organelle part |
| GO:0044237 | biological_process | 0.0025747 | cellular metabolic process |
| GO:0006812 | biological_process | 0.0027527 | cation transport |
| GO:0003700 | molecular_function | 0.0034536 | DNA-binding transcription factor activity |
| GO:0004575 | molecular_function | 0.0039615 | sucrose alpha-glucosidase activity |
| GO:0006259 | biological_process | 0.0046054 | DNA metabolic process |
| GO:0044428 | cellular_component | 0.0052748 | nuclear part |
| GO:0055114 | biological_process | 0.0077037 | oxidation-reduction process |
| GO:0005575 | cellular_component | 0.0085298 | cellular_component |
| GO:0042578 | molecular_function | 0.0100291 | phosphoric ester hydrolase activity |
| GO:0015893 | biological_process | 0.0104664 | drug transport |
| GO:0008194 | molecular_function | 0.0133808 | UDP-glycosyltransferase activity |
| GO:0044238 | biological_process | 0.013395 | primary metabolic process |
| GO:0009916 | molecular_function | 0.0145124 | alternative oxidase activity |
| GO:0006396 | biological_process | 0.022939 | RNA processing |
| GO:0032991 | cellular_component | 0.0268674 | protein-containing complex |
| GO:0016491 | molecular_function | 0.0331531 | oxidoreductase activity |
| GO:0016874 | molecular_function | 0.036186 | ligase activity |
| GO:0019843 | molecular_function | 0.0422236 | rRNA binding |

**Table S15** Number of gene pairs and homologous blocks within six Gramineae genomes or between the *A. splendens* and other five genomes.

| Species | Block>4 | Block>10 | Block>20 | Block>50 | LDB | LDBC |
| --- | --- | --- | --- | --- | --- | --- |
| *A. splendens* | 677/15371 | 155/12479 | 104/11721 | 51/10008 | 1211 | Asp2-Asp3 |
| *O. sativa* | 324/3770 | 49/2246 | 16/1790 | 11/1653 | 280 | Os2-Os4 |
| *B. distachyon* | 657/5602 | 75/2370 | 33/1787 | 11/1127 | 265 | Bd2-Bd2 |
| *P. heterocycla* | 842/17197 | 173/13560 | 109/12661 | 66/11269 | 886 | Ped14-Ped16 |
| *A. tauschii* | 295/1985 | 24/529 | 8/295 | 1/101 | 101 | Ata1-Ata3 |
| *H. vulgare* | 464/2926 | 729/660 | 11/440 | 4/242 | 72 | Hv2-Hv6 |
| *A. splendens-P. edulis* | 3168/63930 | 536/49813 | 365/47348 | 216/42499 | 970 | Asp5-Pe15 |
| *A. splendens-O. sativa* | 1553/35753 | 298/29072 | 186/27499 | 107/24828 | 1491 | Asp5-Os3 |
| *A. splendens-B. distachyon* | 1664/35923 | 111/24545 | 195/27265 | 118/24882 | 1074 | Asp5-Bd1 |
| *A. splendens-H. vulgare* | 1788/26546 | 354/18908 | 219/16951 | 98/12896 | 444 | Asp5-Hv4 |
| *A. splendens-A. tauschii* | 1380/26021 | 333/20353 | 224/18725 | 111/14970 | 478 | Asp5-Ata3 |

BL: block_length; LDB: number of colinear gene pairs reside in longest duplicated block

**Table S16 Statistics of duplication types of annotated genes in *A. splendens.***

| Duplication type | Number | Percent |
| --- | --- | --- |
| Nonduplicate gene | 2683 | 4.68% |
| Dispersed duplicate gene | 11176 | 19.48% |
| Nearby duplicate gene | 2112 | 3.68% |
| Tandem repeat gene | 2269 | 3.95% |
| Synteny gene in the WGD region | 31828 | 55.47% |
| Other gene | 7306 | 12.73% |
| Total gene | 57374 | - |

**Table S17** Copy numbers of gene families that are involved in salt-saline tolerance in the *A. thaliana*, *O. sativa, B. distachyon* and *A. splendens* genomes.

| Gene Family | No. of genes | | | |  | Gene ratio | |
| --- | --- | --- | --- | --- | --- | --- | --- |
|  | *A. thaliana (AT)* | *O. sativa (OS)* | *B. distachyon (BD)* | *A. splendens (AS)* |  | *AS/OS* | *AS/BD* |
| NHX | 8 | 7 | 9 | 16 |  | 2.29 | 1.78 |
| KEA | 6 | 4 | 5 | 9 |  | 2.25 | 1.80 |
| CHX | 27 | 17 | 15 | 24 |  | 1.41 | 1.60 |
| HKT1 | 1 | 7 | 6 | 8 |  | 1.14 | 1.33 |
| Shaker | 9 | 11 | 10 | 16 |  | 1.45 | 1.60 |
| KUP-HAK-KT | 13 | 26 | 27 | 53 |  | 2.04 | 1.96 |
| CNGC | 20 | 16 | 16 | 29 |  | 1.81 | 1.81 |
| TPK | 6 | 3 | 4 | 6 |  | 2.00 | 1.50 |
| Ppa | 6 | 8 | 7 | 16 |  | 2.00 | 2.29 |
| AHA | 21 | 21 | 16 | 34 |  | 1.62 | 2.13 |
| ACA | 18 | 15 | 20 | 28 |  | 1.87 | 1.40 |
| ECA | 26 | 19 | 23 | 38 |  | 2.00 | 1.65 |
| CAX | 6 | 6 | 6 | 11 |  | 1.83 | 1.83 |
| AVP | 3 | 7 | 5 | 16 |  | 2.29 | 3.20 |
| GLR | 20 | 21 | 18 | 26 |  | 1.24 | 1.44 |
| CCC | 1 | 2 | 3 | 3 |  | 1.50 | 1.00 |
| CIPK | 26 | 34 | 30 | 60 |  | 1.76 | 2.00 |
| CDPK | 26 | 33 | 29 | 47 |  | 1.42 | 1.62 |
| Average | - | - | - | - |  | 1.77 | 1.77 |

**Table S18** Copy numbers of transcription factor families identified in the *A. thaliana*, *O. sativa, B. distachyon* and *A. splendens* genomes.

| Gene Family | No. of genes | | | | |  | Gene ratio | |
| --- | --- | --- | --- | --- | --- | --- | --- | --- |
|  | *A. thaliana (AT)* | *O. sativa (OS)* | *B. distachyon (BD)* | *A. splendens (AS)* |  | | *AS/OS* | *AS/BD* |
| AP2 | 18 | 16 | 38 | 54 |  | | 3.38 | 1.42 |
| ARF | 22 | 27 | 26 | 45 |  | | 1.67 | 1.73 |
| ARR-B | 14 | 9 | 13 | 12 |  | | 1.33 | 0.92 |
| B3 | 66 | 54 | 54 | 82 |  | | 1.52 | 1.52 |
| BBR-BPC | 7 | 4 | 3 | 6 |  | | 1.50 | 2.00 |
| BES1 | 8 | 6 | 8 | 12 |  | | 2.00 | 1.50 |
| C2H2 | 100 | 106 | 96 | 155 |  | | 1.46 | 1.61 |
| C3H | 49 | 49 | 47 | 93 |  | | 1.90 | 1.98 |
| CAMTA | 6 | 6 | 7 | 13 |  | | 2.17 | 1.86 |
| CO-like | 17 | 15 | 13 | 19 |  | | 1.27 | 1.46 |
| CPP | 8 | 11 | 9 | 18 |  | | 1.64 | 2.00 |
| DBB | 8 | 10 | 9 | 7 |  | | 0.70 | 0.78 |
| Dof | 36 | 30 | 29 | 49 |  | | 1.63 | 1.69 |
| E2F/DP | 8 | 7 | 11 | 20 |  | | 2.86 | 1.82 |
| EIL | 6 | 9 | 6 | 16 |  | | 1.78 | 2.67 |
| ERF | 122 | 138 | 138 | 206 |  | | 1.49 | 1.49 |
| G2-like | 42 | 47 | 51 | 91 |  | | 1.94 | 1.78 |
| GATA | 30 | 25 | 29 | 50 |  | | 2.00 | 1.72 |
| GRAS | 34 | 60 | 63 | 115 |  | | 1.92 | 1.83 |
| GRF | 9 | 12 | 12 | 22 |  | | 1.83 | 1.83 |
| GeBP | 22 | 13 | 13 | 25 |  | | 1.92 | 1.92 |
| HB-PHD | 2 | 1 | 3 | 5 |  | | 5.00 | 1.67 |
| HB-other | 7 | 11 | 13 | 32 |  | | 2.91 | 2.46 |
| HD-ZIP | 48 | 42 | 42 | 74 |  | | 1.76 | 1.76 |
| HRT-like | 2 | 1 | 1 | 1 |  | | 1.00 | 1.00 |
| LBD | 43 | 36 | 28 | 54 |  | | 1.50 | 1.93 |
| LFY | 1 | 2 | 1 | 2 |  | | 1.00 | 2.00 |
| LSD | 3 | 5 | 5 | 5 |  | | 1.00 | 1.00 |
| M-type_MADS | 66 | 32 | 45 | 13 |  | | 0.41 | 0.29 |
| MYB | 144 | 122 | 123 | 204 |  | | 1.67 | 1.66 |
| MYB_related | 61 | 66 | 61 | 139 |  | | 2.11 | 2.28 |
| NAC | 112 | 140 | 136 | 249 |  | | 1.78 | 1.83 |
| NF-X1 | 2 | 2 | 2 | 3 |  | | 1.50 | 1.50 |
| NF-YA | 10 | 11 | 7 | 16 |  | | 1.45 | 2.29 |
| NF-YB | 13 | 13 | 18 | 34 |  | | 2.62 | 1.89 |
| NF-YC | 14 | 16 | 16 | 21 |  | | 1.31 | 1.31 |
| Nin-like | 14 | 13 | 16 | 24 |  | | 1.85 | 1.50 |
| RAV | 6 | 4 | 4 | 6 |  | | 1.50 | 1.50 |
| S1Fa-like | 3 | 2 | 1 | 2 |  | | 1.00 | 2.00 |
| SBP | 17 | 19 | 17 | 37 |  | | 1.95 | 2.18 |
| SRS | 11 | 5 | 6 | 7 |  | | 1.40 | 1.17 |
| STAT | 2 | 1 | 1 | 2 |  | | 2.00 | 2.00 |
| TALE | 21 | 26 | 22 | 42 |  | | 1.62 | 1.91 |
| TCP | 24 | 20 | 21 | 19 |  | | 0.95 | 0.90 |
| Trihelix | 39 | 31 | 30 | 49 |  | | 1.58 | 1.63 |
| VOZ | 2 | 2 | 2 | 4 |  | | 2.00 | 2.00 |
| WOX | 16 | 14 | 13 | 19 |  | | 1.36 | 1.46 |
| WRKY | 72 | 94 | 88 | 117 |  | | 1.24 | 1.33 |
| Whirly | 3 | 2 | 2 | 4 |  | | 2.00 | 2.00 |
| YABBY | 6 | 8 | 8 | 15 |  | | 1.88 | 1.88 |
| ZF-HD | 17 | 14 | 21 | 19 |  | | 1.36 | 0.90 |
| bHLH | 153 | 156 | 147 | 265 |  | | 1.70 | 1.80 |
| bZIP | 74 | 95 | 86 | 154 |  | | 1.62 | 1.79 |
| Average | - | - | - | - |  | | 1.73 | 1.67 |

**Table S19** The present pattern of DEGs in each of the total 6,796 paralogous gene (i.e., two copies in *A. splendens* with one synteny copy in *O. sativa*) pairs in *A. splendens*.

| Pattern | Number | Percent |
| --- | --- | --- |
| No DEG | 5,347 | 78.68% |
| One DEG in each paralogous gene pair | 1,047 | 15.40% |
| Both are DEGs in each paralogous gene pair | 402 | 5.92% |

**Table S20** The differential expression pattern of 402 paralogous gene pairs in both root and shoot.

|  |  | Log_2_(Fold change) | | | |  |  | Log_2_(Fold change) | | | |
| --- | --- | --- | --- | --- | --- | --- | --- | --- | --- | --- | --- |
| Pattern | Paralogous copy 1 | 6h_root | 24h_root | 6h_shoot | 24h_shoot |  | Paralogous copy 2 | 6h_root | 24h_root | 6h_shoot | 24h_shoot |
| I (258) | AspG014206 | 0.00 | 0.00 | 0.00 | 2.80 |  | AspG003371 | 0.00 | 0.00 | 0.00 | 2.12 |
| I | AspG014201 | 2.64 | 0.00 | 0.00 | 0.00 |  | AspG052847 | 3.50 | 0.00 | 0.00 | 0.00 |
| I | AspG014163 | 0.00 | 0.00 | 0.00 | 5.23 |  | AspG050166 | 0.00 | 0.00 | 0.00 | 3.86 |
| I | AspG014152 | 0.00 | 0.00 | 0.00 | -2.65 |  | AspG050154 | 0.00 | 0.00 | 0.00 | -2.94 |
| I | AspG014112 | -3.77 | -2.72 | 0.00 | 0.00 |  | AspG020106 | -3.94 | -2.08 | 0.00 | 0.00 |
| I | AspG014107 | 0.00 | 0.00 | 0.00 | -2.28 |  | AspG020102 | 0.00 | 0.00 | -2.25 | -2.38 |
| I | AspG024006 | 0.00 | 0.00 | 0.00 | 2.05 |  | AspG050428 | 0.00 | 0.00 | 0.00 | 2.96 |
| I | AspG028490 | 0.00 | 0.00 | 0.00 | -2.26 |  | AspG040892 | 0.00 | 0.00 | -2.08 | -2.41 |
| I | AspG047484 | 4.68 | 3.96 | 0.00 | 0.00 |  | AspG042478 | 4.17 | 4.19 | 0.00 | 0.00 |
| I | AspG004022 | 2.16 | 0.00 | 2.43 | 4.20 |  | AspG035234 | 2.64 | 0.00 | 2.47 | 2.86 |
| I | AspG003921 | 0.00 | 0.00 | 0.00 | 3.62 |  | AspG035197 | 0.00 | 0.00 | 0.00 | 2.05 |
| I | AspG017526 | 0.00 | 0.00 | 0.00 | -3.68 |  | AspG019922 | 0.00 | 0.00 | 0.00 | -3.00 |
| I | AspG017556 | 3.00 | 2.06 | 0.00 | 0.00 |  | AspG008895 | 2.43 | 0.00 | 0.00 | 0.00 |
| I | AspG054249 | 0.00 | -3.25 | 0.00 | 0.00 |  | AspG008893 | -2.50 | -2.83 | 0.00 | 0.00 |
| I | AspG034465 | 0.00 | 2.90 | 0.00 | 0.00 |  | AspG052077 | 3.53 | 2.56 | 0.00 | 0.00 |
| I | AspG017171 | -3.81 | -2.23 | 0.00 | 0.00 |  | AspG025098 | -3.32 | 0.00 | 0.00 | 0.00 |
| I | AspG035870 | -5.38 | -6.53 | 0.00 | 0.00 |  | AspG057078 | -2.96 | -4.47 | 0.00 | 0.00 |
| I | AspG057420 | 0.00 | 0.00 | 0.00 | 2.21 |  | AspG046290 | 0.00 | 0.00 | 0.00 | 2.84 |
| I | AspG004448 | 2.20 | 0.00 | 0.00 | 0.00 |  | AspG024228 | 2.60 | 2.38 | 0.00 | 0.00 |
| I | AspG027931 | 0.00 | -2.24 | 0.00 | 0.00 |  | AspG044431 | 0.00 | -2.16 | 0.00 | 0.00 |
| I | AspG027928 | 0.00 | 0.00 | 0.00 | 2.69 |  | AspG044391 | 0.00 | 0.00 | 0.00 | 2.33 |
| I | AspG025024 | -8.02 | -5.85 | -3.24 | -2.52 |  | AspG029243 | -7.94 | -5.45 | -2.93 | 0.00 |
| I | AspG009711 | -6.22 | -5.37 | -4.39 | -4.98 |  | AspG046085 | -6.15 | -5.49 | -3.85 | -4.26 |
| I | AspG049916 | 0.00 | 0.00 | 0.00 | 3.29 |  | AspG042816 | 0.00 | 0.00 | 0.00 | 2.31 |
| I | AspG052808 | 3.82 | 5.25 | 0.00 | 0.00 |  | AspG033791 | 0.00 | 3.29 | 0.00 | 0.00 |
| I | AspG027433 | 0.00 | 0.00 | 0.00 | 3.01 |  | AspG033746 | 0.00 | 0.00 | 0.00 | 2.04 |
| I | AspG022312 | -2.42 | -3.52 | 0.00 | -3.45 |  | AspG039766 | 0.00 | -2.03 | -2.28 | -3.70 |
| I | AspG039028 | 3.18 | 3.12 | 0.00 | 0.00 |  | AspG048709 | 4.44 | 3.94 | 0.00 | 0.00 |
| I | AspG040094 | -2.61 | -2.48 | 0.00 | 0.00 |  | AspG021307 | 0.00 | -3.92 | 0.00 | 0.00 |
| I | AspG046208 | 0.00 | 0.00 | -2.36 | -3.73 |  | AspG021341 | 0.00 | 0.00 | -2.26 | -3.39 |
| I | AspG017352 | -3.46 | -2.37 | 0.00 | 0.00 |  | AspG050204 | -2.41 | 0.00 | 0.00 | 0.00 |
| I | AspG040147 | -4.46 | -5.52 | 0.00 | 0.00 |  | AspG050185 | 0.00 | -2.61 | 0.00 | 0.00 |
| I | AspG046405 | -4.11 | -5.64 | 0.00 | 0.00 |  | AspG053517 | -3.43 | -4.15 | 0.00 | 0.00 |
| I | AspG046414 | 2.41 | 0.00 | 0.00 | 0.00 |  | AspG053510 | 3.55 | 4.59 | 0.00 | 0.00 |
| I | AspG003982 | 0.00 | 0.00 | 0.00 | -2.13 |  | AspG051957 | 0.00 | 0.00 | -2.37 | -2.20 |
| I | AspG005734 | -3.58 | -4.96 | 0.00 | 0.00 |  | AspG024726 | 0.00 | -2.78 | 0.00 | 0.00 |
| I | AspG035800 | 0.00 | 0.00 | 0.00 | 2.01 |  | AspG056335 | 0.00 | 0.00 | 0.00 | 2.37 |
| I | AspG035861 | 0.00 | 0.00 | 0.00 | 2.79 |  | AspG056338 | 0.00 | 0.00 | 0.00 | 3.41 |
| I | AspG055200 | 3.05 | 0.00 | 0.00 | 0.00 |  | AspG052504 | 0.00 | 2.04 | 0.00 | 0.00 |
| I | AspG032760 | 4.04 | 2.93 | 0.00 | 0.00 |  | AspG053415 | 6.40 | 4.95 | 0.00 | 0.00 |
| I | AspG000336 | 0.00 | -2.88 | 0.00 | 0.00 |  | AspG053451 | 0.00 | -4.68 | 0.00 | 0.00 |
| I | AspG000336 | 0.00 | -2.88 | 0.00 | 0.00 |  | AspG053451 | 0.00 | -4.68 | 0.00 | 0.00 |
| I | AspG000336 | 0.00 | -2.88 | 0.00 | 0.00 |  | AspG053451 | 0.00 | -4.68 | 0.00 | 0.00 |
| I | AspG000279 | 0.00 | 0.00 | 3.64 | 2.18 |  | AspG047035 | 0.00 | 0.00 | 2.35 | 0.00 |
| I | AspG000159 | 0.00 | 0.00 | 0.00 | 2.39 |  | AspG035018 | 0.00 | 0.00 | 0.00 | 2.31 |
| I | AspG023646 | 4.17 | 3.16 | -2.98 | -4.63 |  | AspG029855 | 3.74 | 0.00 | -3.59 | -6.01 |
| I | AspG023728 | -4.35 | -3.58 | 0.00 | 0.00 |  | AspG052297 | -4.57 | -3.85 | 0.00 | 0.00 |
| I | AspG011488 | 0.00 | 0.00 | 0.00 | 2.18 |  | AspG009512 | 0.00 | 0.00 | 0.00 | 2.49 |
| I | AspG011606 | 0.00 | 0.00 | 0.00 | 2.15 |  | AspG009525 | 0.00 | 0.00 | 0.00 | 2.80 |
| I | AspG032504 | 0.00 | 0.00 | 0.00 | 2.83 |  | AspG037011 | 0.00 | 0.00 | 0.00 | 5.23 |
| I | AspG032496 | 0.00 | 2.91 | 0.00 | 4.79 |  | AspG037014 | 0.00 | 2.49 | 0.00 | 3.81 |
| I | AspG006275 | 0.00 | -2.13 | 0.00 | 0.00 |  | AspG008179 | -2.20 | -2.72 | 0.00 | 0.00 |
| I | AspG049405 | -3.41 | -3.52 | 0.00 | 0.00 |  | AspG002042 | 0.00 | -3.57 | 0.00 | 0.00 |
| I | AspG049400 | 0.00 | 0.00 | 0.00 | 2.27 |  | AspG029400 | 0.00 | 0.00 | 0.00 | 2.15 |
| I | AspG035168 | 0.00 | 0.00 | 0.00 | 2.00 |  | AspG029395 | 0.00 | 0.00 | 0.00 | 2.14 |
| I | AspG020793 | 4.06 | 4.05 | 0.00 | 0.00 |  | AspG041051 | 3.27 | 2.07 | 0.00 | 0.00 |
| I | AspG022488 | -5.86 | -5.40 | -4.51 | -2.96 |  | AspG042309 | -2.32 | -2.19 | -3.74 | -3.31 |
| I | AspG002094 | -2.63 | -2.63 | 0.00 | 0.00 |  | AspG036349 | -2.38 | 0.00 | 0.00 | 0.00 |
| I | AspG029543 | 0.00 | 2.27 | 0.00 | 0.00 |  | AspG025429 | 0.00 | 2.26 | 0.00 | 0.00 |
| I | AspG054461 | 0.00 | 0.00 | 0.00 | 2.48 |  | AspG007892 | 0.00 | 0.00 | 0.00 | 2.13 |
| I | AspG054524 | -3.49 | -3.39 | 0.00 | 0.00 |  | AspG007907 | -2.88 | 0.00 | 0.00 | 0.00 |
| I | AspG033335 | 0.00 | 0.00 | 0.00 | 8.14 |  | AspG034039 | 0.00 | 0.00 | 0.00 | 6.24 |
| I | AspG009180 | -6.04 | -4.19 | -2.23 | -2.11 |  | AspG053133 | -6.34 | -5.28 | -2.37 | -2.82 |
| I | AspG023159 | 3.82 | 4.45 | 0.00 | 0.00 |  | AspG034310 | 2.37 | 3.29 | 0.00 | 0.00 |
| I | AspG047183 | -2.41 | -3.84 | 0.00 | 0.00 |  | AspG004607 | 0.00 | -2.76 | 0.00 | 0.00 |
| I | AspG047268 | 4.46 | 0.00 | 0.00 | 0.00 |  | AspG055609 | 3.43 | 2.34 | 0.00 | 0.00 |
| I | AspG047245 | 0.00 | -2.57 | 0.00 | 0.00 |  | AspG034604 | 0.00 | -2.27 | 0.00 | 0.00 |
| I | AspG056054 | -2.20 | -3.89 | 0.00 | 0.00 |  | AspG005135 | 0.00 | -3.37 | 0.00 | 0.00 |
| I | AspG056141 | -2.99 | -2.75 | 0.00 | 0.00 |  | AspG051620 | -3.59 | 0.00 | 0.00 | 0.00 |
| I | AspG050516 | 0.00 | 0.00 | 0.00 | 2.97 |  | AspG007778 | 0.00 | 0.00 | 0.00 | 2.88 |
| I | AspG050608 | 0.00 | 0.00 | 0.00 | -3.70 |  | AspG004690 | 0.00 | 0.00 | -2.19 | -2.01 |
| I | AspG050600 | 0.00 | 0.00 | 0.00 | 2.03 |  | AspG004683 | 0.00 | 0.00 | 0.00 | 3.59 |
| I | AspG051388 | -2.92 | -2.97 | 0.00 | 0.00 |  | AspG008772 | -3.31 | -2.91 | 0.00 | 0.00 |
| I | AspG051380 | 0.00 | 2.04 | 0.00 | 0.00 |  | AspG008782 | 0.00 | 2.44 | 0.00 | 0.00 |
| I | AspG034150 | 2.33 | 0.00 | 0.00 | 0.00 |  | AspG028438 | 2.21 | 0.00 | 0.00 | 0.00 |
| I | AspG034080 | 0.00 | -2.41 | 0.00 | 0.00 |  | AspG028320 | 0.00 | -2.57 | 0.00 | 0.00 |
| I | AspG021627 | 6.76 | 6.88 | 0.00 | 0.00 |  | AspG056627 | 3.91 | 5.19 | 0.00 | 0.00 |
| I | AspG021585 | 2.16 | 0.00 | 0.00 | 0.00 |  | AspG056725 | 2.05 | 0.00 | 0.00 | 0.00 |
| I | AspG013320 | 0.00 | 0.00 | 0.00 | 2.25 |  | AspG043021 | 0.00 | 0.00 | 0.00 | 2.47 |
| I | AspG049071 | -5.11 | -3.50 | -3.82 | -3.19 |  | AspG047531 | -6.60 | -5.88 | -2.36 | 0.00 |
| I | AspG032336 | -2.06 | 0.00 | 0.00 | 0.00 |  | AspG021782 | -2.13 | 0.00 | 0.00 | 0.00 |
| I | AspG032314 | 0.00 | 0.00 | 0.00 | 3.83 |  | AspG021804 | 0.00 | 0.00 | 0.00 | 2.82 |
| I | AspG032462 | 0.00 | 0.00 | 0.00 | 3.10 |  | AspG021676 | 0.00 | 0.00 | 0.00 | 4.82 |
| I | AspG032419 | -5.92 | -5.95 | -4.43 | -3.54 |  | AspG013880 | -5.02 | -4.17 | -3.19 | -2.66 |
| I | AspG047287 | 2.02 | 3.35 | 0.00 | 0.00 |  | AspG007234 | 0.00 | 2.87 | 0.00 | 0.00 |
| I | AspG001629 | 0.00 | 0.00 | 0.00 | 2.52 |  | AspG007451 | 0.00 | 0.00 | 0.00 | 5.69 |
| I | AspG001648 | 4.08 | 0.00 | 0.00 | 9.27 |  | AspG007466 | 2.80 | 0.00 | 0.00 | 5.89 |
| I | AspG001658 | 2.13 | 2.90 | 0.00 | 0.00 |  | AspG007478 | 0.00 | 2.04 | 0.00 | 0.00 |
| I | AspG001676 | -2.17 | 0.00 | 0.00 | 0.00 |  | AspG007239 | -2.07 | 0.00 | 0.00 | 0.00 |
| I | AspG010202 | -4.21 | -5.07 | 0.00 | 0.00 |  | AspG007324 | -2.60 | -4.09 | 0.00 | 0.00 |
| I | AspG010165 | 3.62 | 0.00 | 0.00 | 0.00 |  | AspG022911 | 2.41 | 0.00 | 0.00 | 0.00 |
| I | AspG043675 | 3.15 | 0.00 | 0.00 | 0.00 |  | AspG022930 | 3.11 | 2.44 | 0.00 | 0.00 |
| I | AspG013046 | 0.00 | 0.00 | -2.27 | -2.87 |  | AspG050675 | 0.00 | 0.00 | -2.76 | -3.24 |
| I | AspG013092 | 0.00 | -2.50 | 0.00 | 0.00 |  | AspG050672 | 0.00 | -2.70 | 0.00 | 0.00 |
| I | AspG013094 | 2.30 | 3.40 | 0.00 | 0.00 |  | AspG013998 | 0.00 | 2.38 | 0.00 | 0.00 |
| I | AspG009058 | 0.00 | 2.51 | 0.00 | 0.00 |  | AspG013990 | 0.00 | 2.28 | 0.00 | 0.00 |
| I | AspG049458 | 0.00 | 3.36 | 0.00 | 0.00 |  | AspG001487 | 3.17 | 3.07 | 0.00 | 0.00 |
| I | AspG049470 | 0.00 | 0.00 | 0.00 | 2.62 |  | AspG007762 | 0.00 | 0.00 | 0.00 | 2.74 |
| I | AspG049522 | -2.41 | 0.00 | 0.00 | 0.00 |  | AspG007698 | -2.58 | 0.00 | 0.00 | 0.00 |
| I | AspG049525 | 2.51 | 0.00 | 0.00 | 0.00 |  | AspG007692 | 5.89 | 6.39 | 0.00 | 0.00 |
| I | AspG049365 | -2.76 | 0.00 | 0.00 | 0.00 |  | AspG015591 | -2.79 | 0.00 | 0.00 | 0.00 |
| I | AspG049359 | -2.49 | -2.04 | 0.00 | 0.00 |  | AspG015596 | -3.49 | -2.42 | 0.00 | 0.00 |
| I | AspG049345 | 2.63 | 2.49 | 0.00 | 0.00 |  | AspG015626 | 0.00 | 2.46 | 0.00 | 0.00 |
| I | AspG026065 | 0.00 | 0.00 | 0.00 | -3.46 |  | AspG015641 | 0.00 | 0.00 | -3.66 | -4.72 |
| I | AspG026071 | 0.00 | -3.21 | 0.00 | 0.00 |  | AspG015643 | 0.00 | -3.86 | 0.00 | 0.00 |
| I | AspG015888 | 2.35 | 2.01 | 0.00 | 2.39 |  | AspG048025 | 2.86 | 2.54 | 3.07 | 3.56 |
| I | AspG015862 | -3.73 | 0.00 | 0.00 | -3.07 |  | AspG036438 | -7.09 | -4.75 | 0.00 | -2.26 |
| I | AspG012122 | 0.00 | 0.00 | 0.00 | 2.20 |  | AspG033160 | 0.00 | 0.00 | 0.00 | 2.52 |
| I | AspG012165 | 0.00 | 0.00 | 0.00 | -2.17 |  | AspG033103 | -2.06 | 0.00 | 0.00 | -2.61 |
| I | AspG012176 | -4.47 | -6.24 | 0.00 | -3.80 |  | AspG000608 | -4.20 | -5.68 | 0.00 | -3.63 |
| I | AspG052920 | 3.43 | 2.19 | 0.00 | 0.00 |  | AspG000658 | 2.85 | 0.00 | 0.00 | 0.00 |
| I | AspG018212 | 0.00 | 0.00 | 0.00 | 2.24 |  | AspG031567 | 0.00 | 0.00 | 0.00 | 2.02 |
| I | AspG019061 | 2.23 | 0.00 | 0.00 | 0.00 |  | AspG000955 | 0.00 | 2.33 | 0.00 | 0.00 |
| I | AspG019094 | 0.00 | 2.11 | 0.00 | 0.00 |  | AspG000951 | 0.00 | 2.93 | 0.00 | 0.00 |
| I | AspG019108 | 0.00 | 0.00 | 0.00 | 2.58 |  | AspG000942 | 0.00 | 0.00 | 0.00 | 3.03 |
| I | AspG019131 | -4.19 | -4.69 | -2.79 | -2.86 |  | AspG006277 | -5.11 | -6.25 | 0.00 | -2.03 |
| I | AspG019134 | 0.00 | 0.00 | 0.00 | 2.28 |  | AspG027079 | 0.00 | 0.00 | 0.00 | 2.69 |
| I | AspG019141 | 0.00 | 0.00 | 0.00 | 2.58 |  | AspG027075 | 0.00 | 0.00 | 0.00 | 3.00 |
| I | AspG002034 | 0.00 | 0.00 | 0.00 | 2.90 |  | AspG027048 | 0.00 | 0.00 | 0.00 | 2.52 |
| I | AspG054758 | -4.81 | -4.31 | 0.00 | 0.00 |  | AspG045377 | -2.69 | -2.83 | 0.00 | 0.00 |
| I | AspG017636 | 4.96 | 5.67 | 0.00 | 0.00 |  | AspG052178 | 5.48 | 0.00 | 0.00 | 0.00 |
| I | AspG028703 | 2.00 | 0.00 | 0.00 | 0.00 |  | AspG003582 | 3.42 | 2.83 | 0.00 | 0.00 |
| I | AspG055510 | -2.60 | -2.95 | 0.00 | 0.00 |  | AspG034193 | -3.73 | -4.95 | 0.00 | 0.00 |
| I | AspG011879 | -3.89 | -4.18 | -3.23 | -5.44 |  | AspG045428 | 0.00 | -4.44 | -3.76 | -6.54 |
| I | AspG020002 | -3.47 | 0.00 | 0.00 | 0.00 |  | AspG036926 | -2.30 | 0.00 | 0.00 | 0.00 |
| I | AspG048139 | 0.00 | 0.00 | 0.00 | 2.22 |  | AspG007129 | 0.00 | 0.00 | 0.00 | 2.57 |
| I | AspG018157 | 0.00 | 0.00 | 0.00 | 2.97 |  | AspG024555 | 0.00 | 0.00 | 0.00 | 2.27 |
| I | AspG018153 | 0.00 | 0.00 | 0.00 | 2.45 |  | AspG024559 | 0.00 | 0.00 | 0.00 | 3.45 |
| I | AspG018139 | 0.00 | 0.00 | 0.00 | 2.25 |  | AspG024565 | 0.00 | 0.00 | 0.00 | 3.41 |
| I | AspG027512 | 0.00 | 0.00 | 0.00 | 3.25 |  | AspG024483 | 0.00 | 0.00 | 0.00 | 2.11 |
| I | AspG022170 | 4.17 | 3.95 | 0.00 | 0.00 |  | AspG056891 | 2.32 | 0.00 | 0.00 | 0.00 |
| I | AspG039668 | 0.00 | 0.00 | 0.00 | 3.96 |  | AspG017586 | 0.00 | 0.00 | 0.00 | 2.67 |
| I | AspG034871 | 0.00 | 3.25 | 0.00 | 0.00 |  | AspG005123 | 0.00 | 3.38 | 0.00 | 0.00 |
| I | AspG010661 | 0.00 | 0.00 | 0.00 | 3.06 |  | AspG030657 | 0.00 | 0.00 | 0.00 | 4.42 |
| I | AspG037539 | -2.76 | 0.00 | 0.00 | 0.00 |  | AspG014651 | -2.40 | 0.00 | 0.00 | 0.00 |
| I | AspG037546 | 0.00 | 0.00 | 0.00 | 2.88 |  | AspG014656 | 0.00 | 0.00 | 0.00 | 2.14 |
| I | AspG055723 | 2.81 | 0.00 | 0.00 | 0.00 |  | AspG053948 | 0.00 | 0.00 | 0.00 | 5.67 |
| I | AspG044517 | 0.00 | 0.00 | 0.00 | 2.22 |  | AspG057626 | 0.00 | 0.00 | 0.00 | 2.19 |
| I | AspG036539 | 0.00 | -2.08 | 0.00 | 0.00 |  | AspG009956 | 0.00 | -2.43 | 0.00 | 0.00 |
| I | AspG019691 | 0.00 | -2.07 | 0.00 | 0.00 |  | AspG017267 | 0.00 | -3.10 | 0.00 | 0.00 |
| I | AspG055280 | 0.00 | -2.62 | 0.00 | 0.00 |  | AspG028616 | -2.90 | -4.22 | 0.00 | 0.00 |
| I | AspG028602 | 0.00 | -3.02 | 0.00 | 0.00 |  | AspG028615 | -3.36 | -4.55 | 0.00 | 0.00 |
| I | AspG045061 | 0.00 | 0.00 | 0.00 | 2.83 |  | AspG030460 | 0.00 | 0.00 | 0.00 | 2.69 |
| I | AspG044705 | 0.00 | 2.67 | 0.00 | 0.00 |  | AspG015242 | 2.77 | 3.83 | 0.00 | 0.00 |
| I | AspG044741 | 2.44 | 3.64 | 0.00 | 0.00 |  | AspG015240 | 3.43 | 3.91 | 0.00 | 0.00 |
| I | AspG044705 | 0.00 | 2.67 | 0.00 | 0.00 |  | AspG015242 | 2.77 | 3.83 | 0.00 | 0.00 |
| I | AspG042648 | -3.52 | -3.94 | 0.00 | -2.74 |  | AspG015249 | -4.60 | -4.99 | 0.00 | -2.19 |
| I | AspG042684 | -2.55 | 0.00 | -2.06 | -2.63 |  | AspG032998 | -2.98 | -2.27 | -2.86 | -3.49 |
| I | AspG025917 | 2.05 | 0.00 | 0.00 | 0.00 |  | AspG046868 | 2.36 | 0.00 | 0.00 | 0.00 |
| I | AspG016825 | 0.00 | -4.86 | 0.00 | -2.36 |  | AspG016506 | -3.04 | -4.70 | 0.00 | -3.00 |
| I | AspG049218 | -2.37 | 0.00 | 0.00 | 0.00 |  | AspG027763 | -2.64 | -2.02 | 0.00 | 0.00 |
| I | AspG038834 | 0.00 | 0.00 | 0.00 | 2.16 |  | AspG010397 | 0.00 | 0.00 | 0.00 | 2.00 |
| I | AspG005505 | 2.26 | 2.45 | 0.00 | 0.00 |  | AspG030074 | 2.20 | 2.38 | 0.00 | 0.00 |
| I | AspG024600 | -2.36 | 0.00 | 0.00 | 0.00 |  | AspG006368 | -2.12 | -3.27 | 0.00 | 0.00 |
| I | AspG048118 | -2.26 | -2.16 | 0.00 | 0.00 |  | AspG005070 | 0.00 | -2.07 | 0.00 | 0.00 |
| I | AspG026769 | -3.28 | -3.82 | 0.00 | 0.00 |  | AspG048941 | -4.50 | -4.31 | 0.00 | 0.00 |
| I | AspG028545 | 0.00 | 2.37 | 0.00 | 0.00 |  | AspG038225 | 2.67 | 3.23 | 0.00 | 0.00 |
| I | AspG057276 | 2.93 | 0.00 | 0.00 | 4.41 |  | AspG018853 | 2.79 | 0.00 | 0.00 | 4.46 |
| I | AspG057288 | 3.46 | 2.24 | 0.00 | 0.00 |  | AspG018842 | 2.47 | 0.00 | 0.00 | 0.00 |
| I | AspG038756 | 2.88 | 2.74 | 0.00 | 0.00 |  | AspG040827 | 0.00 | 2.58 | 0.00 | 0.00 |
| I | AspG038765 | 0.00 | 0.00 | 0.00 | 2.23 |  | AspG040836 | 0.00 | 0.00 | 0.00 | 4.47 |
| I | AspG038807 | 2.14 | 0.00 | 0.00 | 0.00 |  | AspG047988 | 2.40 | 2.11 | 0.00 | 0.00 |
| I | AspG044483 | 0.00 | 0.00 | 0.00 | 3.08 |  | AspG054221 | 0.00 | 0.00 | 0.00 | 2.84 |
| I | AspG037683 | 0.00 | -2.72 | 0.00 | 0.00 |  | AspG039823 | 0.00 | -2.44 | 0.00 | 0.00 |
| I | AspG037647 | -2.07 | -2.41 | 0.00 | -3.22 |  | AspG011072 | -2.39 | -3.07 | -2.69 | -3.83 |
| I | AspG037767 | 0.00 | -2.61 | 0.00 | 0.00 |  | AspG019565 | 0.00 | -2.56 | 0.00 | 0.00 |
| I | AspG037757 | 0.00 | 0.00 | 0.00 | -2.36 |  | AspG056015 | 0.00 | 0.00 | 0.00 | -2.74 |
| I | AspG037753 | 0.00 | 0.00 | 2.60 | 6.10 |  | AspG000754 | 0.00 | 0.00 | 0.00 | 4.21 |
| I | AspG037740 | 0.00 | 0.00 | 0.00 | 5.30 |  | AspG000743 | 0.00 | 0.00 | 0.00 | 6.13 |
| I | AspG051735 | 3.34 | 5.63 | 0.00 | 0.00 |  | AspG048094 | 4.50 | 5.93 | 0.00 | 0.00 |
| I | AspG009798 | 3.18 | 3.14 | 0.00 | 0.00 |  | AspG021048 | 0.00 | 2.26 | 0.00 | 0.00 |
| I | AspG001050 | -3.00 | -3.35 | -3.69 | -3.93 |  | AspG043714 | -4.67 | -3.88 | -6.23 | -5.31 |
| I | AspG054427 | 0.00 | 0.00 | 0.00 | 2.86 |  | AspG043731 | 0.00 | 0.00 | 0.00 | 2.67 |
| I | AspG054459 | 0.00 | 0.00 | 2.02 | 3.30 |  | AspG009207 | 0.00 | 0.00 | 0.00 | 2.27 |
| I | AspG000690 | 0.00 | 0.00 | 0.00 | 3.15 |  | AspG021029 | 0.00 | 0.00 | 0.00 | 3.00 |
| I | AspG025530 | 0.00 | 0.00 | 0.00 | 2.85 |  | AspG025552 | 0.00 | 0.00 | 0.00 | 2.84 |
| I | AspG045852 | 0.00 | -2.16 | 0.00 | 0.00 |  | AspG005367 | 0.00 | -3.39 | 0.00 | 0.00 |
| I | AspG030259 | 0.00 | 0.00 | 0.00 | 3.02 |  | AspG005346 | 0.00 | 0.00 | 0.00 | 3.30 |
| I | AspG030270 | 2.69 | 0.00 | 0.00 | 6.19 |  | AspG005335 | 2.99 | 2.03 | 0.00 | 4.28 |
| I | AspG030282 | 3.48 | 0.00 | 0.00 | 0.00 |  | AspG005324 | 2.41 | 0.00 | 0.00 | 0.00 |
| I | AspG030298 | 0.00 | 0.00 | 0.00 | 4.12 |  | AspG005309 | 0.00 | 0.00 | 0.00 | 2.43 |
| I | AspG041559 | -4.89 | -3.80 | 0.00 | 0.00 |  | AspG005224 | -5.67 | -5.68 | 0.00 | 0.00 |
| I | AspG004979 | 0.00 | 3.14 | 0.00 | 0.00 |  | AspG005401 | 2.04 | 3.41 | 0.00 | 0.00 |
| I | AspG004979 | 0.00 | 3.14 | 0.00 | 0.00 |  | AspG005401 | 2.04 | 3.41 | 0.00 | 0.00 |
| I | AspG053779 | 0.00 | -2.51 | 0.00 | 0.00 |  | AspG036977 | 0.00 | -2.74 | 0.00 | 0.00 |
| I | AspG004240 | -2.91 | -2.03 | 0.00 | 0.00 |  | AspG010307 | -2.22 | 0.00 | 0.00 | 0.00 |
| I | AspG038038 | -2.11 | 0.00 | 0.00 | 0.00 |  | AspG019766 | -2.43 | 0.00 | 0.00 | 0.00 |
| I | AspG054046 | 3.85 | 0.00 | 0.00 | 0.00 |  | AspG046485 | 3.51 | 2.20 | 0.00 | 0.00 |
| I | AspG022896 | 3.08 | 0.00 | 0.00 | 0.00 |  | AspG053857 | 4.26 | 3.02 | 0.00 | 0.00 |
| I | AspG041310 | -2.57 | -2.52 | 0.00 | 0.00 |  | AspG056458 | -3.16 | -2.34 | 0.00 | 0.00 |
| I | AspG018947 | 0.00 | 0.00 | 2.03 | 3.11 |  | AspG016361 | 0.00 | 0.00 | 3.38 | 2.36 |
| I | AspG018941 | 4.10 | 4.00 | 0.00 | 0.00 |  | AspG016356 | 3.18 | 4.05 | 0.00 | 0.00 |
| I | AspG017050 | 0.00 | 0.00 | 0.00 | 2.49 |  | AspG011830 | 0.00 | 0.00 | 0.00 | 2.55 |
| I | AspG017031 | 5.11 | 6.82 | 0.00 | 0.00 |  | AspG011812 | 4.98 | 4.70 | 0.00 | 0.00 |
| I | AspG017028 | 0.00 | 0.00 | 0.00 | 2.72 |  | AspG011809 | 0.00 | 0.00 | 0.00 | 3.72 |
| I | AspG027164 | -3.54 | -2.54 | 0.00 | 0.00 |  | AspG011045 | -2.20 | -3.00 | 0.00 | 0.00 |
| I | AspG027237 | 3.78 | 3.63 | 0.00 | 0.00 |  | AspG009898 | 0.00 | 2.40 | 0.00 | 0.00 |
| I | AspG039963 | 0.00 | 0.00 | 0.00 | 2.07 |  | AspG016077 | 0.00 | 0.00 | 0.00 | 2.57 |
| I | AspG018558 | 0.00 | 4.31 | 0.00 | 0.00 |  | AspG053676 | 3.38 | 4.41 | 0.00 | 0.00 |
| I | AspG029501 | 0.00 | -2.33 | 0.00 | 0.00 |  | AspG051567 | 0.00 | -3.06 | 0.00 | 0.00 |
| I | AspG056299 | -6.74 | -6.39 | 0.00 | -2.80 |  | AspG001914 | -2.77 | -2.12 | -3.09 | -3.65 |
| I | AspG056312 | 0.00 | 0.00 | 0.00 | 4.28 |  | AspG031590 | 0.00 | 0.00 | 0.00 | 3.11 |
| I | AspG007636 | 3.67 | 0.00 | 0.00 | 0.00 |  | AspG031578 | 4.20 | 0.00 | 0.00 | 0.00 |
| I | AspG018212 | 0.00 | 0.00 | 0.00 | 2.24 |  | AspG031567 | 0.00 | 0.00 | 0.00 | 2.02 |
| I | AspG037483 | -3.05 | -3.14 | 0.00 | 0.00 |  | AspG031471 | -2.95 | -2.27 | 0.00 | 0.00 |
| I | AspG037457 | 0.00 | 0.00 | 0.00 | -2.58 |  | AspG031456 | 0.00 | 0.00 | 0.00 | -2.24 |
| I | AspG037473 | -2.12 | 0.00 | 0.00 | -2.13 |  | AspG031440 | -2.53 | 0.00 | 0.00 | -2.20 |
| I | AspG037633 | 0.00 | 0.00 | -2.57 | -2.72 |  | AspG031651 | 0.00 | 0.00 | -2.21 | 0.00 |
| I | AspG028031 | 0.00 | -3.08 | 0.00 | 0.00 |  | AspG044346 | 0.00 | -2.08 | 0.00 | 0.00 |
| I | AspG028027 | 0.00 | 2.23 | 0.00 | 0.00 |  | AspG044351 | 0.00 | 2.25 | 0.00 | 0.00 |
| I | AspG052762 | 2.78 | 0.00 | 0.00 | 5.49 |  | AspG028242 | 3.41 | 0.00 | 0.00 | 3.43 |
| I | AspG005931 | 0.00 | -3.11 | 0.00 | 0.00 |  | AspG011303 | -2.26 | -3.81 | 0.00 | 0.00 |
| I | AspG005961 | -4.08 | 0.00 | 0.00 | 0.00 |  | AspG011329 | -2.58 | 0.00 | 0.00 | 0.00 |
| I | AspG033623 | -4.10 | -3.65 | 0.00 | 0.00 |  | AspG006244 | -4.77 | -3.67 | 0.00 | 0.00 |
| I | AspG002779 | 2.88 | 0.00 | 0.00 | 0.00 |  | AspG027899 | 2.81 | 0.00 | 0.00 | 0.00 |
| I | AspG041277 | 3.82 | 3.10 | 0.00 | 0.00 |  | AspG049038 | 0.00 | 2.62 | 0.00 | 0.00 |
| I | AspG042490 | -4.61 | -4.28 | -2.65 | -3.12 |  | AspG003124 | -5.22 | -3.63 | -4.46 | -4.97 |
| I | AspG049652 | 0.00 | -2.89 | 0.00 | 0.00 |  | AspG038265 | 0.00 | -2.01 | 0.00 | 0.00 |
| I | AspG028964 | 0.00 | 0.00 | -2.06 | 0.00 |  | AspG016963 | 0.00 | 0.00 | -2.40 | -2.22 |
| I | AspG052623 | -2.18 | 0.00 | 0.00 | 0.00 |  | AspG006100 | -2.53 | 0.00 | 0.00 | 0.00 |
| I | AspG021967 | 0.00 | 2.25 | 0.00 | 0.00 |  | AspG028861 | 0.00 | 2.45 | 0.00 | 0.00 |
| I | AspG040467 | -2.24 | -4.10 | 0.00 | 0.00 |  | AspG001074 | 0.00 | -3.95 | 0.00 | 0.00 |
| I | AspG036901 | 0.00 | 0.00 | 0.00 | 3.29 |  | AspG006725 | 0.00 | 0.00 | 0.00 | 2.68 |
| I | AspG044289 | 2.24 | 2.75 | 0.00 | 0.00 |  | AspG047767 | 0.00 | 2.29 | 0.00 | 0.00 |
| I | AspG050836 | 0.00 | 0.00 | 0.00 | 2.75 |  | AspG005388 | 0.00 | 0.00 | 0.00 | 2.79 |
| I | AspG024699 | 2.49 | 0.00 | 0.00 | 0.00 |  | AspG056947 | 2.17 | 0.00 | 0.00 | 0.00 |
| I | AspG055764 | 0.00 | 2.17 | 0.00 | 0.00 |  | AspG030802 | 2.91 | 3.73 | 0.00 | 0.00 |
| I | AspG055825 | 0.00 | 0.00 | 0.00 | 2.25 |  | AspG047703 | 0.00 | 0.00 | 0.00 | 2.05 |
| I | AspG055748 | 0.00 | 0.00 | 0.00 | 3.47 |  | AspG004574 | 0.00 | 0.00 | 0.00 | 3.00 |
| I | AspG006978 | -4.15 | 0.00 | 0.00 | 0.00 |  | AspG038491 | -3.48 | 0.00 | 0.00 | 0.00 |
| I | AspG006956 | 0.00 | 0.00 | 0.00 | 3.46 |  | AspG057552 | 0.00 | 0.00 | 0.00 | 2.60 |
| I | AspG003897 | 0.00 | 0.00 | 2.59 | 2.71 |  | AspG024694 | 0.00 | 0.00 | 3.16 | 3.83 |
| I | AspG006446 | 2.45 | 3.04 | 0.00 | 0.00 |  | AspG053973 | 3.91 | 0.00 | 0.00 | 0.00 |
| I | AspG006473 | 0.00 | 0.00 | 0.00 | 3.37 |  | AspG025354 | 0.00 | 0.00 | 0.00 | 2.24 |
| I | AspG000138 | 2.11 | 0.00 | 0.00 | 0.00 |  | AspG033273 | 2.58 | 0.00 | 0.00 | 0.00 |
| I | AspG045084 | 0.00 | 0.00 | 0.00 | 3.04 |  | AspG030317 | 0.00 | 0.00 | 0.00 | 3.44 |
| I | AspG039192 | 0.00 | -2.46 | 0.00 | 0.00 |  | AspG008084 | -2.05 | -2.85 | 0.00 | 0.00 |
| I | AspG029698 | 0.00 | 0.00 | 0.00 | 7.22 |  | AspG000531 | 0.00 | 0.00 | 0.00 | 4.04 |
| I | AspG000667 | 0.00 | 2.96 | 0.00 | 3.27 |  | AspG000545 | 2.29 | 4.13 | 0.00 | 2.28 |
| I | AspG026343 | -5.95 | -4.18 | -7.21 | -5.89 |  | AspG022418 | -7.17 | -7.18 | -7.69 | 0.00 |
| I | AspG026343 | -5.95 | -4.18 | -7.21 | -5.89 |  | AspG022418 | -7.17 | -7.18 | -7.69 | 0.00 |
| I | AspG045084 | 0.00 | 0.00 | 0.00 | 3.04 |  | AspG030317 | 0.00 | 0.00 | 0.00 | 3.44 |
| I | AspG039192 | 0.00 | -2.46 | 0.00 | 0.00 |  | AspG008084 | -2.05 | -2.85 | 0.00 | 0.00 |
| I | AspG039188 | 0.00 | 0.00 | 4.30 | 2.03 |  | AspG003578 | 0.00 | 0.00 | 3.17 | 2.06 |
| I | AspG013700 | 0.00 | 0.00 | 0.00 | 2.21 |  | AspG002367 | 0.00 | 0.00 | 0.00 | 2.01 |
| I | AspG053295 | 4.19 | 0.00 | 0.00 | 2.30 |  | AspG049693 | 3.18 | 0.00 | 0.00 | 5.61 |
| I | AspG028834 | 0.00 | 0.00 | 2.01 | 2.79 |  | AspG056584 | 0.00 | 0.00 | 0.00 | 3.50 |
| I | AspG044909 | -2.63 | -4.04 | 0.00 | 0.00 |  | AspG000363 | -3.29 | -4.08 | 0.00 | 0.00 |
| I | AspG044434 | -2.76 | 0.00 | 0.00 | 0.00 |  | AspG032693 | -3.70 | -2.96 | 0.00 | 0.00 |
| I | AspG048216 | 0.00 | 0.00 | 0.00 | 5.17 |  | AspG048063 | 0.00 | 0.00 | 0.00 | 7.00 |
| I | AspG024930 | 0.00 | 0.00 | 0.00 | 2.34 |  | AspG025721 | 0.00 | 0.00 | 0.00 | 3.59 |
| I | AspG053640 | -4.95 | -6.33 | 0.00 | 0.00 |  | AspG021443 | -2.64 | -3.13 | 0.00 | 0.00 |
| I | AspG047171 | 0.00 | 0.00 | 0.00 | 2.91 |  | AspG021509 | 0.00 | 0.00 | 0.00 | 4.14 |
| I | AspG003609 | -2.51 | 0.00 | 0.00 | 0.00 |  | AspG052680 | -5.63 | -4.09 | 0.00 | 0.00 |
| I | AspG005095 | -3.76 | -3.29 | 0.00 | 0.00 |  | AspG020073 | -3.07 | 0.00 | 0.00 | 0.00 |
| I | AspG053314 | -2.22 | 0.00 | 0.00 | 0.00 |  | AspG020002 | -3.47 | 0.00 | 0.00 | 0.00 |
| I | AspG057270 | 0.00 | 0.00 | 0.00 | 2.17 |  | AspG018750 | 0.00 | 0.00 | 0.00 | 2.14 |
| I | AspG031972 | -2.55 | -3.48 | 0.00 | -4.81 |  | AspG025458 | 0.00 | -2.49 | -2.24 | -4.43 |
| II (108) | AspG046643 | 2.21 | 0.00 | 0.00 | 2.38 |  | AspG008887 | 0.00 | 0.00 | 0.00 | 2.44 |
| II | AspG028181 | 0.00 | 0.00 | 0.00 | 2.54 |  | AspG043266 | 2.21 | 0.00 | 0.00 | 0.00 |
| II | AspG024900 | 0.00 | 0.00 | 0.00 | 2.93 |  | AspG025730 | 4.63 | 2.35 | 0.00 | 3.67 |
| II | AspG024929 | 2.72 | 2.19 | 0.00 | 3.76 |  | AspG047931 | 3.27 | 3.21 | 0.00 | 0.00 |
| II | AspG014174 | -2.30 | -2.10 | 0.00 | 0.00 |  | AspG052825 | 0.00 | 0.00 | -2.48 | 0.00 |
| II | AspG014132 | -3.66 | -5.23 | 0.00 | -2.73 |  | AspG050265 | 0.00 | -3.01 | 0.00 | 0.00 |
| II | AspG023844 | 3.19 | 2.22 | 2.32 | 0.00 |  | AspG028132 | 0.00 | 0.00 | 0.00 | 2.46 |
| II | AspG023999 | 0.00 | 2.94 | 0.00 | 0.00 |  | AspG050397 | 2.11 | 2.39 | 0.00 | 4.32 |
| II | AspG028473 | 3.43 | 2.07 | 4.76 | 8.14 |  | AspG051713 | 0.00 | 0.00 | 0.00 | 5.23 |
| II | AspG028808 | 2.00 | 0.00 | 0.00 | 2.93 |  | AspG035755 | 0.00 | 0.00 | 0.00 | 2.17 |
| II | AspG029275 | 0.00 | -2.48 | 0.00 | 0.00 |  | AspG025328 | -2.39 | -3.72 | 0.00 | -2.83 |
| II | AspG004030 | 0.00 | 0.00 | 0.00 | 2.75 |  | AspG035195 | 2.00 | 0.00 | 0.00 | 0.00 |
| II | AspG017525 | -2.67 | 0.00 | 0.00 | -2.15 |  | AspG019923 | 0.00 | 0.00 | 0.00 | -2.15 |
| II | AspG034469 | 4.07 | 0.00 | 0.00 | 0.00 |  | AspG052078 | 0.00 | 0.00 | 0.00 | 3.05 |
| II | AspG017175 | -2.80 | 0.00 | 0.00 | 0.00 |  | AspG038198 | 0.00 | 0.00 | 0.00 | -2.05 |
| II | AspG020486 | 0.00 | 0.00 | 0.00 | -2.79 |  | AspG020882 | 0.00 | -2.95 | 0.00 | 0.00 |
| II | AspG049751 | 0.00 | 0.00 | 0.00 | 3.61 |  | AspG041631 | 2.09 | 0.00 | 2.92 | 4.09 |
| II | AspG049807 | 3.08 | 0.00 | 3.16 | 3.94 |  | AspG042764 | 2.49 | 0.00 | 0.00 | 0.00 |
| II | AspG057274 | 0.00 | 2.23 | 0.00 | 2.36 |  | AspG042789 | 0.00 | 0.00 | 0.00 | 2.04 |
| II | AspG049863 | 2.18 | 0.00 | 0.00 | 5.12 |  | AspG042868 | 0.00 | 0.00 | 0.00 | 6.06 |
| II | AspG025787 | 3.01 | 2.06 | 0.00 | 3.67 |  | AspG042892 | 2.48 | 0.00 | 0.00 | 0.00 |
| II | AspG050446 | -2.72 | -3.17 | 0.00 | 0.00 |  | AspG048770 | -3.52 | -3.83 | 0.00 | -2.37 |
| II | AspG040160 | 0.00 | 0.00 | 0.00 | -3.35 |  | AspG050199 | -2.80 | -2.43 | 0.00 | -2.77 |
| II | AspG040153 | -2.58 | 0.00 | -2.16 | -2.36 |  | AspG050195 | 0.00 | -2.15 | 0.00 | 0.00 |
| II | AspG003975 | 3.14 | 0.00 | 0.00 | 4.74 |  | AspG051948 | 0.00 | 0.00 | 0.00 | 2.81 |
| II | AspG000354 | -2.66 | -2.16 | -2.50 | -2.32 |  | AspG053419 | -2.76 | -2.81 | 0.00 | 0.00 |
| II | AspG000339 | -2.84 | -2.67 | 0.00 | 0.00 |  | AspG053438 | 0.00 | -2.36 | -2.18 | -2.10 |
| II | AspG006682 | 0.00 | 0.00 | -2.97 | -2.79 |  | AspG011565 | -2.41 | 0.00 | -3.16 | -3.06 |
| II | AspG044656 | 0.00 | 0.00 | 0.00 | 2.08 |  | AspG014367 | 2.15 | 0.00 | 0.00 | 0.00 |
| II | AspG046114 | 2.12 | 0.00 | 0.00 | 0.00 |  | AspG022196 | 6.01 | 0.00 | 0.00 | 6.19 |
| II | AspG049390 | -4.05 | -4.96 | 0.00 | -6.41 |  | AspG002051 | -6.31 | -7.52 | 0.00 | 0.00 |
| II | AspG045254 | -2.72 | -2.77 | 0.00 | -2.32 |  | AspG025672 | -2.43 | -2.25 | 0.00 | 0.00 |
| II | AspG052242 | 6.09 | 3.10 | 0.00 | 0.00 |  | AspG021159 | 6.90 | 6.85 | 4.78 | 4.41 |
| II | AspG002069 | 0.00 | 0.00 | 0.00 | 2.75 |  | AspG036376 | 2.69 | 0.00 | 0.00 | 0.00 |
| II | AspG015549 | 4.33 | 3.19 | 0.00 | 4.25 |  | AspG035244 | 3.00 | 0.00 | 0.00 | 0.00 |
| II | AspG029568 | -3.15 | -3.25 | 0.00 | -2.14 |  | AspG025441 | 0.00 | -2.39 | 0.00 | 0.00 |
| II | AspG049744 | 0.00 | 0.00 | 2.64 | 0.00 |  | AspG007936 | 2.44 | 0.00 | 0.00 | 0.00 |
| II | AspG051364 | 0.00 | 0.00 | 0.00 | 3.61 |  | AspG034263 | 2.51 | 3.77 | 0.00 | 5.39 |
| II | AspG051795 | -2.38 | 0.00 | 0.00 | 0.00 |  | AspG008679 | 0.00 | -2.08 | 0.00 | -2.20 |
| II | AspG051913 | 0.00 | 0.00 | 0.00 | -3.29 |  | AspG008704 | -2.44 | -2.26 | 0.00 | 0.00 |
| II | AspG021612 | 3.77 | 0.00 | 2.79 | 0.00 |  | AspG056615 | 0.00 | 0.00 | 0.00 | 3.18 |
| II | AspG021595 | -3.26 | -3.32 | 0.00 | 0.00 |  | AspG056732 | 0.00 | 0.00 | 0.00 | -2.13 |
| II | AspG013298 | 0.00 | 0.00 | 0.00 | 3.81 |  | AspG042885 | 2.58 | 0.00 | 0.00 | 5.87 |
| II | AspG013277 | 0.00 | 0.00 | 0.00 | 2.11 |  | AspG042753 | 3.71 | 2.71 | 0.00 | 0.00 |
| II | AspG032374 | -2.32 | 0.00 | 0.00 | 0.00 |  | AspG024504 | -3.85 | -2.30 | 0.00 | -3.98 |
| II | AspG032441 | 0.00 | 0.00 | 0.00 | 2.02 |  | AspG013902 | 2.96 | 0.00 | 0.00 | 0.00 |
| II | AspG001675 | -3.33 | -5.70 | 0.00 | -2.43 |  | AspG007237 | -3.13 | -4.03 | 0.00 | 0.00 |
| II | AspG055938 | 3.02 | 0.00 | 0.00 | 0.00 |  | AspG013981 | 2.15 | 0.00 | 0.00 | 2.26 |
| II | AspG015897 | -5.00 | -2.96 | -3.16 | 0.00 |  | AspG048040 | -7.38 | -5.35 | 0.00 | 0.00 |
| II | AspG051696 | 3.83 | 4.74 | 2.00 | 2.91 |  | AspG036445 | 5.11 | 5.32 | 0.00 | 0.00 |
| II | AspG005175 | -3.27 | -3.09 | 0.00 | 0.00 |  | AspG000664 | -2.62 | -2.17 | 0.00 | -2.55 |
| II | AspG029646 | 0.00 | 0.00 | 0.00 | 2.60 |  | AspG045353 | 2.04 | 0.00 | 2.16 | 3.58 |
| II | AspG022587 | -2.47 | 0.00 | 0.00 | -2.21 |  | AspG055540 | -2.09 | 0.00 | 0.00 | 0.00 |
| II | AspG048141 | 0.00 | 0.00 | 0.00 | 4.74 |  | AspG007131 | 3.92 | 6.21 | 0.00 | 0.00 |
| II | AspG051297 | 0.00 | 2.40 | 0.00 | 0.00 |  | AspG045806 | 2.76 | 0.00 | 0.00 | 2.43 |
| II | AspG032237 | 0.00 | 2.27 | 3.15 | 4.00 |  | AspG056872 | 4.61 | 3.87 | 0.00 | 0.00 |
| II | AspG022158 | 0.00 | 2.33 | 2.48 | 3.38 |  | AspG017777 | 0.00 | 0.00 | 3.53 | 2.17 |
| II | AspG039648 | 0.00 | 0.00 | 0.00 | -2.60 |  | AspG017804 | 0.00 | -2.66 | 0.00 | -3.05 |
| II | AspG039663 | -2.24 | 0.00 | 0.00 | -2.24 |  | AspG017828 | -4.61 | -4.87 | 0.00 | 0.00 |
| II | AspG044562 | 0.00 | 0.00 | 0.00 | 4.32 |  | AspG041723 | 2.28 | 0.00 | 0.00 | 0.00 |
| II | AspG049273 | 0.00 | 0.00 | 0.00 | -3.10 |  | AspG028565 | -2.35 | -3.23 | -2.71 | -3.00 |
| II | AspG042655 | 2.13 | 0.00 | 0.00 | 2.66 |  | AspG015258 | 0.00 | 0.00 | 0.00 | 2.39 |
| II | AspG042679 | -2.61 | -2.14 | 0.00 | 0.00 |  | AspG033002 | 0.00 | 0.00 | 0.00 | -2.12 |
| II | AspG016850 | 2.09 | 0.00 | 0.00 | 0.00 |  | AspG016483 | 0.00 | 0.00 | 0.00 | 2.47 |
| II | AspG001255 | 0.00 | 0.00 | -2.17 | 0.00 |  | AspG053212 | -3.07 | -3.07 | 0.00 | 0.00 |
| II | AspG011022 | -4.23 | -2.25 | -2.53 | -2.69 |  | AspG031780 | 0.00 | 0.00 | 0.00 | -2.65 |
| II | AspG001565 | 0.00 | -2.19 | 0.00 | 0.00 |  | AspG001139 | -2.92 | -3.66 | 0.00 | -2.03 |
| II | AspG055091 | -2.38 | 0.00 | 0.00 | 0.00 |  | AspG049992 | -3.34 | 0.00 | -2.57 | 0.00 |
| II | AspG026041 | 3.28 | 3.04 | 2.78 | 2.96 |  | AspG001024 | 3.80 | 3.83 | 0.00 | 0.00 |
| II | AspG019231 | 0.00 | -2.06 | 0.00 | 0.00 |  | AspG035144 | 0.00 | 0.00 | 0.00 | -2.22 |
| II | AspG054000 | -3.59 | -3.36 | 0.00 | -2.59 |  | AspG028759 | -2.11 | 0.00 | 0.00 | 0.00 |
| II | AspG029133 | 7.36 | 0.00 | 2.60 | 3.99 |  | AspG046119 | 0.00 | 0.00 | 0.00 | 4.09 |
| II | AspG017361 | 2.43 | 2.00 | 0.00 | 0.00 |  | AspG035650 | 0.00 | 0.00 | 0.00 | 2.34 |
| II | AspG028578 | -5.77 | -4.68 | 0.00 | 0.00 |  | AspG048285 | -6.17 | -5.46 | -2.02 | 0.00 |
| II | AspG039310 | -4.70 | -3.61 | 0.00 | 0.00 |  | AspG048295 | -5.14 | -6.46 | 0.00 | -2.27 |
| II | AspG039318 | -2.91 | 0.00 | 0.00 | -3.04 |  | AspG034361 | 0.00 | 0.00 | 0.00 | -3.52 |
| II | AspG039336 | 3.24 | 3.12 | 0.00 | 5.63 |  | AspG054093 | 0.00 | 0.00 | 2.36 | 4.32 |
| II | AspG021932 | 2.10 | 0.00 | 0.00 | 2.66 |  | AspG014792 | 0.00 | 0.00 | 0.00 | 3.77 |
| II | AspG002199 | 3.65 | 2.14 | 2.33 | 0.00 |  | AspG021050 | 2.11 | 0.00 | 0.00 | 0.00 |
| II | AspG020916 | 2.02 | 0.00 | 0.00 | 2.77 |  | AspG006170 | 0.00 | 0.00 | 0.00 | 4.08 |
| II | AspG001761 | -2.35 | -2.83 | 0.00 | 0.00 |  | AspG027635 | -5.15 | -6.60 | -2.58 | -4.17 |
| II | AspG030286 | -6.30 | -4.66 | 0.00 | 0.00 |  | AspG005320 | -3.70 | -3.43 | 0.00 | -2.34 |
| II | AspG041551 | 3.20 | 0.00 | 0.00 | 2.85 |  | AspG005232 | 2.19 | 0.00 | 0.00 | 0.00 |
| II | AspG041417 | 0.00 | -2.56 | 0.00 | 0.00 |  | AspG005187 | 0.00 | 0.00 | 0.00 | -2.41 |
| II | AspG041972 | -2.95 | 0.00 | -3.40 | -4.43 |  | AspG004404 | -3.80 | 0.00 | 0.00 | 0.00 |
| II | AspG008600 | -2.98 | -2.75 | 0.00 | -2.93 |  | AspG046507 | 0.00 | 0.00 | 0.00 | -3.39 |
| II | AspG055723 | 2.81 | 0.00 | 0.00 | 0.00 |  | AspG053948 | 0.00 | 0.00 | 0.00 | 5.67 |
| II | AspG022887 | 0.00 | 0.00 | -2.44 | -2.75 |  | AspG053848 | 0.00 | -2.01 | 0.00 | -2.10 |
| II | AspG029489 | 2.58 | 0.00 | 0.00 | 0.00 |  | AspG051550 | 0.00 | 0.00 | 0.00 | 3.04 |
| II | AspG037391 | 2.47 | 2.19 | 2.78 | 4.41 |  | AspG031361 | 2.68 | 0.00 | 0.00 | 0.00 |
| II | AspG037611 | -5.97 | -3.26 | 0.00 | 0.00 |  | AspG031623 | -8.52 | -5.92 | 0.00 | -2.10 |
| II | AspG055471 | 0.00 | 0.00 | 0.00 | 5.17 |  | AspG051466 | 2.51 | 0.00 | 0.00 | 0.00 |
| II | AspG046651 | 4.50 | 5.21 | 3.62 | 7.17 |  | AspG055148 | 0.00 | 0.00 | 2.92 | 6.58 |
| II | AspG002817 | 2.04 | 0.00 | 0.00 | 0.00 |  | AspG041805 | 0.00 | 0.00 | 0.00 | 2.67 |
| II | AspG020291 | -3.22 | -4.21 | 0.00 | 0.00 |  | AspG038985 | -6.59 | -6.18 | 0.00 | -2.27 |
| II | AspG028818 | -2.06 | 0.00 | 0.00 | 0.00 |  | AspG027473 | -2.47 | 0.00 | 0.00 | -2.30 |
| II | AspG020856 | 2.82 | 0.00 | 0.00 | 3.37 |  | AspG028084 | 0.00 | 0.00 | 0.00 | 2.89 |
| II | AspG056196 | 2.72 | 0.00 | 0.00 | 6.78 |  | AspG002258 | 0.00 | 0.00 | 0.00 | 2.73 |
| II | AspG056197 | 4.37 | 4.62 | 0.00 | 7.56 |  | AspG002258 | 0.00 | 0.00 | 0.00 | 2.73 |
| II | AspG044315 | 0.00 | 0.00 | -2.18 | -2.29 |  | AspG046375 | -3.09 | 0.00 | -2.16 | -2.02 |
| II | AspG014759 | 0.00 | 0.00 | 0.00 | 2.26 |  | AspG019418 | 0.00 | 2.01 | 0.00 | 0.00 |
| II | AspG008529 | 3.82 | 2.18 | 0.00 | 0.00 |  | AspG037184 | 0.00 | 0.00 | 0.00 | 2.25 |
| II | AspG055850 | 0.00 | 0.00 | -2.78 | -2.46 |  | AspG047684 | -2.19 | 0.00 | -2.62 | -3.78 |
| II | AspG032648 | 0.00 | 0.00 | 0.00 | -2.61 |  | AspG025285 | -2.79 | -3.36 | -2.48 | -3.34 |
| II | AspG035053 | 0.00 | 0.00 | 0.00 | 2.20 |  | AspG017086 | 3.21 | 2.04 | 0.00 | 0.00 |
| II | AspG004173 | -4.63 | -4.51 | -3.84 | -5.03 |  | AspG044603 | 0.00 | 0.00 | 0.00 | -3.94 |
| II | AspG031927 | -3.36 | -2.56 | 0.00 | -2.53 |  | AspG002187 | 0.00 | 0.00 | 0.00 | -2.46 |
| II | AspG003417 | 0.00 | 0.00 | 0.00 | 2.60 |  | AspG011954 | 0.00 | 2.08 | 0.00 | 0.00 |
| III (10) | AspG016692 | 0.00 | -2.68 | 0.00 | 0.00 |  | AspG016471 | 2.21 | -2.24 | 0.00 | 0.00 |
| III | AspG040100 | 0.00 | 0.00 | 0.00 | -3.95 |  | AspG021313 | 0.00 | 0.00 | 0.00 | 3.15 |
| III | AspG003958 | -3.20 | -6.20 | 0.00 | 0.00 |  | AspG047438 | 2.37 | 0.00 | 0.00 | 0.00 |
| III | AspG050581 | 2.96 | 0.00 | 0.00 | 0.00 |  | AspG008757 | 0.00 | -2.04 | 0.00 | 0.00 |
| III | AspG013088 | -2.14 | 0.00 | 0.00 | 0.00 |  | AspG014007 | 2.01 | 2.24 | 0.00 | 0.00 |
| III | AspG039619 | 0.00 | 2.13 | 0.00 | 0.00 |  | AspG033554 | -2.32 | 0.00 | 0.00 | 0.00 |
| III | AspG034375 | -2.17 | -2.04 | 0.00 | 0.00 |  | AspG056453 | 2.08 | 0.00 | 0.00 | 0.00 |
| III | AspG037494 | -2.74 | -2.28 | 0.00 | 0.00 |  | AspG031484 | 2.74 | 0.00 | 0.00 | 0.00 |
| III | AspG006872 | 2.04 | 0.00 | 0.00 | 0.00 |  | AspG033302 | 0.00 | -2.92 | 0.00 | 0.00 |
| III | AspG031966 | 2.90 | 0.00 | 0.00 | 0.00 |  | AspG049684 | 0.00 | -2.92 | 0.00 | 0.00 |
| IV (26) | AspG043889 | 2.13 | 2.76 | 0.00 | 0.00 |  | AspG006509 | 2.01 | 3.14 | -3.67 | -6.16 |
| IV | AspG027416 | 0.00 | 0.00 | 0.00 | 2.08 |  | AspG033778 | 0.00 | -2.05 | 0.00 | 2.06 |
| IV | AspG043424 | -2.40 | 0.00 | 0.00 | 3.90 |  | AspG054093 | 0.00 | 0.00 | 2.36 | 4.32 |
| IV | AspG040108 | 0.00 | 2.40 | 0.00 | 0.00 |  | AspG021321 | 3.96 | 3.95 | 0.00 | -3.82 |
| IV | AspG046217 | -2.11 | -2.45 | 0.00 | 2.20 |  | AspG021331 | -2.01 | 0.00 | 0.00 | 0.00 |
| IV | AspG024333 | 0.00 | 0.00 | 0.00 | -2.17 |  | AspG045009 | 0.00 | 2.31 | 0.00 | 0.00 |
| IV | AspG052015 | 2.12 | 0.00 | 0.00 | 0.00 |  | AspG036348 | 0.00 | 0.00 | -2.26 | -3.28 |
| IV | AspG023137 | 0.00 | 0.00 | 0.00 | 5.03 |  | AspG034334 | -2.16 | -2.80 | 0.00 | 4.77 |
| IV | AspG048149 | 0.00 | 0.00 | 0.00 | 3.77 |  | AspG008813 | -2.63 | 0.00 | 0.00 | 0.00 |
| IV | AspG056143 | -4.41 | -4.05 | 0.00 | 0.00 |  | AspG051615 | 0.00 | 0.00 | 0.00 | 2.02 |
| IV | AspG056139 | 0.00 | -2.57 | 0.00 | 0.00 |  | AspG051622 | 0.00 | 0.00 | 0.00 | 3.65 |
| IV | AspG034119 | -3.95 | -7.70 | 0.00 | 9.63 |  | AspG028291 | 0.00 | 0.00 | 0.00 | 6.63 |
| IV | AspG032292 | -4.81 | -3.99 | 0.00 | 0.00 |  | AspG021828 | 0.00 | 0.00 | 0.00 | 2.15 |
| IV | AspG008959 | 3.59 | 4.30 | 0.00 | 0.00 |  | AspG000660 | 2.42 | 0.00 | 0.00 | -3.73 |
| IV | AspG051406 | 0.00 | 0.00 | 0.00 | 2.65 |  | AspG034018 | -2.97 | -3.21 | 0.00 | 0.00 |
| IV | AspG048103 | 0.00 | -2.73 | 0.00 | 0.00 |  | AspG005071 | 0.00 | 0.00 | 0.00 | 4.24 |
| IV | AspG039345 | 0.00 | -3.09 | 0.00 | 0.00 |  | AspG034370 | 0.00 | 0.00 | 0.00 | 2.53 |
| IV | AspG021934 | 2.54 | 2.62 | 0.00 | 0.00 |  | AspG014788 | 0.00 | 0.00 | 0.00 | -3.01 |
| IV | AspG017836 | 0.00 | 0.00 | 0.00 | 2.06 |  | AspG028985 | 0.00 | -3.49 | 0.00 | 2.13 |
| IV | AspG018066 | -2.48 | 0.00 | 0.00 | 0.00 |  | AspG039899 | -2.19 | 0.00 | 0.00 | 4.03 |
| IV | AspG028119 | 0.00 | 0.00 | 0.00 | 2.60 |  | AspG049989 | 0.00 | -2.03 | 0.00 | 0.00 |
| IV | AspG050790 | 0.00 | 0.00 | 0.00 | 2.08 |  | AspG018409 | 0.00 | -2.14 | 0.00 | 0.00 |
| IV | AspG017043 | 3.63 | 0.00 | 0.00 | 0.00 |  | AspG011821 | 0.00 | 0.00 | 0.00 | -2.05 |
| IV | AspG008954 | 0.00 | -2.33 | 0.00 | 0.00 |  | AspG033017 | 3.14 | 3.04 | 2.58 | 4.02 |
| IV | AspG056307 | 0.00 | 0.00 | 0.00 | -4.84 |  | AspG031595 | 2.34 | 2.64 | 0.00 | 0.00 |
| IV | AspG037383 | 0.00 | 0.00 | -2.87 | 0.00 |  | AspG000658 | 2.85 | 0.00 | 0.00 | 0.00 |

**Table S21** The expression (TPM) of the salt-tolerance related DEGs in root and shoot of *A. splendens*. The degree of regulation of the genes in red color at 6 h in root and at 24 h in shoot are represented in Figure 5c.

| Gene family | Gene ID | CK_Root | 6h_Root | 24h_Root | CK_Shoot | 6h_Shoot | 24h_Shoot |
| --- | --- | --- | --- | --- | --- | --- | --- |
| SOS1 | AspG038708 | 177.9026 | 245.2689 | 270.4106 | 39.8170 | 65.0561 | 86.9525 |
| SOS2 | AspG030726 | 27.8656 | 38.2709 | 40.9397 | 20.2825 | 24.1108 | 29.3781 |
| SOS3 | AspG015733 | 7.9670 | 13.9415 | 8.7559 | 0.0000 | 0.0000 | 0.0000 |
| AHA | AspG040496 | 4.4096 | 1.1282 | 0.8569 | 1.2202 | 1.7123 | 1.0211 |
|  | AspG045818 | 20.5902 | 167.3596 | 136.6814 | 13.2448 | 52.6416 | 253.3554 |
|  | AspG013514 | 49.1393 | 216.5512 | 203.3676 | 29.0966 | 112.0624 | 144.6530 |
| HKT1 | AspG013501 | 0.0709 | 0.0000 | 0.0363 | 0.8173 | 2.6846 | 6.0046 |
| GLR | AspG056575 | 0.0263 | 0.8627 | 0.3404 | 0.2771 | 2.0550 | 3.5152 |
| CNGC | AspG050460 | 0.0122 | 0.1857 | 0.1103 | 3.6214 | 6.4763 | 10.3606 |
| CIPK | AspG052916 | 0.2265 | 0.0882 | 0.1356 | 14.5311 | 6.3233 | 2.1520 |
|  | AspG019259 | 0.2337 | 0.0873 | 0.1487 | 17.1831 | 7.4132 | 2.4024 |
|  | AspG051708 | 1.6805 | 2.9881 | 2.3996 | 3.1736 | 6.3331 | 17.5272 |
|  | AspG011165 | 2.4165 | 3.8304 | 3.6075 | 4.0257 | 7.9633 | 23.5108 |
|  | AspG031344 | 10.0961 | 18.2663 | 39.0791 | 8.6656 | 11.5370 | 15.4528 |
|  | AspG046602 | 13.4650 | 57.5473 | 30.4622 | 73.4667 | 115.7791 | 191.1508 |
|  | AspG004240 | 13.6016 | 90.4700 | 51.8021 | 8.1063 | 25.7457 | 18.6318 |
|  | AspG010307 | 19.6975 | 85.2845 | 52.5785 | 7.3268 | 19.9228 | 22.7745 |
|  | AspG016963 | 26.9530 | 25.0938 | 29.0697 | 0.8111 | 4.2051 | 3.4344 |
|  | AspG028964 | 88.9211 | 79.4661 | 46.1412 | 0.9655 | 4.3799 | 3.3452 |
|  | AspG013308 | 139.0483 | 82.3092 | 120.0083 | 45.4147 | 96.5907 | 210.2449 |
|  | AspG000653 | 336.2046 | 103.8075 | 57.6881 | 55.9312 | 22.2564 | 11.1003 |
| NHX | AspG037444 | 1.3813 | 22.3586 | 12.8170 | 7.0173 | 17.4970 | 23.5234 |
|  | AspG007608 | 3.0716 | 13.0109 | 7.1536 | 4.4031 | 5.7642 | 5.6698 |
|  | AspG031421 | 9.1399 | 36.4530 | 34.5895 | 48.1830 | 106.4699 | 112.7214 |
| ACA | AspG040496 | 6.6060 | 1.4899 | 1.2608 | 1.9348 | 2.5112 | 1.0211 |
|  | AspG031951 | 10.0335 | 40.6168 | 22.7544 | 53.8205 | 32.4120 | 13.4008 |
|  | AspG013514 | 63.1645 | 281.1262 | 257.4732 | 39.0774 | 149.4418 | 144.6530 |
| bZIP | AspG018068 | 0.9651 | 6.8387 | 4.5470 | 2.2649 | 4.8578 | 5.3006 |
|  | AspG012547 | 1.1411 | 3.2724 | 5.7638 | 2.9960 | 4.5627 | 7.1723 |
|  | AspG041818 | 1.5152 | 5.9072 | 3.8114 | 1.0677 | 3.6238 | 6.0835 |
|  | AspG034729 | 1.7560 | 17.3814 | 9.2336 | 0.9967 | 10.2643 | 15.4970 |
|  | AspG018449 | 3.1727 | 9.2825 | 7.2979 | 12.5994 | 29.2722 | 35.4751 |
|  | AspG055402 | 3.8246 | 18.4034 | 14.6507 | 4.4016 | 15.2350 | 15.8720 |
|  | AspG018057 | 5.5413 | 36.7410 | 14.9654 | 1.6454 | 7.2265 | 4.8578 |
|  | AspG033875 | 5.6251 | 39.8101 | 23.1992 | 2.5932 | 7.1614 | 6.2011 |
|  | AspG003380 | 6.0697 | 0.8961 | 1.2314 | 5.4292 | 1.7617 | 0.1115 |
|  | AspG004335 | 6.1161 | 16.2564 | 37.9004 | 9.8695 | 13.6133 | 13.6547 |
|  | AspG000682 | 8.3463 | 1.7088 | 2.3606 | 1.0307 | 0.6266 | 0.1544 |
|  | AspG000771 | 9.4933 | 3.9413 | 5.0429 | 3.7586 | 1.3383 | 0.0472 |
|  | AspG027652 | 10.0375 | 0.2650 | 0.5916 | 3.9175 | 2.5651 | 5.1465 |
|  | AspG042868 | 14.7819 | 4.9908 | 4.2235 | 3.4716 | 1.2812 | 0.0653 |
|  | AspG006723 | 26.2617 | 2.2771 | 2.2564 | 0.1993 | 0.2607 | 0.3601 |
|  | AspG049863 | 28.9377 | 5.4416 | 8.3090 | 4.2364 | 1.5095 | 0.1002 |
|  | AspG022365 | 29.4208 | 136.4484 | 77.2686 | 23.3571 | 69.2610 | 54.4893 |
|  | AspG005401 | 69.0392 | 14.8692 | 6.1314 | 0.4183 | 0.0839 | 0.3181 |
|  | AspG053478 | 70.7661 | 83.0603 | 80.0026 | 2.5184 | 8.7139 | 11.9721 |
|  | AspG004979 | 96.1703 | 50.5517 | 11.1205 | 0.3798 | 0.2122 | 0.1780 |
|  | AspG009884 | 116.5405 | 43.1374 | 24.2534 | 0.0214 | 0.0000 | 0.0620 |
| HB | AspG011534 | 1.0973 | 1.9018 | 5.1881 | 0.0926 | 0.2581 | 0.0372 |
|  | AspG043032 | 7.3624 | 1.6363 | 3.3225 | 10.2298 | 10.0040 | 8.9918 |
| bHLH | AspG008904 | 0.1959 | 1.0109 | 1.0294 | 5.9567 | 17.4152 | 48.6516 |
|  | AspG022572 | 0.5755 | 0.1012 | 0.6552 | 7.0748 | 7.7891 | 23.8456 |
|  | AspG022011 | 1.2805 | 52.9014 | 7.1922 | 1.9632 | 24.1082 | 6.6642 |
|  | AspG019063 | 1.5109 | 6.7186 | 3.6121 | 0.5130 | 5.0154 | 2.6403 |
|  | AspG045570 | 1.5603 | 6.9987 | 3.6380 | 15.5603 | 19.9674 | 15.0992 |
|  | AspG054393 | 1.7597 | 30.7339 | 20.3860 | 13.3104 | 28.8570 | 22.7611 |
|  | AspG028880 | 2.9977 | 1.3161 | 3.0529 | 1.7949 | 3.0753 | 11.3777 |
|  | AspG024280 | 3.9724 | 0.6194 | 1.7134 | 0.3703 | 0.4484 | 0.6096 |
|  | AspG049087 | 5.1905 | 1.0327 | 1.5841 | 0.4633 | 0.2532 | 0.0860 |
|  | AspG033608 | 5.7863 | 2.7407 | 1.4412 | 1.6561 | 0.7101 | 0.9098 |
|  | AspG002447 | 6.1663 | 4.7237 | 3.7764 | 2.2281 | 1.1572 | 0.0604 |
|  | AspG028703 | 6.4153 | 1.5230 | 2.7866 | 0.4997 | 0.6298 | 0.8577 |
|  | AspG015381 | 12.2926 | 2.8555 | 7.4737 | 12.5514 | 6.9922 | 1.6225 |
|  | AspG015448 | 13.5513 | 7.1425 | 5.8003 | 8.9757 | 8.2954 | 2.0315 |
|  | AspG056169 | 14.9566 | 6.8206 | 7.3038 | 1.9293 | 0.3408 | 0.0000 |
|  | AspG052856 | 18.8924 | 5.3543 | 3.4192 | 0.3630 | 0.4313 | 0.0458 |
|  | AspG050722 | 47.5804 | 9.4547 | 10.0676 | 0.7351 | 0.7771 | 0.3794 |
|  | AspG023576 | 56.3710 | 4.6658 | 4.5352 | 7.2071 | 3.2183 | 1.2036 |
|  | AspG054525 | 143.4193 | 18.7128 | 60.4621 | 19.4027 | 7.4416 | 9.9801 |
| WRKY | AspG004186 | 0.0223 | 0.5536 | 0.2437 | 6.1073 | 29.6688 | 15.9441 |
|  | AspG028660 | 0.8470 | 22.3603 | 10.3173 | 0.1308 | 0.0488 | 0.1430 |
|  | AspG008891 | 1.5314 | 3.7969 | 6.3577 | 3.1407 | 4.4239 | 7.0837 |
|  | AspG044484 | 2.9386 | 13.8444 | 6.9253 | 1.3747 | 3.9387 | 2.3228 |
|  | AspG033490 | 3.0912 | 2.7742 | 1.7389 | 1.6762 | 1.3226 | 0.3965 |
|  | AspG013374 | 3.2230 | 2.6024 | 1.9712 | 6.6852 | 6.2400 | 1.4182 |
|  | AspG007131 | 3.6626 | 0.2195 | 0.0638 | 0.1986 | 0.4868 | 0.0000 |
|  | AspG031332 | 5.4739 | 0.9465 | 0.4389 | 0.6869 | 1.0102 | 0.8461 |
|  | AspG016964 | 6.1530 | 1.4532 | 0.5688 | 10.0959 | 39.9235 | 19.3741 |
|  | AspG000637 | 8.3455 | 0.7903 | 1.0317 | 4.4775 | 5.2124 | 1.9196 |
|  | AspG028963 | 9.5892 | 6.3254 | 0.8573 | 20.2904 | 54.3006 | 17.1511 |
|  | AspG052868 | 35.0661 | 45.3651 | 23.1945 | 30.2414 | 40.5736 | 5.4107 |
|  | AspG052717 | 42.0988 | 22.4838 | 9.0515 | 8.6909 | 23.5856 | 5.3904 |
|  | AspG034655 | 57.9958 | 12.2224 | 7.3684 | 61.6177 | 112.1681 | 87.7358 |
|  | AspG028907 | 101.1524 | 25.3241 | 15.5909 | 53.1470 | 106.9510 | 47.5386 |
| NAC | AspG016809 | 0.2242 | 0.3110 | 0.0923 | 0.5747 | 3.2725 | 9.0719 |
|  | AspG044186 | 1.7123 | 0.3281 | 0.5779 | 6.5555 | 3.9664 | 2.3302 |
|  | AspG031656 | 3.0304 | 5.1204 | 4.7861 | 1.6490 | 4.9584 | 11.8629 |
|  | AspG039598 | 4.9338 | 10.4138 | 23.8969 | 22.2580 | 36.5337 | 27.6847 |
|  | AspG005020 | 5.1689 | 1.6139 | 0.8197 | 0.6148 | 0.5921 | 0.4441 |
|  | AspG055276 | 6.3462 | 14.9698 | 14.0889 | 0.2512 | 0.5199 | 1.9625 |
|  | AspG033103 | 6.4307 | 25.4819 | 11.3535 | 4.5349 | 13.5778 | 28.4666 |
|  | AspG012165 | 8.7413 | 26.9452 | 8.6023 | 3.3491 | 8.9204 | 15.5799 |
|  | AspG030307 | 9.0275 | 30.3873 | 28.1913 | 6.4553 | 22.0596 | 28.0691 |
|  | AspG005474 | 9.8093 | 7.5459 | 4.8764 | 14.1210 | 9.2826 | 3.1697 |
|  | AspG050876 | 13.5028 | 20.1664 | 15.9112 | 17.6875 | 54.6389 | 91.0256 |
|  | AspG015184 | 13.6136 | 57.6888 | 34.8485 | 3.0315 | 19.0651 | 9.9850 |
|  | AspG031651 | 17.7972 | 37.6056 | 23.4797 | 0.9736 | 4.3798 | 0.8436 |
|  | AspG037149 | 21.4470 | 36.0504 | 22.5290 | 6.1554 | 35.3618 | 22.0664 |
|  | AspG037633 | 23.2221 | 41.3138 | 22.8631 | 2.5543 | 15.6756 | 18.1460 |
| MYB | AspG000958 | 0.0000 | 0.0000 | 0.0000 | 2.7772 | 2.5005 | 0.4440 |
|  | AspG053640 | 0.0499 | 2.4015 | 6.5459 | 5.5572 | 10.7805 | 22.4629 |
|  | AspG021443 | 0.2095 | 1.6674 | 2.2547 | 8.4002 | 10.0938 | 16.4944 |
|  | AspG056306 | 0.2268 | 0.1877 | 0.9085 | 2.9226 | 8.9317 | 12.6008 |
|  | AspG001427 | 0.2677 | 2.2736 | 4.2924 | 4.9035 | 13.0946 | 10.3025 |
|  | AspG049709 | 0.4439 | 2.4129 | 1.3544 | 2.9706 | 5.4055 | 2.5194 |
|  | AspG055574 | 0.5159 | 2.0780 | 1.5007 | 2.7738 | 4.6864 | 2.5968 |
|  | AspG050154 | 0.5627 | 0.5809 | 1.1242 | 0.4274 | 1.5003 | 3.4689 |
|  | AspG014152 | 0.6567 | 1.2879 | 2.4858 | 0.3811 | 0.8838 | 2.3002 |
|  | AspG029354 | 0.7685 | 3.6287 | 2.4057 | 4.2981 | 8.0112 | 9.6751 |
|  | AspG012261 | 0.8108 | 7.9956 | 7.1422 | 21.4740 | 42.7976 | 52.1580 |
|  | AspG047245 | 0.8448 | 1.8777 | 4.8832 | 2.8740 | 4.3170 | 3.5088 |
|  | AspG019348 | 1.6886 | 8.9384 | 8.5097 | 3.7817 | 6.5663 | 10.3054 |
|  | AspG016843 | 1.7735 | 11.2623 | 6.9465 | 0.0000 | 0.1685 | 0.1177 |
|  | AspG040860 | 2.8343 | 15.5309 | 9.8196 | 0.9999 | 2.4223 | 3.7034 |
|  | AspG046922 | 2.9245 | 0.4092 | 0.2874 | 0.1889 | 0.0330 | 0.0000 |
|  | AspG020698 | 4.3624 | 5.9488 | 5.4100 | 0.5424 | 0.2925 | 0.0124 |
|  | AspG036109 | 5.3923 | 6.3947 | 9.0601 | 1.6243 | 4.8488 | 8.0363 |
|  | AspG043894 | 5.4474 | 0.2939 | 0.8370 | 0.5953 | 0.2323 | 0.3790 |
|  | AspG032019 | 10.2841 | 2.5484 | 2.1790 | 2.6452 | 3.4999 | 2.2587 |
|  | AspG031832 | 13.2547 | 2.9185 | 3.1191 | 1.5618 | 0.8729 | 0.6778 |
|  | AspG025385 | 13.3042 | 2.1952 | 1.6740 | 0.8422 | 0.6658 | 0.6130 |
|  | AspG016802 | 22.9123 | 88.2987 | 61.8564 | 16.4140 | 29.2620 | 13.9539 |
|  | AspG011877 | 63.0709 | 5.2779 | 16.4167 | 24.5758 | 19.1446 | 30.6551 |
|  | AspG003998 | 91.2655 | 11.1078 | 8.5924 | 6.1714 | 2.3755 | 1.6499 |
|  | AspG002496 | 438.3345 | 73.8443 | 58.7623 | 3.0454 | 1.8124 | 2.9092 |
| ERF | AspG001410 | 0.0765 | 2.2415 | 0.4478 | 5.4556 | 12.2800 | 6.0686 |
|  | AspG008335 | 0.1687 | 8.9645 | 12.0809 | 2.2772 | 4.9881 | 1.1540 |
|  | AspG041310 | 0.2226 | 1.4442 | 1.4069 | 4.7060 | 5.9198 | 11.2174 |
|  | AspG050569 | 0.2282 | 1.1503 | 0.6853 | 3.0023 | 4.7208 | 1.0822 |
|  | AspG043654 | 0.2417 | 0.8541 | 1.1636 | 2.3492 | 3.4375 | 5.1344 |
|  | AspG041805 | 0.4080 | 0.5190 | 0.5805 | 2.5977 | 4.3970 | 0.3756 |
|  | AspG022356 | 0.4698 | 9.8057 | 0.9022 | 19.0545 | 47.9080 | 18.5989 |
|  | AspG032121 | 0.8182 | 4.1712 | 2.7033 | 2.1430 | 4.0533 | 3.9594 |
|  | AspG008711 | 0.8956 | 4.7631 | 3.3861 | 0.2389 | 0.1354 | 0.2907 |
|  | AspG026853 | 1.0178 | 2.5606 | 8.2510 | 1.4669 | 1.0088 | 1.0465 |
|  | AspG024028 | 1.2023 | 6.5961 | 3.4580 | 2.3022 | 3.8373 | 1.0539 |
|  | AspG034223 | 1.4816 | 12.1484 | 3.5465 | 4.9375 | 10.5294 | 10.3478 |
|  | AspG033575 | 2.3839 | 0.6129 | 0.8729 | 2.0597 | 0.7465 | 0.3046 |
|  | AspG017267 | 2.5675 | 6.7482 | 19.3753 | 2.6392 | 4.0613 | 2.8373 |
|  | AspG010905 | 2.9624 | 5.8746 | 2.7930 | 0.7787 | 1.5010 | 4.9857 |
|  | AspG056183 | 3.2777 | 3.8985 | 0.6512 | 1.2073 | 1.1679 | 0.5476 |
|  | AspG027252 | 3.3619 | 1.5825 | 0.7383 | 0.7574 | 0.4086 | 0.6704 |
|  | AspG035622 | 3.9635 | 8.6342 | 14.1453 | 4.2363 | 8.2908 | 2.0041 |
|  | AspG001200 | 4.1857 | 0.6518 | 1.0640 | 0.0000 | 0.0000 | 0.0000 |
|  | AspG012280 | 4.8734 | 59.8246 | 95.6478 | 3.7687 | 4.4739 | 1.9568 |
|  | AspG002817 | 5.1385 | 1.0658 | 6.0316 | 3.3494 | 6.1646 | 0.8801 |
|  | AspG056458 | 5.3553 | 45.3171 | 25.9614 | 21.0382 | 45.6092 | 51.0707 |
|  | AspG055394 | 8.9432 | 2.8175 | 1.8521 | 0.4339 | 0.9192 | 0.0296 |
|  | AspG056196 | 9.0915 | 1.1912 | 2.2047 | 3.6248 | 3.2345 | 0.0554 |
|  | AspG019691 | 21.4534 | 70.3762 | 87.1262 | 9.9238 | 23.5550 | 28.2563 |
|  | AspG056197 | 34.1148 | 1.6398 | 1.3286 | 6.0758 | 3.3462 | 0.0241 |
|  | AspG047852 | 41.1798 | 57.0645 | 43.6545 | 4.8144 | 16.3880 | 32.1016 |
|  | AspG002258 | 107.2034 | 45.5753 | 50.4725 | 10.8524 | 8.3154 | 1.7583 |
|  | AspG019215 | 174.8405 | 222.8849 | 154.8317 | 0.0524 | 1.5895 | 0.4932 |
|  | AspG037389 | 215.9351 | 41.2805 | 94.1343 | 0.1874 | 0.0578 | 0.0083 |
|  | AspG050438 | 268.6832 | 40.0947 | 18.9068 | 78.6814 | 115.3063 | 84.3250 |
|  | AspG007446 | 340.7479 | 61.2330 | 26.4020 | 2.9747 | 7.5103 | 9.6422 |
|  | AspG007447 | 513.4708 | 63.9672 | 35.9324 | 90.0970 | 122.2925 | 42.5484 |
